# Proteomic Identification of ADAM12 as a Regulator for TGF-β1-Induced Differentiation of Human Mesenchymal Stem Cells to Smooth Muscle Cells

**DOI:** 10.1371/journal.pone.0040820

**Published:** 2012-07-13

**Authors:** Young Mi Kim, Jaeyoon Kim, Soon Chul Heo, Sang Hun Shin, Eun Kyoung Do, Dong-Soo Suh, Ki-Hyung Kim, Man-Soo Yoon, Taehoon G. Lee, Jae Ho Kim

**Affiliations:** 1 Medical Research Center for Ischemic Tissue Regeneration, School of Medicine, Pusan National University, Yangsan, Republic of Korea; 2 Department of Physiology, School of Medicine, Pusan National University, Yangsan, Republic of Korea; 3 Department of Obstetrics and Gynecology, School of Medicine, Pusan National University, Yangsan, Republic of Korea; 4 NovaCell Technology Inc., Pohang, Republic of Korea; University of Pittsburgh, United States of America

## Abstract

**Background:**

Transforming growth factor-β1 (TGF-β1) induces the differentiation of human adipose tissue-derived mesenchymal stem cells (hASCs) into smooth muscle cells. Lipid rafts are cholesterol-rich microdomains in cell membranes that reportedly play a key role in receptor-mediated signal transduction and cellular responses. In order to clarify whether lipid rafts are involved in TGF-β1-induced differentiation of hASCs into smooth muscle cells, we analyzed the lipid raft proteome of hASCs.

**Methods and Results:**

Pretreatment of hASCs with the lipid raft disruptor methyl-β-cyclodextrin abrogated TGF-β1-induced expression of α-smooth muscle actin, a smooth muscle cell marker, suggesting a pivotal role of lipid rafts in TGF-β1-induced differentiation of hASCs to smooth muscle cells. Sucrose density gradient centrifugation along with a shotgun proteomic strategy using liquid chromatography-tandem mass spectrometry identified 1002 individual proteins as the lipid raft proteome, and 242 of these were induced by TGF-β1 treatment. ADAM12, a disintegrin and metalloproteases family member, was identified as the most highly up-regulated protein in response to TGF-β1 treatment. TGF-β1 treatment of hASCs stimulated the production of both ADAM12 protein and mRNA. Silencing of endogenous ADAM12 expression using lentiviral small hairpin RNA or small interfering RNA abrogated the TGF-β1-induced differentiation of hASCs into smooth muscle cells.

**Conclusions:**

These results suggest a pivotal role for lipid raft-associated ADAM12 in the TGF-β1-induced differentiation of hASCs into smooth muscle cells.

## Introduction

Mesenchymal stem cells (MSCs) have self-renewal ability, long-term viability, and the potential to differentiate into diverse cells types, including adipogenic, osteogenic, chondrogenic, and myogenic lineages [1]–[4]. Accumulating evidence demonstrates that MSCs reside in a perivascular location found within multiple human organs, including adipose tissue [5], [6]. Pericytes, which contribute to vascularization, natively express the phenotypic and functional characteristics of MSCs [7], suggesting a key role for MSCs in vascularization. Smooth muscle cells (SMCs) have also been implicated in vascular development as well as a variety of cardiovascular diseases, including hypertension and atherosclerosis [8], [9]. The phenotype of SMCs is characterized by expression of smooth muscle-specific contractile proteins such as α-smooth muscle actin (α-SMA), *h*_1_-calponin, smoothelin, and smooth muscle-myosin heavy chain [9].

Transforming growth factor-β (TGF-β) family cytokines have been implicated in a variety of cellular responses, such as proliferation, differentiation, and apoptosis [10], [11]. TGF-β signals by binding to its type I and type II Ser/Thr kinase receptors on the cell surface, which leads to the formation of a heteromeric complex. The binding of TGF-β to type II receptor induces the recruitment and phosphorylation of TGF-β type I receptor, which phosphorylates the receptor-regulated Smads, Smad2 and Smad3. Once phosphorylated, Smad2 and Smad3 associate with Smad4 and translocate to the nucleus where they regulate the expression of TGF-β target genes. [12]. *In vitro*, MSCs have been shown to differentiate into SMCs in response to TGF-β [13], [14]. Moreover, injected bone marrow-derived MSCs have been reported to differentiate into SMCs and to contribute to the remodeling of vasculature *in vivo* [15]–[17]. We previously demonstrated that the expression of SMC-specific markers is up-regulated in human adipose tissue-derived mesenchymal stem cells (hASCs) by treatment with TGF-β1 and sphingosylphosphorylcholine [18], [19], suggesting a potential role for hASCs as SMC progenitors.

ADAM12 (a disintegrin and metalloprotease 12 or meltrin-α) is a multidomain type I transmembrane protein that functions both in normal physiology and in diseases such as cancer and osteoarthritis [20]. ADAM12 is expressed as two splice variants, a long transmembrane form (ADAM12-L) and a short secreted form (ADAM12-S). Both ADAM12-L and ADAM12-S are proteolytically processed, and the mature forms translocate to the plasma membrane and extracellular space, respectively. Full-length ADAM12-L (∼120 kDa) is processed in the trans-Golgi network to an ∼90 kDa mature form, which is the predominant form present at the cell surface [21]. ADAM12 is an active metalloprotease, that cleaves IGFBP-3 and -5 and mediates the ectodomain shedding of pro-EGF [22], [23]. ADAM12 interacts with the extracellular domain of type II TGF-β receptor and enhances TGF-β signaling by inducing accumulation of the receptor in early endosomes [24]. TGF-β1 stimulates ADAM12 expression in various cell types, including fibroblasts, hepatic stellate cells, and epithelial cells [25], [26]. However, the role of ADAM12 in TGF-β1-induced differentiation of MSCs to SMCs has not been determined.

Many receptors and their downstream signaling molecules are reported to be organized into specific membrane compartments, called lipid rafts [27], [28]. Lipid rafts are dynamic membrane domains enriched with cholesterol and sphingolipids that are characterized by their insolubility in nonionic detergents and low density [29]–[31]. In addition to their role in signaling, lipid rafts are also involved in various cellular functions such as endocytosis, pathogenic invasion, immune responses, and cellular migration [32]. Caveolae are plasma membrane invaginations or lipid rafts containing caveolin-1, a protein that is highly expressed in endothelial cells, muscle cells, fibroblasts, and adipocytes [32]. TGF-β receptors are distributed in both lipid rafts/caveolae and non-raft membrane microdomains. Proteomics technologies are very useful for the identification of lipid raft-associated protein components [33] and the elucidation of the biological functions of these proteins during biological responses, including TGF-β1-induced differentiation of MSCs.

To explore the role of lipid rafts in TGF-β1-induced differentiation of hASCs into SMCs, we characterized the molecular profiles of lipid rafts using a shotgun proteomic technique and subsequently identified ADAM12 as a TGF-β1-induced and lipid raft-associated protein. We then explored the role of ADAM12 in TGF-β1-induced differentiation of hASCs into SMCs.

## Materials and Methods

### Materials

Trypsin, α-minimum essential medium, fetal bovine serum, Alexa Fluor 488 goat anti-mouse antibody, Alexa Fluor 568 goat anti-rabbit antibody, and Lipofectamine™ 2000 reagent were purchased from Invitrogen (Carlsbad, CA, www.invitrogen.com). Methyl-β-cyclodextrin (MβCD), filipin, anti-ADAM-12 antibody and anti-α-SMA antibody were obtained from Sigma-Aldrich (St. Louis, MO, www.sigmaaldrich.com). Anti-phospho-Smad2, anti-Smad2, and anti-calnexin antibodies were purchased from Cell Signaling Technology (Danvers, MA, www.cellsignal.com). Anti-caveolin-1 and anti-flotillin-1 antibodies were purchased from BD Biosciences (Franklin Lakes, NJ, www.bd.com). Anti-glyceraldehyde-3-phosphate dehydrogenase (GAPDH) was purchased from Millipore (Temecula, CA, www.milliore.com). Human recombinant TGF-β1 was purchased from R&D systems, Inc. (Minneapolis, MN). Vectashield mounting medium with 4′-6-Diamidino-2-phenylindole (DAPI) was purchased from Vector Laboratories (Burlingame, CA, www.vectorlabs.com). Culture plates were purchased from Nunc (Roskilde, Denmark, www.nuncbrand.com). Peroxidase-labeled secondary antibodies and enhanced chemiluminescence kit were obtained from Amersham Biosciences (Pittsburgh, PA, www4.gelifesciences.com).

### Cell Culture

Subcutaneous adipose tissue was acquired from patients during elective surgery after obtaining each patient’s written consent and following approval by the Institution Review Board of Pusan National University Hospital. For the isolation of hASCs, adipose tissues were washed at least 3 times in sterile PBS and treated with an equal volume of collagenase type I suspension (1 g/L of HBSS buffer with 1% bovine serum albumin) for 60 min at 37°C with intermittent shaking. The floating adipocytes were separated from the stromal-vascular fraction by centrifugation at 300×*g* for 5 min. The cell pellet was resuspended in α-minimum essential medium supplemented with 10% fetal bovine serum, 100 U/mL penicillin, and 100 µg/mL streptomycin, and the cells were then plated in tissue culture dishes at 3500 cells/cm^2^. The primary hASCs were cultured for 4–5 days until they reached confluence and this was defined as passage “0”. The passage number of hASCs used in these experiments was 3–10. The hASCs were positive for CD29, CD44, CD73, CD90, and CD105, whereas they were negative for CD31, CD34, and CD45. To induce differentiation of hASCs into SMCs, hASCs were seeded onto tissue culture dishes, serum-starved for 24 h, and then treated with serum-free medium in the presence or absence of 2 ng/mL TGF-β1 for 4 days. TGF-β1-induced differentiation of hASCs into SMCs was confirmed by measuring α-SMA expression by Western blotting.

### Purification of Caveolin-1-enriched Lipid Raft Fractions

Caveolin-enriched lipid raft fractions were prepared as described previously [34] with a slight modification. Serum-starved hASCs were treated with serum-free medium in the presence or absence of 2 ng/mL TGF-β1 for 4 days. The cells (three 150-mm dishes per experimental condition) were washed once in ice-cold phosphate-buffered saline and scraped into 2 mL of MES buffer (25 mM MES, pH 6.5, 1 mM EGTA, and 150 mM NaCl) containing 1% Triton X-100, 1 mM phenylmethylsulfonyl fluoride, and 1 µg/mL leupeptin. Lysates were incubated for 10 min on ice with frequent agitation. Homogenization was carried out with 10 strokes of a loose fitting Dounce homogenizer, and the protein concentration of homogenate was determined by the Bradford method [35]. The sucrose concentration of each homogenate was adjusted to 40% by adding 80% sucrose prepared in MES-buffered saline. Equal amounts of protein were then placed at the bottom of an ultracentrifuge tube, and 4 mL of MES-buffered saline containing 30% sucrose and 5% sucrose were successively layered on top of the sample. The tubes were centrifuged at 39,000 rpm in an SW41 rotor for 12 h at 4°C. A total of 12 fractions (1 mL each) were collected beginning from the top of the gradient. An equal volume of each gradient fraction was separated by SDS-PAGE and subjected to Western blot analysis.

### Cholesterol Depletion and Measurement of Cholesterol Levels

To disrupt lipid raft membrane domains, membrane cholesterol was depleted by treating the hASCs with serum-free medium containing 5 mM MβCD for 1 h at 37°C. Cellular cholesterol levels were assayed spectrophotometrically using an Amplex Red cholesterol assay kit (Invitrogen) according to manufacturer’s protocol. In order to verify that the MβCD treatment depleted membrane cholesterol, filipin, a cholesterol-binding polyene antibiotic, was used to stain membrane-associated cholesterol. Control or MβCD-treated cells were fixed with 3.7% formaldehyde in phosphate-buffered saline for 10 min. After rinsing, the cells were stained with phosphate-buffered saline containing 50 µg/mL filipin and 0.1% bovine serum albumin for 30 min at room temperature. Fluorescence images were obtained using an inverted fluorescence microscope (Leica DM IRB, Solms, Germany) mounted with a digital camera using the following filter: excitation 360/40 nm and emission 460/50 nm.

### Sample Preparation

Lipid raft fractions were treated with 10% trichloroacetic acid and incubated for 10 min at 4°C, followed by centrifugation at 14,000 rpm for 10 min to precipitate proteins. The supernatant was removed and the pellets were washed 3 times with 1 mL cold acetone and then dried to remove residual acetone. Lipid raft proteomes were hydrolyzed at aspartic acid residue using ChemDigest™ (NOVACELL Technology, Korea). The resulting hydrolysate was lyophilized, reconstituted by water/0.1% TFA and subjected to chromatographic separation by off-line C18 HPLC. Chromolith RP-18 Endcapped column (Merck, Height: 100 mm, Diameter : 4.6 mm) was equilibrated with water/0.1% TFA. A 0%–50% gradient of acetonitrile/0.1% TFA was applied over 7 min, and 50%–100% applied for the next 1 min at a flow rate of 3 ml/min. HPLC eleunts were collected to give 6 fractions. Each fraction was lyophilized and dissolved with 50 µl of 50 mM ammonium bicarbonate. Each sample was incubated at 90°C for 15 min, and then 5 µL of 10 mM dithiothreitol was added and the samples were incubated at 56°C for 20 min. Next, 5 µL of 100 mM iodoacetamide was added and incubated in the dark at room temperature for 15 min. To consume any unreacted iodoacetamide, an additional 5 µL of 100 mM dithiothreitol was added. Reduced and alkylated proteins were digested with trypsin (Promega, Madison, WI) at 37°C for 12 h.

### LC-MS/MS

All mass analyses were performed using a nano-LC MS system consisting of an Agilent 1100 high-pressure liquid chromatography (HPLC) system (Agilent Technologies, Santa Clara, CA) and a QSTAR quadrupole-time-of-flight mass spectrometer (MDS SCIEX, Concord, Ontario, Canada) equipped with a nano-electrospray ionization source. To achieve high-resolution separation, a nanoscale reversed phase chromatography analytical column (ZORBAX C18, 0.1 mm, 0.075 mm i.d.; Agilent Technologies) was used. Mobile phase A consisted of HPLC-grade water containing 0.1% formic acid, and mobile phase B consisted of 84% HPLC grade acetonitrile containing 0.1% formic acid. Separation was performed at a flow rate of 250 nL/min and the applied gradient was 0%–40% phase B over 60 min. For MS/MS analysis, each scan cycle consisted of 1 full scan mass spectrum (m/z 400–1500) followed by 3 MS/MS events. Dynamic exclusion was activated for 1 min with a repeat count of 2. Digested samples were run in duplicate and representative LC-MS/MS data from three independent experiments are shown (Table S1).

### Database Searching

The LC-MS/MS data were used to search the UniProt database (v15.10) using in-house MASCOT software (ver 2.2.04). The following parameters were used: 6 missed cuts; carbamidomethylation (C) as a fixed modification; *N*-acetyl (protein), oxidation (M), and pyroglutamylation (N-term EQ) as variable modifications; and charge states +2, +3, and +4. Windows of mass accuracy of 100 ppm and 0.25 Da were used for precursor ions and MS/MS data, respectively. Peptide identification and protein assembly were performed in multiple stages. Initial peptide filtering was used to determine the estimated 1% false discovery rate, which was calculated using the target-decoy method [36]. All protein identifications required the detection of unique peptides, and proteins with more than 2 spectral counts were selected for further analysis. Proteins identified with a MASCOT score that was higher in the bovine database than in the human database were considered serum contamination and were removed.

### Quantitative Analysis of MS Results

To estimate the fold-change of identified proteins between the experimental groups, we used a label-free quantitative method based on ion intensity measurement with some modifications [37], [38]. Briefly, we used the total ion intensity (TII), which is the sum of all fragment ion intensities in an MS/MS spectrum. The values of the TII of a protein in each group (TII_CON_, TII_TGF-β1_) could be calculated by the summation of the TIIs of all spectra identified in each condition. The log_2_ ratio of TII_TGF-β1_ to TII_CON_ was calculated and used as a quantitative index. Log_2_ ratio of TIITGF-β1 and TIICON was calculated and used for quantitative index. To avoid taking logarithm on zero’s, we set the TIICON and TIITGF-β1 as 0.371 and 0.379, respectively, which is the half of the smallest value of TII if no peptide was identified in each experimental group.

### Western Blotting

Serum-starved hASCs were treated under the appropriate conditions, washed with ice-cold PBS, and then lysed in lysis buffer (20 mM Tris-HCl, 1 mM EGTA, 1 mM EDTA, 10 mM NaCl, 0.1 mM phenylmethylsulfonyl fluoride, 1 mM Na_3_VO_4_, 30 mM sodium pyrophosphate, 25 mM β-glycerol phosphate, and 1% Triton X-100; pH 7.4). Lysates were resolved by SDS-PAGE, transferred to a nitrocellulose membrane, and then stained with 0.1% Ponceau S solution (Sigma-Aldrich). After blocking in 5% nonfat milk, the membranes were immunoblotted with various antibodies, and the bound antibodies were visualized with horseradish peroxidase-conjugated secondary antibodies using the enhanced chemiluminescence Western blotting kit (ECL, Amersham Biosciences). Intensities of the protein bands on the blots were quantified using Scion Image software (http://rsb.info.nih.gov/nih-image).

### Immunocytochemistry

Immunostaining and confocal microscopy were used to determine the subcellular distribution of ADAM12 and caveolin-1. Cells were fixed in PBS containing 4% paraformaldehyde for 15 min, permeabilized with PBS containing 0.2% Triton X-100 for 10 min, and then blocked with PBS containing 2% bovine serum albumin. For immunostaining, specimens were incubated with anti-ADAM12 and anti-caveolin-1 antibodies for 2 h, followed by treatment with Alexa Fluor 488 goat anti-mouse and Alexa Fluor 568 goat anti-rabbit antibodies for 1 h. The specimens were finally washed and mounted in Vectashield medium with DAPI for visualization of nuclei. A Leica TCL-SP2 confocal microscope system was used for collection of fluorescence and differential interference contrast (DIC) images.

### Reverse Transcription-polymerase Chain Reaction (RT-PCR) Analysis

Total cellular RNA was extracted using the TRIzol method (Invitrogen, Carlsbad, CA). For RT-PCR analysis, 2 µg aliquots of each RNA were subjected to cDNA synthesis using 200 U of M-MLV reverse transcriptase and 0.5 µg of oligo (dT) 15 primer (Promega, Madison, WI). The cDNA in 2 µL of the reaction mixture was amplified using 0.5 U of GoTaq DNA polymerase (Promega, Madison, WI), and 10 pmol each of the following sense and antisense primers: ADAM12, 5′-CTGCTCCACAATATCCACAAG-3′, 5′-AGGACTCAAAAGTCTGGCCTTTCA-3′; GAPDH, 5′-TCCATGACAACTTTGGTATCG-3′, 5′-TGTAGCCAAATTCGTTGTCA-3′; α-SMA, 5′-CCTGACTGAGCGTGGCTATT-3′, 5′-GATGAAGGATGGCTGGAACA-3′. The thermal cycle was as follows: denaturation at 95°C for 30 s, annealing at 52–58°C for 30 s depending on the primers used, and extension at 72°C for 30 s. Each PCR reaction was carried out for 30 cycles, and the PCR products were size fractionated on a 1.2% agarose gel with ethidium bromide and photographed under UV transillumination.

### Gene Silencing Using Short Hairpin RNA (shRNA) Lentivirus or Small Interfering RNAs (siRNAs)

pLKO.1-puro lentiviral vectors bearing ADAM12 shRNA (TRCN0000047035) and nontarget control shRNA (SHC002) were purchased from Sigma-Aldrich. The functional sequence in the ADAM12 shRNA lentiviral vector was as follows: CCGGGCTGCCGGATTTGTGGTTTATCTCGAGATAAACCACAAATCCGGCAGCTTTTTG to target the ADAM12 gene sequence (GCTGCCGGATTTGTGGTTTAT). To generate lentiviral particles, HEK293FT cells were co-transfected with the shRNA lentiviral plasmid pLKO.1-puro and ViraPower Lentiviral packaging mix (pLP1, pLP2, pLP-VSV-G; Invitrogen) using Lipofectamine 2000 (Invitrogen) and the culture supernatants containing lentivirus were harvested 48 h after transfection. For lentiviral transduction, hASCs were treated with culture supernatants from HEK293FT cells in the presence of 5 µg/mL polybrene (Sigma-Aldrich) and stable cell lines expressing shRNA were generated by selection with puromycin (5 µg/mL). To ensure shRNA-mediated silencing of ADAM12 expression, the mRNA levels of ADAM12 and GAPDH were determined by RT-PCR analysis.

For siRNA experiments, hASCs were seeded on 60-mm dishes. At 70% confluence, the cells were transfected with the appropriate siRNAs using Lipofectamine 2000 reagent according to manufacturer’s instructions (Invitrogen). ADAM12 siRNA duplexes were synthesized, desalted, and purified by Samchully Pharm. Co. Ltd. (Siheung, GyeongGi, Korea) with the following sequences: 5′-AACGGGAAAGCAAAGAACUTT-3′ (sense) and 5′-AGUUCUUUGCUUUCCCGUUTT-3′ (anti-sense). Nonspecific control siRNA was purchased from Dharmacon (Lafayette, CO). Lipofectamine 2000 reagent was incubated in serum-free medium for 10 min, and the respective siRNAs were then added into the mixture. After incubation at room temperature for 15 min, the mixture was diluted with serum-free medium to obtain a final concentration of 100 nM siRNAs in each well. After the hASCs were incubated with the siRNAs for 6 h, the cells were cultured in growth medium for 24 h, and the expression of ADAM12 and GAPDH was determined by Western blotting.

### Statistical Analysis

The results of multiple observations are presented as mean ± SD. For multivariate data analysis, group differences were assessed with 2-way ANOVA, followed by post hoc comparisons tested using Scheffe’s method.

## Results

### Cholesterol Depletion Abrogates TGF-β1-induced α-SMA Expression and Smad2 Phosphorylation in hASCs

**Figure 1:**
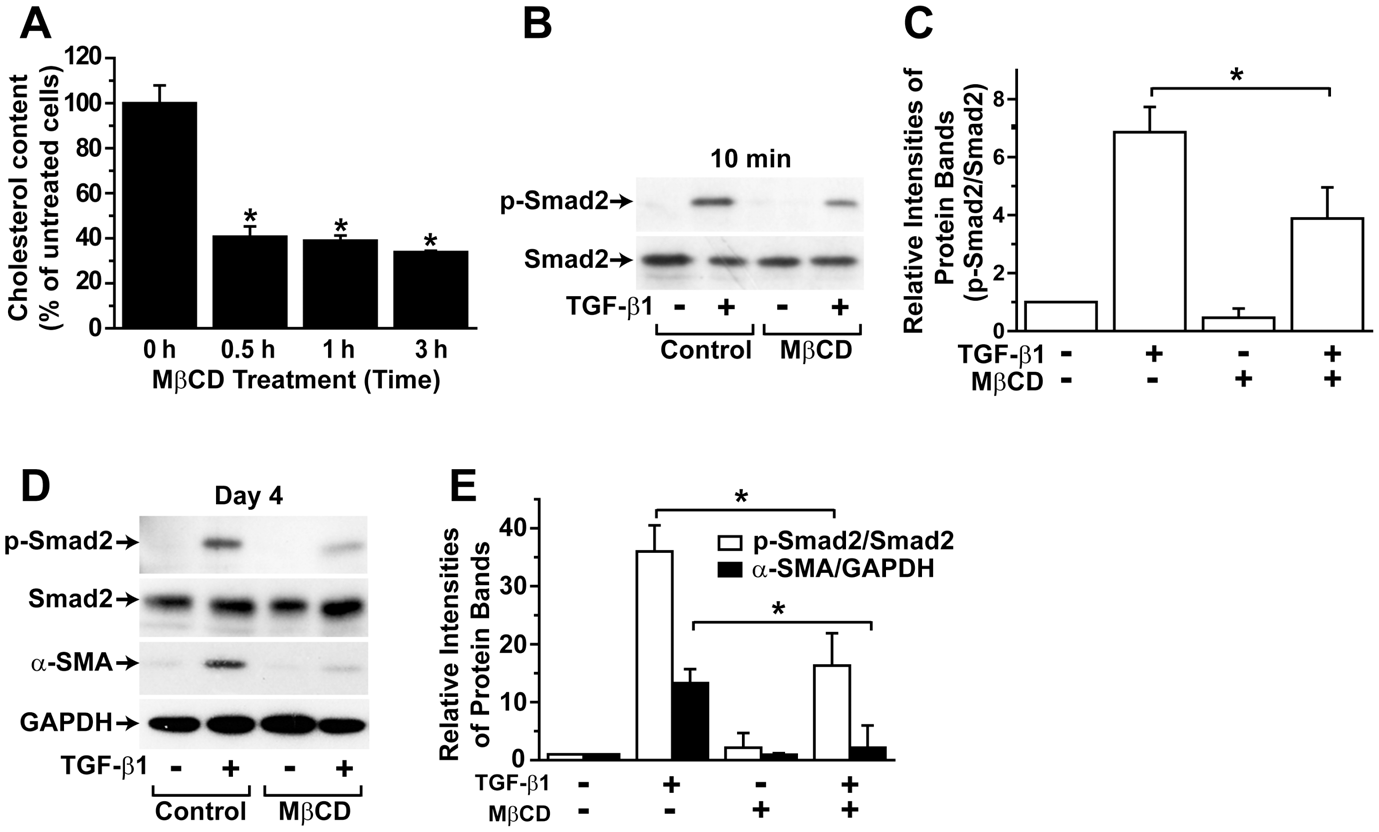
Effects of cholesterol depletion on TGF-β1-stimulated α-SMA expression and Smad2 phosphorylation. (A) Effects of MβCD on cellular cholesterol content. hASCs were treated with serum-free medium containing 5 mM MβCD for the indicated time periods, and the cellular cholesterol content of hASCs was determined. (B, D) Effects of cholesterol depletion on TGF-β1-induced Smad2 phosphorylation. hASCs were pretreated with serum-free medium in the presence or absence of 5 mM MβCD for 30 min, then treated with TGF-β1 for either 10 min (B) or 4 days (D). The expression and phosphorylation of Smad2 and the expression of α-SMA and GAPDH were determined by Western blotting. (C) The relative intensities of p-Smad2 vs. Smad2 from panel (B) are shown. (E) The relative intensities of p-Smad2 vs. Smad2 and α-SMA vs. GAPDH from panel (D) are shown. Representative data from three independent experiments are shown. * *p*<0.05 by 2-way ANOVA followed by post hoc comparisons using Scheffe’s method.

MβCD has been reported to selectively extract membrane cholesterol and disrupt lipid rafts [39]. In order to explore whether lipid rafts are involved in TGF-β1-induced cellular responses, hASCs were treated with 5 mM MβCD to disrupt lipid rafts by cholesterol depletion. Treatment of hASCs with 5 mM MβCD for 30 min diminished cholesterol by approximately 60% (Fig. 1A). Cholesterol depletion from the plasma membrane was confirmed by staining with filipin, a fluorescent polyene antibiotic that binds to cholesterol and is used as a cholesterol probe [40]. MβCD treatment reduced filipin binding in a time-dependent manner (Figure S1).

To study the influence of cholesterol depletion on TGF-β1-stimulated signal transduction, hASCs were pre-treated with 5 mM MβCD for 30 min, and treated with 1 ng/mL TGF-β1 or vehicle for 10 min, followed by measurement of Smad2 phosphorylation. TGF-β1-induced acute phosphorylation of Smad2 within 10 min was markedly attenuated by pretreatment of the cells with MβCD (Fig. 1B and 1C), suggesting a possible lipid raft-dependent mechanism for TGF-β1-induced Smad2 phosphorylation.

To assess whether lipid rafts are involved in TGF-β1-induced differentiation of hASCs into SMCs, we examined the effects of MβCD on TGF-β1-stimulated α-SMA expression. hASCs were treated with TGF-β1 in the presence or absence of 5 mM MβCD for 4 days. As shown in Fig. 1D and 1E, TGF-β1-induced α-SMA expression was blocked by pretreatment of cells with MβCD. Furthermore, TGF-β1 induced sustained Smad2 phosphorylation on day 4 which was markedly attenuated by pretreatment with MβCD.

### Proteomic Identification of TGF-β1-induced Proteins in Lipid Raft Fractions of hASCs

To identify TGF-β1-induced lipid raft-associated proteins, hASCs were treated with serum-free medium in the presence or absence of 1 ng/mL TGF-β1 for 4 days and lipid raft fractions were isolated using sucrose density gradient centrifugation. Lipid raft fractions were subjected to LC-MS/MS analyses for protein identification. For comparative analysis of the lipid raft proteome associated with TGF-β1-induced differentiation, a label-free quantitative approach was adopted, and the determination of relative abundance was based on the sum of the total ion intensity of peptides matched in the mock- and the TGF-β1-treated cells. After single peptide matched proteins were excluded, 1002 proteins were identified from the comparative proteomic analysis of lipid raft fractions. The identities and relative abundance of proteins in the lipid raft proteome are summarized in Table S1. The subcellular localization and molecular function of the proteome are summarized in the Table S2. We classified 242 features that had Log_2_ ratio values are higher than 1 as TGF-β1-induced proteins (Table S1). The functional annotation analysis of the TGF-β1-induced proteins based on gene ontology terms was performed using DAVID 2008, and the complete list of gene ontology annotation is shown in Table S3.

A literature mining for lipid raft proteins in the PubMed database revealed 517 proteins that were suggested as lipid raft-associated proteins [41]. A comparison with the database for lipid raft proteins showed that 97 features of the lipid raft proteome of hASCs were previously reported as lipid raft-associated proteins (Table S4). An abbreviated list of the lipid raft-associated proteins is presented in Table 1. These proteins include well-known lipid raft markers, such as caveolin-1, caveolin-2, flotillin-1, and flotillin-2, which were not up-regulated in response to TGF-β1 treatment. In addition, we found that 3 ADAM family proteases (ADAM12, ADAM10, and ADAM17), low-density lipoprotein receptor, syndecan-1, and β-catenin were lipid raft-associated proteins. ADAM12 was identified as the protein that was most highly up-regulated in response to TGF-β1 treatment. A representative mass spectrum of ADAM12 is shown in Figure S2.

**Table 1:** An abbreviated list of TGF-β1-induced lipid raft-associated proteins in hASCs.

Gene	Uniprot Acc^a^	Protein	Log_2_ Ratio^b^
ADAM12	O43184	Disintegrin and metalloproteinase domain-containing protein 12	6.233
LDLR	P01130	Low-density lipoprotein receptor	4.732
PKD2	Q13563	Polycystin-2	4.435
SDC1	P18827	Syndecan-1	3.579
FAS	P25445	Tumor necrosis factor receptor superfamily member 6	3.355
CTNNB1	P35222	Catenin beta-1	2.448
ADAM10	A0AV88	Disintegrin and metalloproteinase domain-containing protein 10	2.378
KIDINS220	Q9ULH0	Kinase D-interacting substrate of 220 kDa	2.181
DYSF	O75923	Dysferlin	1.879
CD99	P14209	CD99 antigen	1.588
ATP1B1	P05026	Sodium/potassium-transporting ATPase subunit beta-1	1.397
NCSTN	Q92542	Nicastrin	1.300
ITGAV	A5YM53	ITGAV protein	1.027
CALR	P27797	Calreticulin	1.022
CD47	Q08722	Leukocyte surface antigen CD47	1.006
RAP1A	P62834	Ras-related protein Rap-1A	1.003
ADAM17	P78536	Disintegrin and metalloproteinase domain-containing protein 17	0.758
FLOT1	O75955	Flotillin-1	0.700
FLOT2	Q14254	Flotillin-2	−0.332
CAV2	P51636	Caveolin-2	−0.987
CAV1	Q03135	Caveolin-1	−1.638

a Uniprot accession number.

b Log_2_(TII_TGF-β1_/TII_con_).

### TGF-β1-stimulated Expression of ADAM12 in hASCs

**Figure 2:**
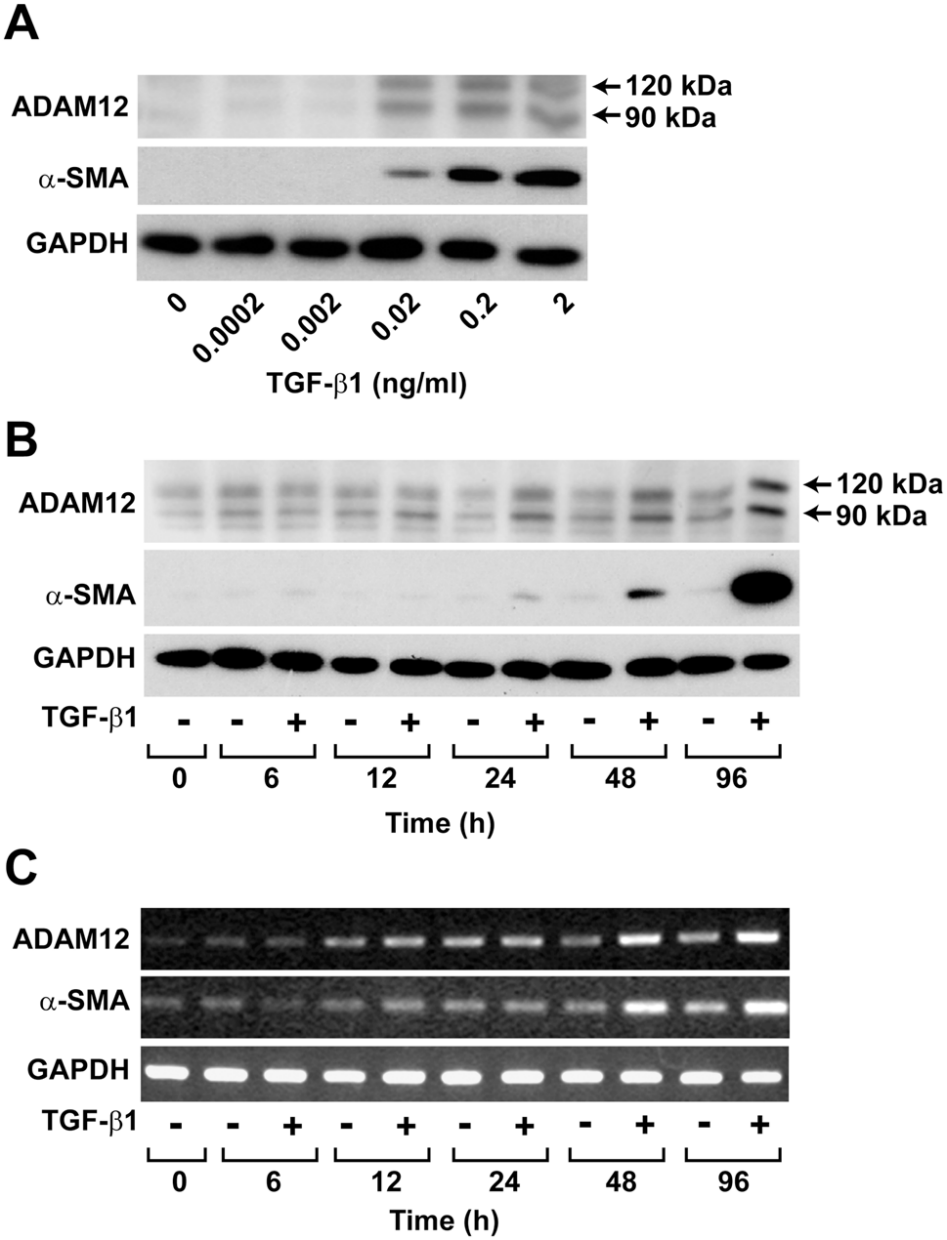
Effects of TGF-β1 on the expression of ADAM12 and α-SMA in hASCs. (A) Dose-dependent effects of TGF-β1 on the expression of ADAM12 and α-SMA. hASCs were treated with the indicated concentrations of TGF-β1 for 4 days. (B) Time dependence of TGF-β1-induced expression of ADAM12 and α-SMA. hASCs were treated with serum-free medium containing 1 ng/mL TGF-β1 or vehicle (0.1% BSA) for the indicated time periods. The expression levels of α-SMA, ADAM12, and GAPDH were determined by Western blotting. The ADAM12 protein bands with molecular masses of ∼120 kDa and ∼90 kDa are indicated. (C) TGF-β1-induced mRNA expression of ADAM12 and α-SMA. hASCs were treated with serum-free medium containing 1 ng/mL TGF-β1 or vehicle (0.1% BSA) for the indicated time periods. The expression levels of α-SMA, ADAM12, and GAPDH were determined by RT-PCR analysis. Representative data from three independent experiments are shown.

To confirm the proteomic identification of ADAM12 as a TGF-β1-induced protein in hASCs, the effect of TGF-β1 on ADAM12 expression was assessed by Western blotting. TGF-β1 dose-dependently increased the expression levels of not only α-SMA but also ADAM12 (Fig. 2A). Consistent with a previous report [21], Western blotting of cell lysates identified two protein bands for ADAM12 at ∼120 and ∼90 kDa. In addition, TGF-β1 treatment time-dependently stimulated expression of α-SMA, and the two ADAM12 bands increased to a maximum on day 4 (Fig. 2B). Likewise, mRNA levels of ADAM12 and α-SMA were also up-regulated by TGF-β1 treatment in a time-dependent manner (Fig. 2C).

### Localization of ADAM12 in Lipid Raft Fractions of hASCs

**Figure 3:**
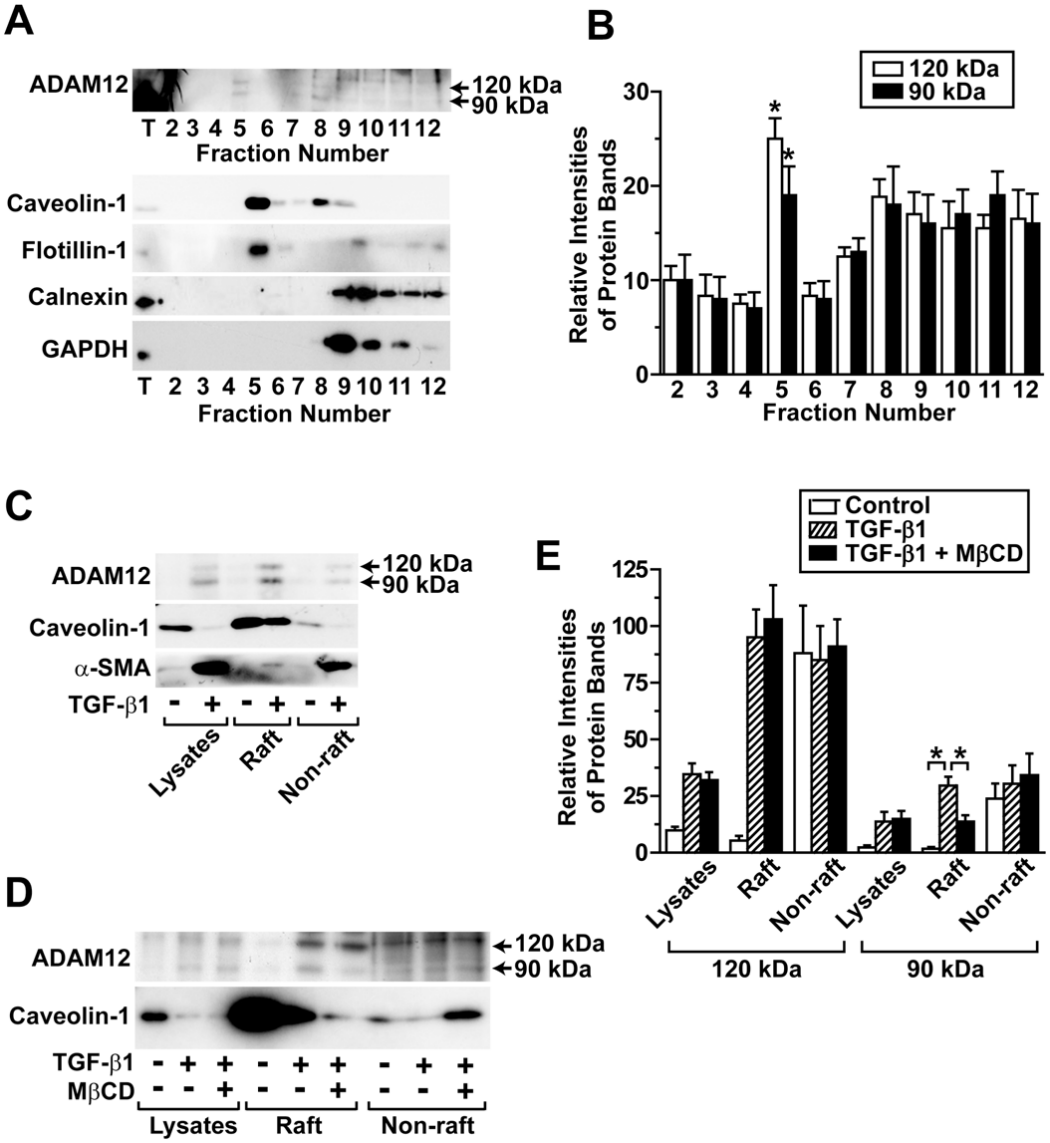
TGF-β1-stimulated expression of ADAM12 in lipid raft fractions of hASCs. (A) Existence of ADAM12 in lipid raft fractions. hASCs were treated with 1 ng/mL TGF-β1 for 4 days and lipid rafts were fractionated by sucrose density gradient centrifugation. The protein levels of ADAM12, caveolin-1, flotillin-1, calnexin, and GAPDH in each fraction were probed by Western blot analysis. (B) The relative intensities of ADAM12 protein bands with molecular masses of ∼120 kDa and ∼90 kDa from the panel (A) were quantified. * *p*<0.05 vs fraction number 2. (C) TGF-β1-stimulated expression of ADAM12 in lipid raft fractions. hASCs were treated with serum-free medium in the presence or absence of 1 ng/mL TGF-β1 for 4 days, and then lipid rafts were fractionated. (D) Effects of cholesterol depletion on TGF-β1-stimulated expression of ADAM12 proteins in lipid raft fractions. hASCs were treated with serum-free medium in the presence or absence of 1 ng/mL TGF-β1 for 4 days. TGF-β1-treated hASCs were subsequently treated with 5 mM MβCD or vehicles for 30 min. The expression levels of ADAM12, caveolin-1, and α-SMA in cell lysates, the lipid raft fraction (#5), and pooled non-lipid raft fractions (sample pooled from #8–12) were determined by Western blotting. Representative data from three independent experiments are shown. (E) The relative intensities of ADAM12 protein bands with molecular masses of ∼120 kDa and ∼90 kDa from the panel (D) were quantified. * *p*<0.05 by 2-way ANOVA followed by post hoc comparisons using Scheffe’s method.

To determine the existence of ADAM12 in lipid raft fractions of hASCs, the ADAM12 protein levels in sucrose density gradient fractions from hASCs were determined by Western blotting. As shown in Fig. 3A and 3B, ADAM12 could be detected in both lipid raft (fraction 5) and non-lipid raft fractions (fractions 8–12) in TGF-β1-treated cells. ADAM12 co-localized with lipid raft-specific markers, including caveolin-1 and flotillin-1, in the lipid raft fraction. The lipid raft fraction appears to be relatively free of contamination by endoplasmic reticulum membranes and cytosol, as evidenced by the absence of calnexin and GAPDH from fraction 5. TGF-β1 treatment decreased the expression of caveolin-1 in hASCs, which was consistent with the proteomic data suggesting that caveolin-1 levels in lipid raft fractions decreased in response to TGF-β1 treatment (Fig. 3C). In total lysates from TGF-β1-treated hASCs, ADAM12 expression increased compared with that in control cell lysates. TGF-β1 treatment increased ADAM12 protein levels not only in lipid raft fractions but also in non-lipid raft fractions.

Because MβCD treatment blocked TGF-β1-stimulated Smad2 phosphorylation and α-SMA expression, we explored the effect of MβCD on the localization of ADAM12 in lipid rafts. hASCs were treated with TGF-β1 for 4 days, then treated with 5 mM MβCD for 30 min, and lipid rafts were fractionated. Treatment with MβCD for 30 min attenuated the levels of caveolin-1 in lipid rafts, which was in contrast to the increased caveolin-1 levels in non-lipid raft fractions, suggesting MβCD-induced disruption of lipid rafts. Levels of the ∼90 kDa ADAM12 protein in lipid raft fractions were diminished by treatment with MβCD, whereas the levels of ∼120 kDa ADAM12 protein were not significantly affected by MβCD treatment (Fig. 3D and 3E). These results support the findings that the ∼90 kDa mature form of ADAM12, but not the ∼120 kDa precursor, localizes to the lipid rafts of hASCs.

### Co-localization of ADAM12 and Caveolin-1 in hASCs

**Figure 4:**
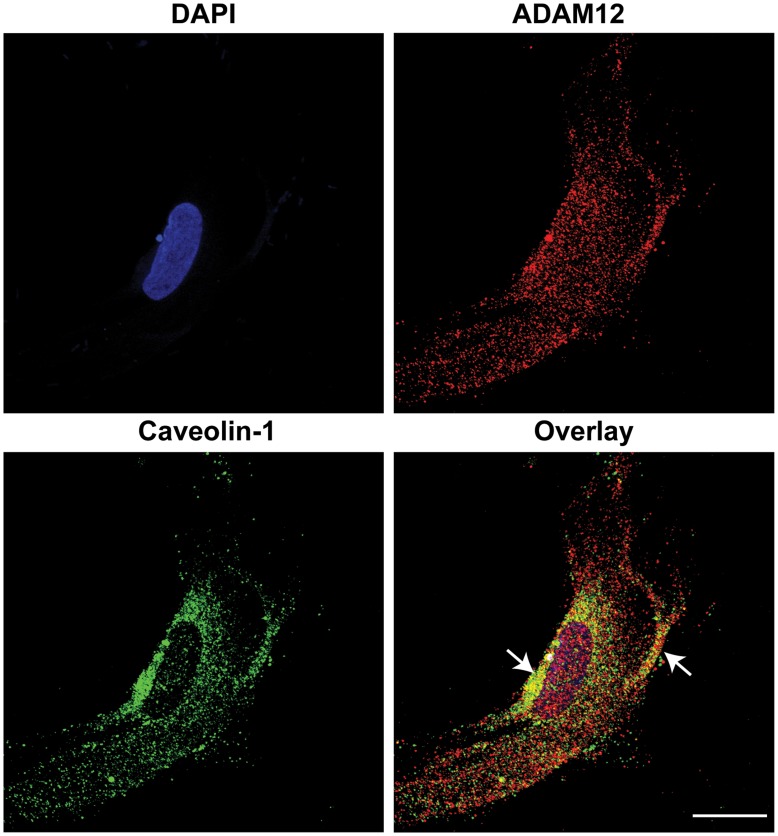
Co-localization of ADAM12 and caveolin-1 in hASCs. hASCs that overexpressed ADAM12 were immunostained with anti-ADAM12 and anti-caveolin-1 antibodies (Bar = 20 µm). Images for ADAM12 (red color) and caveolin-1 (green color) were overlaid, and arrows indicate colocalization of ADAM12 and cavelin-1. Representative data from three independent experiments are shown.

To confirm the existence of ADAM12 in caveolin-1-positive lipid raft fractions, we examined the localization of ADAM12 and caveolin-1 in hASCs using immunofluorescence staining. ADAM12 was over-expressed in hASCs by transfection with a plasmid harboring ADAM12 cDNA. ADAM12 was detected mainly in the perinuclear area of hASCs, and the ADAM12-positive signal markedly overlapped with caveolin-1 staining in plasma membrane (Fig. 4), suggesting localization of ADAM12 in caveolin-1-positive lipid rafts.

### ADAM12 is Involved in TGF-β1-induced α-SMA Expression

**Figure 5:**
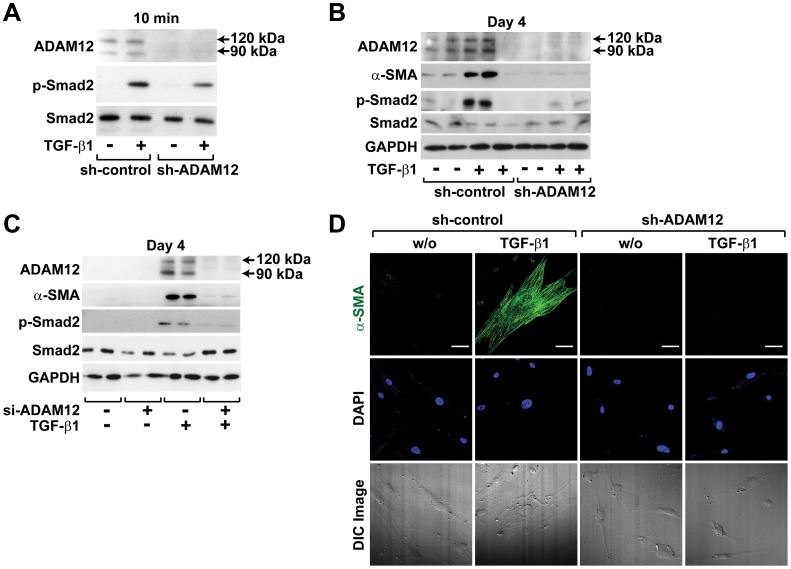
Role of ADAM12 in TGF-β1-stimulated phosphorylation of Smad2 and expression of α-SMA. (A-C) Silencing of ADAM12 expression inhibits TGF-β1-stimulated α-SMA expression and Smad2 phosphorylation. hASCs were infected with either sh-control or sh-ADAM12 lentivirus, and then treated with TGF-β1 or vehicle for either 10 min (A) or 4 days (B). (C) hASCs were transfected with si-control or si-ADAM12, and then treated with either TGF-β1 or vehicle for 4 days. The levels of ADAM12, α-SMA, Smad2, and GAPDH protein and the phosphorylation of Smad2 were determined by Western blotting. Representative data from three independent experiments performed in duplicate are shown. (D) ADAM12 dependence of TGF-β1-induced α-SMA expression. shRNA-infected hASCs were immunostained with anti-α-SMA antibody (green) and counterstained with DAPI (blue) (Bar = 50 µm). Fluorescence and corresponding differential interference contrast (DIC) images were taken by a laser scanning confocal microscope. Representative data from three independent experiments are shown.

To assess the role of ADAM12 in TGF-β1-induced differentiation of hASCs into SMCs, ADAM12 expression was silenced using lentiviral shRNA, and the effects of TGF-β1 on α-SMA expression and Smad2 phosphorylation were determined. As shown in Fig. 5A, lentiviral transduction of ADAM12 shRNA silenced endogenous expression of ADAM12. Moreover, the TGF-β1-induced acute phosphorylation of Smad2 that occurred within 10 min was significantly attenuated by silencing of endogenous ADAM12 expression, suggesting that ADAM12 is partially involved in TGF-β1-stimulated acute phosphorylation of Smad2. Depletion of ADAM12 expression abrogated TGF-β1-induced α-SMA expression on day 4. Furthermore, TGF-β1-induced Smad2 phosphorylation on day 4 was also blocked by ADAM12 depletion (Fig. 5B). To support these results, we silenced ADAM12 expression in hASCs by transfection with ADAM12-specific siRNA. As shown in Fig. 5C, siRNA-mediated depletion of ADAM12 inhibited TGF-β1-stimulated α-SMA expression and Smad2 phosphorylation on day 4. In support of these results, shRNA-mediated silencing of ADAM12 expression abrogated TGF-β1-induced α-SMA expression and stress fiber formation (Fig. 5D). These results suggest that TGF-β1-induced ADAM12 expression is required for TGF-β1-stimulated Smad2 phosphorylation and α-SMA expression.

## Discussion

Shotgun proteomic analysis using LC-MS/MS is a powerful strategy for the identification of membrane proteins associated with the differentiation of stem cells. Using sucrose density gradient fractionation and LC-MS/MS analysis, we identified 1002 features as lipid raft-associated proteins in hASCs. A total of 242 proteins were determined to be TGF-β1-induced, while 190 and 570 were classified as TGF-β1-suppressed and -independent proteins, respectively. By comparing our data with the previously reported lipid raft protein database [41], we observed that 97 out of the 1002 identified in the proteome corresponded to previously reported lipid raft-associated proteins; however, whether the other 905 features are localized in lipid rafts remains to be determined. Among the 97 lipid raft-associated features, 16 were classified as TGF-β1-induced proteins, while 17 and 64 were identified as TGF-β1-inhibited proteins and TGF-β1-independent proteins, respectively. TGF-β1-induced lipid raft proteins included ADAM12, ADAM10, ADAM17, low density lipoprotein receptor, syndecan-1, and β-catenin. Among them, ADAM12, low-density lipoprotein receptor, syndecan-1, and β-catenin were previously reported to be induced by TGF-β1 treatment [42]–[45]. From proteomic analyses and database searching, we finally identified ADAM12 as a TGF-β1-induced lipid raft protein in hASCs. Therefore, LC-MS/MS technology will be useful for the identification of lipid raft- or cell membrane-associated proteins in stem cells.

In the present study, we demonstrated for the first time that ADAM12 with a molecular mass of ∼90 kDa, but not ∼120 kDa, is primarily localized in lipid raft fractions containing caveolin-1 in hASCs. Consistent with this, the ∼90 kDa mature form of ADAM12 has been reported to predominantly localize at the cell surface, whereas the ∼120 kDa full-length form of ADAM12 exists in the trans-Golgi network [21]. Shedding activities of several ADAM isoforms, including ADAM10, ADAM17 and ADAM19, have been reported to take place in lipid rafts [46]–[48]. Despite the lack of direct evidence for the existence of ADAM12 in caveolin-positive lipid rafts, there is a report suggesting the co-localization of ADAM12 and caveolin-1 in a specific membrane domain [49]. Caveolin-1 has been reported to exist in the invadopodia of human breast cancer cells, and lipid rafts and caveolin-1 reportedly play a key role in invadopodia formation and protease actions within invadopodia [50]. Moreover, ADAM12 interacts with c-Src in invadopodia, which are dynamic actin structures located at the cell surface that degrade extracellular matrix and act as signal transduction sites, and ADAM12-mediated ectodomain shedding of epidermal growth factor receptor ligands occurs within invadopodia [49]. ADADM12-L has been reported to redistribute from perinuclear areas to actin-rich c-Src-positive structures at the cell periphery [51]. ADAM12 has been reported to localize to both the plasma membrane and endoplasmic reticulum, and the transport of ADAM12 to the plasma membrane depends on the proteolytic processing of the protein [21]. Treatment with phorbol esters induced ADAM12 translocation to the cell surface *via* mechanisms involving the catalytic activity of protein kinase Cε [52]. These results suggest that protein levels of ADAM12 in caveolin-1-containing membrane domains, *i.e.*, lipid rafts, can be regulated by extracellular stimuli, including TGF-β1.

TGF-β has been reported to up-regulate ADAM12 expression in hepatic stellate cells [42]. In addition, ADAM12 interacts with TGF-β type II receptor and inhibits degradation of TGF-β type II receptor by facilitating the trafficking of TGF-β type II receptor to the early endosome and preventing assembly of the TGF-β type II receptor and Smad7 complex. Therefore, ADAM12 stimulates activation of TGF-β signaling, including the phosphorylation of Smad2 and transcriptional activation [24]. In the present study, we demonstrated that silencing of ADAM12 expression using ADAM12-specific shRNA or siRNA abrogated TGF-β1-stimulated α-SMA expression and Smad2 phosphorylation. Taken together, these results suggest that TGF-β1 stimulates expression of ADAM12, which is involved in TGF-β1-mediated Smad2 phosphorylation and expression of SMC markers by regulating trafficking of the TGF-β receptor, and that ADAM12 plays a pivotal role in TGF-β1-stimulated differentiation of hASCs into SMCs.

## Supporting Information

Figure S1 **Staining of membrane cholesterol using filipin in MβCD-treated hASCs.** hASCs were treated with 5 mM MβCD for the indicated time periods, and stained with 50 µg/mL filipin for 30 min at room temperature. Fluorescence images were taken using a cooled CCD camera mounted on a Leica DM IRB inverted fluorescence microscope (Leica, Solms, Germany).
(JPG)

Figure S2 **Representative mass spectra of an LC-MS/MS feature identified as ADAM12 peptide TPGQWETGLR.**
(JPG)

Table S1 **Complete list of lipid raft-associated proteins of hASCs in the absence or presence of TGF-β1.**
(PDF)

Table S2 **Subcellular localization and molecular function of identified proteins.**
(PDF)

Table S3 **Functional annotation of up-regulated proteins by TGF-β1.**
(PDF)

Table S4 **Comparison of lipid raft proteome of hASCs with a database for lipid raft porteins (Zhang, T. et al. (2010) J. Mol. Biol., 402: 761–773).**
(PDF)
