# Supplementary figures and images for: Proteomic Identification of ADAM12 as a Regulator for TGF-β1-Induced Differentiation of Human Mesenchymal Stem Cells to Smooth Muscle Cells
Source: PLoS One. 2012 Jul 13;7(7):e40820. doi: 10.1371/journal.pone.0040820 (PMC3396647; doi:10.1371/journal.pone.0040820)

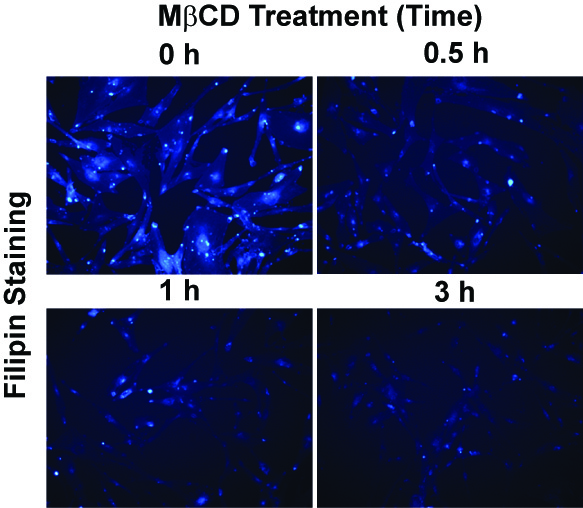

Supplement: Figure S1 — Staining of membrane cholesterol using filipin in MβCD-treated hASCs. hASCs were treated with 5 mM MβCD for the indicated time periods, and stained with 50 µg/mL filipin for 30 min at room temperature. Fluorescence images were taken using a cooled CCD camera mounted on a Leica DM IRB inverted fluorescence microscope (Leica, Solms, Germany). (JPG) [file pone.0040820.s001.jpg]

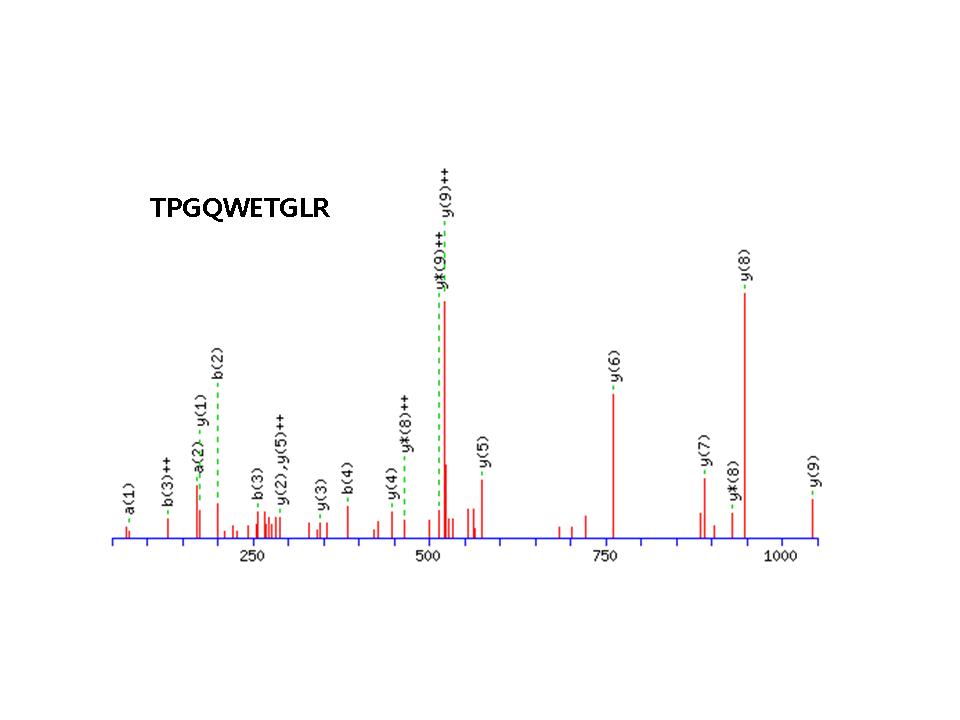

Supplement: Figure S2 — Representative mass spectra of an LC-MS/MS feature identified as ADAM12 peptide TPGQWETGLR. (JPG) [file pone.0040820.s002.jpg]
