# Supplementary material for: Proteomic Identification of ADAM12 as a Regulator for TGF-β1-Induced Differentiation of Human Mesenchymal Stem Cells to Smooth Muscle Cells
Source: PLoS One. 2012 Jul 13;7(7):e40820. doi: 10.1371/journal.pone.0040820 (PMC3396647; doi:10.1371/journal.pone.0040820)
Supplement: Table S1 — Complete list of lipid raft-associated proteins of hASCs in the absence or presence of TGF-β1. (PDF) [file pone.0040820.s003.pdf]

Table S1: Complete list of lipid raft-associated proteins of hASCs in the absence or presence of TGF-β1

Uniprot ID	Acc	Gene	Protein	spec#	TII_{CON}	$TII_{TGF\text{-}\beta 1}$	Log2Ratio
ADA12_HUMAN	O43184	ADAM12	Disintegrin and metalloproteinase domain-containing protein 12	6	0.1855	13.9495	6.233
PLOD2_HUMAN	O00469	PLOD2	Procollagen-lysine,2-oxoglutarate 5-dioxygenase 2	4	0.1855	9.6333	5.699
PVR_HUMAN	P15151	PVR	Poliovirus receptor	3	0.1855	8.5719	5.530
ALG2_HUMAN	Q9H553	ALG2	Alpha-1,3-mannosyltransferase ALG2	2	0.1855	6.7929	5.195
ACOD_HUMAN	O00767	SCD	Acyl-CoA desaturase	9	0.1855	6.6588	5.166
SATT_HUMAN	P43007	SLC1A4	Neutral amino acid transporter A	4	0.1855	6.6378	5.161
HYOU1_HUMAN	Q9Y4L1	HYOU1	Hypoxia up-regulated protein 1	3	0.1855	6.1864	5.060
FADS2_HUMAN	O95864	FADS2	Fatty acid desaturase 2	14	0.7811	24.6108	4.978
ATP5I_HUMAN	P56385	ATP5I	ATP synthase subunit e, mitochondrial	3	0.1855	5.8146	4.970
IDHP_HUMAN	P48735	IDH2	Isocitrate dehydrogenase [NADP], mitochondrial	5	0.1855	5.5572	4.905
P4HA2_HUMAN	O15460	P4HA2	Prolyl 4-hydroxylase subunit alpha-2	5	0.1855	5.5224	4.896
RS15_HUMAN	P62841	RPS15	40S ribosomal protein S15	2	0.1855	5.1227	4.787
LDLR_HUMAN	P01130	LDLR	Low-density lipoprotein receptor	8	0.1855	4.9291	4.732
B4DXK4_HUMAN	B4DXK4	KRT72	cDNA FLJ50908	2	0.1855	4.5899	4.629
RS15A_HUMAN	P62244	RPS15A	40S ribosomal protein S15a	3	0.1855	4.4844	4.595
PRKDC_HUMAN	P78527	PRKDC	DNA-dependent protein kinase catalytic subunit	5	0.1855	4.4452	4.583
PGH1_HUMAN	P23219	PTGS1	Prostaglandin G/H synthase 1	2	0.1855	4.3937	4.566
FND3B_HUMAN	Q53EP0	FNDC3B	Fibronectin type III domain-containing protein 3B	12	0.5107	11.3016	4.468
PKD2_HUMAN	Q13563	PKD2	Polycystin-2	2	0.1855	4.0117	4.435
SEM7A_HUMAN	O75326	SEMA7A	Semaphorin-7A	13	1.3005	25.5419	4.296
BGH3_HUMAN	Q15582	TGFBI	Transforming growth factor-beta-induced protein ig-h3	5	0.1855	3.5692	4.266
SGPL1_HUMAN	O95470	SGPL1	Sphingosine-1-phosphate lyase 1	4	0.1855	3.4418	4.214
S27A4_HUMAN	Q6P1M0	SLC27A4	Long-chain fatty acid transport protein 4	2	0.1855	3.1592	4.090
Q8TA92_HUMAN	Q8TA92	AFG3	Similar to AFG3 ATPase family gene 3-like 2 (Yeast) (Fragment)	5	0.1855	3.0358	4.033
RL29_HUMAN	P47914	RPL29	60S ribosomal protein L29	3	0.1855	2.7682	3.899
ANKL2_HUMAN	Q86XL3	ANKLE2	Ankyrin repeat and LEM domain-containing protein 2	2	0.1855	2.5974	3.808
PVRL2_HUMAN	Q92692	PVRL2	Poliovirus receptor-related protein 2	2	0.1855	2.4696	3.735
TMX1_HUMAN	Q9H3N1	TMX1	Thioredoxin-related transmembrane protein 1	5	0.643	8.4734	3.720
FKBP9_HUMAN	O95302	FKBP9	FK506-binding protein 9	2	0.1855	2.4011	3.694
COMP_HUMAN	P49747	COMP	Cartilage oligomeric matrix protein	2	0.1855	2.3775	3.680
RS27L_HUMAN	Q71UM5	RPS27L	40S ribosomal protein S27-like protein	2	0.1855	2.3102	3.639
GT251_HUMAN	Q8NBJ5	GLT25D1	Procollagen galactosyltransferase 1	3	0.1855	2.2885	3.625
MAN1_HUMAN	Q9Y2U8	LEMD3	Inner nuclear membrane protein Man1	6	0.3728	4.57	3.616
Q9UNR6_HUMAN	Q9UNR6	ERG1	Squalene epoxidase	7	0.4062	4.9386	3.604
Q495Q6_HUMAN	Q495Q6	LRRC15	HCG2043616	26	2.4062	29.2382	3.603
SDC1_HUMAN	P18827	SDC1	Syndecan-1	4	0.1855	2.217	3.579

LAMB1_HUMAN	P07942	LAMB1	Laminin subunit beta-1	3	0.1855	2.1987	3.567
ST1C3_HUMAN	Q6IMI6	SULT1C3	Sulfotransferase 1C3	3	3.1622	36.1998	3.517
TENC1_HUMAN	Q63HR2	TENC1	Tensin-like C1 domain-containing phosphatase	2	0.1855	2.098	3.500
PLOD1_HUMAN	Q02809	PLOD1	Procollagen-lysine,2-oxoglutarate 5-dioxygenase 1	12	0.787	8.5693	3.445
PTPRM_HUMAN	P28827	PTPRM	Receptor-type tyrosine-protein phosphatase mu	2	0.1855	2.0104	3.438
AP2M1_HUMAN	Q96CW1	AP2M1	AP-2 complex subunit mu	2	0.1855	2.0009	3.431
OSBL5_HUMAN	Q9H0X9	OSBPL5	Oxysterol-binding protein-related protein 5	3	0.1855	1.9084	3.363
TNR6_HUMAN	P25445	FAS	Tumor necrosis factor receptor superfamily member 6	2	0.1855	1.8982	3.355
ERP29_HUMAN	P30040	ERP29	Endoplasmic reticulum protein ERp29	3	0.1855	1.8221	3.296
FZD7_HUMAN	O75084	FZD7	Frizzled-7	2	0.1855	1.8167	3.292
HNRPC_HUMAN	P07910	HNRNPC	Heterogeneous nuclear ribonucleoproteins C1/C2	3	0.1855	1.8139	3.290
GPI8_HUMAN	Q92643	PIGK	GPI-anchor transamidase	7	0.6422	6.1883	3.268
MA1B1_HUMAN	Q9UKM7	MAN1B1	ER mannosyl-oligosaccharide 1,2-alpha-mannosidase	4	0.3129	2.9827	3.253
GOSR2_HUMAN	O14653	GOSR2	Golgi SNAP receptor complex member 2	7	0.8192	7.5071	3.196
VPS45_HUMAN	Q9NRW7	VPS45	Vacuolar protein sorting-associated protein 45	2	0.1855	1.6679	3.169
VPP1_HUMAN	Q93050	ATP6V0A1	V-type proton ATPase 116 kDa subunit a isoform 1	4	0.304	2.6715	3.136
MUC18_HUMAN	P43121	MCAM	Cell surface glycoprotein MUC18	8	0.9858	8.5858	3.123
SRPR_HUMAN	P08240	SRPR	Signal recognition particle receptor subunit alpha	26	4.8553	41.9714	3.112
OSBL8_HUMAN	Q9BZF1	OSBPL8	Oxysterol-binding protein-related protein 8	14	1.5145	12.9657	3.098
SPRC_HUMAN	P09486	SPARC	SPARC	7	1.0454	8.9208	3.093
MIA3_HUMAN	Q5JRA6	MIA3	Melanoma inhibitory activity protein 3	6	0.464	3.9297	3.082
B2CI53_HUMAN	B2CI53	SLC4A7	Solute carrier family 4 sodium bicarbonate cotransporter member 7	2	0.1855	1.5622	3.074
COCA1_HUMAN	Q99715	COL12A1	Collagen alpha-1(XII) chain	22	1.773	14.8637	3.068
IMPA3_HUMAN	Q9NX62	IMPAD1	Inositol monophosphatase 3	7	0.856	6.8884	3.008
FAT1_HUMAN	Q14517	FAT1	Protocadherin Fat 1	2	0.1855	1.4359	2.952
AL1B1_HUMAN	P30837	ALDH1B1	Aldehyde dehydrogenase X, mitochondrial	2	0.1855	1.404	2.920
HNRPM_HUMAN	P52272	HNRNPM	Heterogeneous nuclear ribonucleoprotein M	5	0.35	2.5252	2.851
BET1L_HUMAN	Q9NYM9	BET1L	BET1-like protein	2	0.1855	1.3061	2.816
FINC_HUMAN	P02751	FN1	Fibronectin	105	25.6875	175.903	2.776
VIGLN_HUMAN	Q00341	HDLBP	Vigilin	3	0.2941	1.9939	2.761
DERL1_HUMAN	Q9BUN8	DERL1	Derlin-1	2	0.1855	1.2446	2.746
USMG5_HUMAN	Q96IX5	USMG5	Up-regulated during skeletal muscle growth protein 5	2	0.1855	1.2269	2.726
PLCE1_HUMAN	Q9P212	PLCE1	1-phosphatidylinositol-4,5-bisphosphate phosphodiesterase epsilon-1	2	0.1855	1.2269	2.726
SCAM3_HUMAN	O14828	SCAMP3	Secretory carrier-associated membrane protein 3	2	0.1855	1.1882	2.679
F177A_HUMAN	Q8N128	FAM177A1	Protein FAM177A1	2	0.1855	1.186	2.677
TIM50_HUMAN	Q3ZCQ8	TIMM50	Mitochondrial import inner membrane translocase subunit TIM50	4	0.4617	2.8539	2.628
AP2B1_HUMAN	P63010	AP2B1	AP-2 complex subunit beta	5	0.6424	3.9032	2.603
CALU_HUMAN	O43852	CALU	Calumenin	5	0.9351	5.6507	2.595
F134C_HUMAN	Q86VR2	FAM134C	Protein FAM134C	2	0.1855	1.1128	2.585

TSP1_HUMAN	P07996	THBS1	Thrombospondin-1	64	13.973	82.7218	2.566
IF4A1_HUMAN	P60842	EIF4A1	Eukaryotic initiation factor 4A-I	13	1.1069	6.3766	2.526
Q9BW34_HUMAN	Q9BW34	EEF1D	EEF1D protein (Fragment)	4	0.3969	2.2792	2.522
UBXN4_HUMAN	Q92575	UBXN4	UBX domain-containing protein 4	3	0.581	3.2977	2.505
CPT1A_HUMAN	P50416	CPT1A	Carnitine O-palmitoyltransferase 1, liver isoform	3	0.3428	1.927	2.491
S38AA_HUMAN	Q9HBR0	SLC38A10	Putative sodium-coupled neutral amino acid transporter 10	3	0.4059	2.2704	2.484
RL36_HUMAN	Q9Y3U8	RPL36	60S ribosomal protein L36	5	0.7578	4.2002	2.471
MBOA7_HUMAN	Q96N66	MBOAT7	Lysophospholipid acyltransferase 7	6	0.6583	3.6304	2.463
RCN3_HUMAN	Q96D15	RCN3	Reticulocalbin-3	9	1.8816	10.3637	2.462
STX18_HUMAN	Q9P2W9	STX18	Syntaxin-18	5	0.4868	2.6604	2.450
CTNB1_HUMAN	P35222	CTNNB1	Catenin beta-1	49	7.4006	40.3724	2.448
P4HA1_HUMAN	P13674	P4HA1	Prolyl 4-hydroxylase subunit alpha-1	2	0.1855	1.0043	2.437
EGLN_HUMAN	P17813	ENG	Endoglin	17	6.4157	34.3979	2.423
A0AV88_HUMAN	A0AV88	ADAM10	ADAM10 protein	3	1.3939	7.2445	2.378
DYL1_HUMAN	P63167	DYNLL1	Dynein light chain 1, cytoplasmic	3	0.8974	4.5423	2.340
K220L_HUMAN	A8MRT5	KIAA0220L	Putative NPIP-like protein KIAA0220-like	2	0.1855	0.9092	2.293
CHSTE_HUMAN	Q8NCH0	CHST14	Carbohydrate sulfotransferase 14	2	0.1855	0.9059	2.288
PTK7_HUMAN	Q13308	PTK7	Tyrosine-protein kinase-like 7	34	8.775	41.6076	2.245
FIS1_HUMAN	Q9Y3D6	FIS1	Mitochondrial fission 1 protein	2	0.357	1.6847	2.238
4F2_HUMAN	P08195	SLC3A2	4F2 cell-surface antigen heavy chain	46	12.2123	56.8204	2.218
SSRB_HUMAN	P43308	SSR2	Translocon-associated protein subunit beta	27	15.3454	70.2513	2.195
RB22A_HUMAN	Q9UL26	RAB22A	Ras-related protein Rab-22A	4	0.4286	1.946	2.183
KDIS_HUMAN	Q9ULH0	KIDINS220	Kinase D-interacting substrate of 220 kDa	2	0.1855	0.8412	2.181
STX2_HUMAN	P32856	STX2	Syntaxin-2	4	0.8499	3.8386	2.175
CLPT1_HUMAN	O96005	CLPTM1	Cleft lip and palate transmembrane protein 1	4	1.7087	7.6376	2.160
UGPA_HUMAN	Q16851	UGP2	UTP--glucose-1-phosphate uridylyltransferase	2	0.3125	1.3937	2.157
K0776_HUMAN	O94874	KIAA0776	UPF0555 protein KIAA0776	16	3.6915	16.3526	2.147
SHPS1_HUMAN	P78324	SIRPA	Tyrosine-protein phosphatase non-receptor type substrate 1	6	1.6147	7.06	2.128
RL19_HUMAN	P84098	RPL19	60S ribosomal protein L19	2	0.1958	0.849	2.116
S12A4_HUMAN	Q9UP95	SLC12A4	Solute carrier family 12 member 4	11	2.5786	11.1213	2.109
SC65_HUMAN	Q92791	SC65	Synaptonemal complex protein SC65	2	0.1855	0.7883	2.087
PXDN_HUMAN	Q92626	PXDN	Peroxidasin homolog	2	0.1855	0.7686	2.051
K0090_HUMAN	Q8N766	KIAA0090	Uncharacterized protein KIAA0090	12	1.6627	6.8166	2.036
APOL2_HUMAN	Q9BQE5	APOL2	Apolipoprotein L2	3	1.1181	4.5357	2.020
TEN3_HUMAN	Q9P273	ODZ3	Teneurin-3	2	0.1855	0.7495	2.015
SEC63_HUMAN	Q9UGP8	SEC63	Translocation protein SEC63 homolog	17	4.9918	20.0672	2.007
CCD47_HUMAN	Q96A33	CCDC47	Coiled-coil domain-containing protein 47	11	2.0771	8.2122	1.983
RL27A_HUMAN	P46776	RPL27A	60S ribosomal protein L27a	11	4.295	16.8147	1.969
CG059_HUMAN	Q0VGL1	C7orf59	UPF0539 protein C7orf59	4	0.4697	1.831	1.963

RAB31_HUMAN	Q13636	RAB31	Ras-related protein Rab-31	6	0.6849	2.6691	1.962
GOSR1_HUMAN	O95249	GOSR1	Golgi SNAP receptor complex member 1	6	1.0277	3.813	1.892
LIPB1_HUMAN	Q86W92	PPFIBP1	Liprin-beta-1	2	0.1855	0.6877	1.890
DERL2_HUMAN	Q9GZP9	DERL2	Derlin-2	3	3.1805	11.7898	1.890
AP2A2_HUMAN	O94973	AP2A2	AP-2 complex subunit alpha-2	4	0.7054	2.6006	1.882
DYSF_HUMAN	O75923	DYSF	Dysferlin	3	0.5572	2.0488	1.879
GALT2_HUMAN	Q10471	GALNT2	Polypeptide N-acetylgalactosaminyltransferase 2	13	2.5361	9.3206	1.878
TPM1_HUMAN	P09493	TPM1	Tropomyosin alpha-1 chain	48	23.8542	86.9347	1.866
ITA1_HUMAN	P56199	ITGA1	Integrin alpha-1	16	3.1978	11.5866	1.857
MFGM_HUMAN	Q08431	MFGE8	Lactadherin	8	2.0163	7.2519	1.847
ACSL3_HUMAN	O95573	ACSL3	Long-chain-fatty-acid--CoA ligase 3	23	7.3545	26.4125	1.845
AAAT_HUMAN	Q15758	SLC1A5	Neutral amino acid transporter B(0)	23	34.7876	124.921	1.844
PICAL_HUMAN	Q13492	PICALM	Phosphatidylinositol-binding clathrin assembly protein	8	0.8668	3.0894	1.834
BT2A1_HUMAN	Q7KYR7	BTN2A1	Butyrophilin subfamily 2 member A1	3	0.4541	1.6181	1.833
NCPR_HUMAN	P16435	POR	NADPH--cytochrome P450 reductase	20	3.3219	11.6847	1.815
YIPF5_HUMAN	Q969M3	YIPF5	Protein YIPF5	5	1.141	3.8652	1.760
LIMA1_HUMAN	Q9UHB6	LIMA1	LIM domain and actin-binding protein 1	4	1.0039	3.3964	1.758
AP2A1_HUMAN	O95782	AP2A1	AP-2 complex subunit alpha-1	10	2.9299	9.7505	1.735
INF2_HUMAN	Q27J81	INF2	Inverted formin-2	2	0.2253	0.7463	1.728
MICA2_HUMAN	O94851	MICAL2	Protein MICAL-2	4	0.8539	2.8275	1.727
RDH11_HUMAN	Q8TC12	RDH11	Retinol dehydrogenase 11	13	3.4344	11.3568	1.725
GBG5_HUMAN	P63218	GNG5	Guanine nucleotide-binding protein G(I)/G(S)/G(O) subunit gamma-5	2	0.1855	0.6102	1.718
B2MG_HUMAN	P61769	B2M	Beta-2-microglobulin	5	1.0232	3.3286	1.702
SAR1B_HUMAN	Q9Y6B6	SAR1B	GTP-binding protein SAR1b	5	0.4562	1.4829	1.701
LGAT1_HUMAN	Q92604	LPGAT1	Acyl-CoA:lysophosphatidylglycerol acyltransferase 1	3	0.6333	2.0578	1.700
FA38A_HUMAN	Q92508	FAM38A	Protein FAM38A	3	0.3942	1.275	1.693
PI16_HUMAN	Q6UXB8	PI16	Peptidase inhibitor 16	43	32.105	102.577	1.676
MA2A1_HUMAN	Q16706	MAN2A1	Alpha-mannosidase 2	3	0.3493	1.1108	1.669
NCLN_HUMAN	Q969V3	NCLN	Nicalin	25	6.0042	18.9827	1.661
IF2A_HUMAN	P05198	EIF2S1	Eukaryotic translation initiation factor 2 subunit 1	2	0.2404	0.7573	1.655
SC61B_HUMAN	P60468	SEC61B	Protein transport protein Sec61 subunit beta	8	4.1983	13.1403	1.646
DHCR7_HUMAN	Q9UBM7	DHCR7	7-dehydrocholesterol reductase	10	2.6024	8.0745	1.634
KTN1_HUMAN	Q86UP2	KTN1	Kinectin	14	2.6	7.9872	1.619
VANG1_HUMAN	Q8TAA9	VANGL1	Vang-like protein 1	2	0.4738	1.4391	1.603
RRBP1_HUMAN	Q9P2E9	RRBP1	Ribosome-binding protein 1	83	50.3077	152.102	1.596
CD99_HUMAN	P14209	CD99	CD99 antigen	4	4.4849	13.4839	1.588
TM9S4_HUMAN	Q92544	TM9SF4	Transmembrane 9 superfamily member 4	3	0.9904	2.9398	1.570
GL8D1_HUMAN	Q68CQ7	GLT8D1	Glycosyltransferase 8 domain-containing protein 1	10	3.4363	10.1462	1.562
SPTC1_HUMAN	O15269	SPTLC1	Serine palmitoyltransferase 1	2	0.2321	0.6853	1.562

B2R701_HUMAN	B2R701	P116	cDNA, FLJ93202, Homo sapiens protease inhibitor 16 (PI16), mRNA	31	25.4107	74.5373	1.553
FAF2_HUMAN	Q96CS3	FAF2	FAS-associated factor 2	14	4.4947	13.1773	1.552
MPRI_HUMAN	P11717	IGF2R	Cation-independent mannose-6-phosphate receptor	4	0.7235	2.1108	1.545
ITA11_HUMAN	Q9UKX5	ITGA11	Integrin alpha-11	53	23.2606	67.6814	1.541
FDFT_HUMAN	P37268	FDFT1	Squalene synthetase	8	1.1756	3.3512	1.511
PDIA4_HUMAN	P13667	PDIA4	Protein disulfide-isomerase A4	20	7.6305	21.4292	1.490
CO1A2_HUMAN	P08123	COL1A2	Collagen alpha-2(I) chain	3	0.5448	1.5256	1.486
ITFG3_HUMAN	Q9H0X4	ITFG3	Protein ITFG3	6	2.7432	7.5321	1.457
TM165_HUMAN	Q9HC07	TMEM165	Transmembrane protein 165	9	6.6201	18.0786	1.449
SSRA_HUMAN	P43307	SSR1	Translocon-associated protein subunit alpha	34	21.072	57.4328	1.447
A7XZE4_HUMAN	A7XZE4	TPM2b	Beta tropomyosin isoform	55	30.2459	81.1828	1.424
ADT1_HUMAN	P12235	SLC25A4	ADP/ATP translocase 1	36	30.5199	81.6836	1.420
CTND1_HUMAN	O60716	CTNND1	Catenin delta-1	52	21.966	58.5406	1.414
PTH2_HUMAN	Q9Y3E5	PTRH2	Peptidyl-tRNA hydrolase 2, mitochondrial	7	2.1758	5.7331	1.398
CO024_HUMAN	Q9NPA0	C15orf24	UPF0480 protein C15orf24	10	2.2844	6.0171	1.397
AT1B1_HUMAN	P05026	ATP1B1	Sodium/potassium-transporting ATPase subunit beta-1	5	1.723	4.5377	1.397
EPHB3_HUMAN	P54753	EPHB3	Ephrin type-B receptor 3	6	1.6214	4.2569	1.393
NUCB2_HUMAN	P80303	NUCB2	Nucleobindin-2	2	0.1855	0.4844	1.385
RL32_HUMAN	P62910	RPL32	60S ribosomal protein L32	4	2.1754	5.6735	1.383
ERP44_HUMAN	Q9BS26	ERP44	Endoplasmic reticulum resident protein ERp44	11	3.1167	8.097	1.377
CSPG4_HUMAN	Q6UVK1	CSPG4	Chondroitin sulfate proteoglycan 4	15	5.0992	13.2128	1.374
STX12_HUMAN	Q86Y82	STX12	Syntaxin-12	18	8.595	22.139	1.365
AT2A2_HUMAN	P16615	ATP2A2	Sarcoplasmic/endoplasmic reticulum calcium ATPase 2	62	20.1724	51.8686	1.362
COMT_HUMAN	P21964	COMT	Catechol O-methyltransferase	22	5.0429	12.8612	1.351
SRPRB_HUMAN	Q9Y5M8	SRPRB	Signal recognition particle receptor subunit beta	40	14.0123	35.5044	1.341
CTL2_HUMAN	Q8IWA5	SLC44A2	Choline transporter-like protein 2	3	1.3671	3.4543	1.337
NUCB1_HUMAN	Q02818	NUCB1	Nucleobindin-1	7	2.7481	6.9247	1.333
RENR_HUMAN	O75787	ATP6AP2	Renin receptor	5	3.3862	8.5124	1.330
Q5TCU3_HUMAN	Q5TCU3	TPM2	Tropomyosin 2 (Beta)	53	30.2459	75.5579	1.321
NPM_HUMAN	P06748	NPM1	Nucleophosmin	2	0.1855	0.4625	1.318
SL9A1_HUMAN	P19634	SLC9A1	Sodium/hydrogen exchanger 1	12	4.9446	12.3052	1.315
LMF2_HUMAN	Q9BU23	LMF2	Lipase maturation factor 2	6	0.8064	2.0015	1.312
H2AV_HUMAN	Q71UI9	H2AFV	Histone H2A.V	10	5.7169	14.0935	1.302
NICA_HUMAN	Q92542	NCSTN	Nicastrin	16	4.6303	11.4016	1.300
H2AJ_HUMAN	Q9BTM1	H2AFJ	Histone H2A.J	15	10.024	24.6821	1.300
EPHA7_HUMAN	Q15375	EPHA7	Ephrin type-A receptor 7	3	0.5776	1.4217	1.299
RL27_HUMAN	P61353	RPL27	60S ribosomal protein L27	5	3.3393	8.2135	1.298
SURF4_HUMAN	O15260	SURF4	Surfeit locus protein 4	28	12.0837	29.7195	1.298
CADH2_HUMAN	P19022	CDH2	Cadherin-2	46	19.8046	48.6068	1.295

RAB21_HUMAN	Q9UL25	RAB21	Ras-related protein Rab-21	11	2.5814	6.2969	1.286
STX7_HUMAN	O15400	STX7	Syntaxin-7	12	6.8478	16.5048	1.269
P3H3_HUMAN	Q8IVL6	LEPREL2	Prolyl 3-hydroxylase 3	8	1.6552	3.9842	1.267
NOMO1_HUMAN	Q15155	NOMO1	Nodal modulator 1	24	10.0892	24.0046	1.250
RAB6A_HUMAN	P20340	RAB6A	Ras-related protein Rab-6A	32	23.4748	55.233	1.234
H2BFS_HUMAN	P57053	H2BFS	Histone H2B type F-S	11	2.9065	6.8313	1.233
SSRD_HUMAN	P51571	SSR4	Translocon-associated protein subunit delta	43	28.6149	66.5188	1.217
H2A2A_HUMAN	Q6FI13	HIST2H2AA3	Histone H2A type 2-A	17	10.024	23.2563	1.214
ERG25_HUMAN	Q15800	SC4MOL	C-4 methylsterol oxidase	3	0.6207	1.4391	1.213
LPLC3_HUMAN	P59826	LPLUNC3	Long palate, lung and nasal epithelium carcinoma-associated protein 3	4	1.9892	4.5862	1.205
RL12_HUMAN	P30050	RPL12	60S ribosomal protein L12	10	2.1227	4.8596	1.195
TOR1A_HUMAN	O14656	TOR1A	Torsin-1A	5	0.7367	1.6865	1.195
RL15_HUMAN	P61313	RPL15	60S ribosomal protein L15	11	4.4359	10.1038	1.188
FKB10_HUMAN	Q96AY3	FKBP10	FK506-binding protein 10	26	10.2429	23.2559	1.183
PARVA_HUMAN	Q9NVD7	PARVA	Alpha-parvin	2	0.4845	1.0971	1.179
TAGL_HUMAN	Q01995	TAGLN	Transgelin	39	16.8683	38.1941	1.179
HBXIP_HUMAN	O43504	HBXIP	Hepatitis B virus X-interacting protein	4	0.9596	2.172	1.179
Q5HYB6_HUMAN	Q5HYB6	TPM3	Putative uncharacterized protein DKFZp686J1372	31	22.9476	51.6794	1.171
NCEH1_HUMAN	Q6PIU2	NCEH1	Neutral cholesterol ester hydrolase 1	9	5.0091	11.2528	1.168
TMEM2_HUMAN	Q9UHN6	TMEM2	Transmembrane protein 2	3	0.4421	0.9886	1.161
ADT2_HUMAN	P05141	SLC25A5	ADP/ATP translocase 2	51	42.1381	93.8743	1.156
RAN_HUMAN	P62826	RAN	GTP-binding nuclear protein Ran	2	0.4278	0.9483	1.148
SMC3_HUMAN	Q9UQE7	SMC3	Structural maintenance of chromosomes protein 3	2	1.0581	2.3375	1.143
STT3A_HUMAN	P46977	STT3A	Dolichyl-diphosphooligosaccharide--protein glycosyltransferase subunit STT3A	20	5.5175	12.1255	1.136
MOS_HUMAN	P00540	MOS	Proto-oncogene serine/threonine-protein kinase mos	2	0.872	1.9147	1.135
NDUS2_HUMAN	O75306	NDUFS2	NADH dehydrogenase [ubiquinone] iron-sulfur protein 2, mitochondrial	2	0.9817	2.1499	1.131
HM13_HUMAN	Q8TCT9	HM13	Minor histocompatibility antigen H13	3	0.7903	1.7307	1.131
RL8_HUMAN	P62917	RPL8	60S ribosomal protein L8	3	2.5949	5.6807	1.130
CCD19_HUMAN	Q9UL16	CCDC19	Coiled-coil domain-containing protein 19, mitochondrial	2	1.3711	2.9785	1.119
RL13_HUMAN	P26373	RPL13	60S ribosomal protein L13	17	7.3465	15.9326	1.117
LYRIC_HUMAN	Q86UE4	MTDH	Protein LYRIC	16	8.7058	18.6442	1.099
YIF1B_HUMAN	Q5BJH7	YIF1B	Protein YIF1B	6	2.3258	4.9669	1.095
RS13_HUMAN	P62277	RPS13	40S ribosomal protein S13	10	5.1301	10.8941	1.086
TBL2_HUMAN	Q9Y4P3	TBL2	Transducin beta-like protein 2	17	18.268	38.6802	1.082
MXRA7_HUMAN	P84157	MXRA7	Matrix-remodeling-associated protein 7	10	11.4954	24.0947	1.068
MOGS_HUMAN	Q13724	MOGS	Mannosyl-oligosaccharide glucosidase	53	37.2727	77.9125	1.064
ADT3_HUMAN	P12236	SLC25A6	ADP/ATP translocase 3	56	49.7438	103.602	1.058

RAC1_HUMAN	P63000	RAC1	Ras-related C3 botulinum toxin substrate 1	27	11.2982	23.4945	1.056
Q562Z4_HUMAN	Q562Z4	ACT	Actin-like protein (Fragment)	39	236.212	487.058	1.044
GBLP_HUMAN	P63244	GNB2L1	Guanine nucleotide-binding protein subunit beta-2-like 1	5	1.8307	3.7699	1.042
PGAM1_HUMAN	P18669	PGAM1	Phosphoglycerate mutase 1	4	2.6031	5.3559	1.041
GOLI4_HUMAN	O00461	GOLIM4	Golgi integral membrane protein 4	7	4.5266	9.2576	1.032
UTRO_HUMAN	P46939	UTRN	Utrophin	37	13.1893	26.9018	1.028
A5YM53_HUMAN	A5YM53	ITGAV	ITGAV protein	165	74.0798	151.004	1.027
DRS7B_HUMAN	Q6IAN0	DHRS7B	Dehydrogenase/reductase SDR family member 7B	11	2.3378	4.7602	1.026
CALR_HUMAN	P27797	CALR	Calreticulin	46	29.1637	59.2211	1.022
SSRG_HUMAN	Q9UNL2	SSR3	Translocon-associated protein subunit gamma	10	5.915	11.9574	1.015
CD47_HUMAN	Q08722	CD47	Leukocyte surface antigen CD47	2	0.6576	1.3206	1.006
RAP1A_HUMAN	P62834	RAP1A	Ras-related protein Rap-1A	41	28.8638	57.8317	1.003
CKAP4_HUMAN	Q07065	CKAP4	Cytoskeleton-associated protein 4	451	571.124	1144.18	1.002
SERPH_HUMAN	P50454	SERPINH1	Serpin H1	149	160.373	320.004	0.997
DMD_HUMAN	P11532	DMD	Dystrophin	2	0.1874	0.3724	0.991
CALX_HUMAN	P27824	CANX	Calnexin	211	207.027	411.351	0.991
ITB1_HUMAN	P05556	ITGB1	Integrin beta-1	162	149.513	296.146	0.986
TXTP_HUMAN	P53007	SLC25A1	Tricarboxylate transport protein, mitochondrial	13	4.2631	8.438	0.985
SAC1_HUMAN	Q9NTJ5	SACM1L	Phosphatidylinositide phosphatase SAC1	11	2.8899	5.7113	0.983
ARL1_HUMAN	P40616	ARL1	ADP-ribosylation factor-like protein 1	10	1.8674	3.6634	0.972
CXA1_HUMAN	P17302	GJA1	Gap junction alpha-1 protein	9	5.7535	11.2767	0.971
HNRPU_HUMAN	Q00839	HNRNPU	Heterogeneous nuclear ribonucleoprotein U	5	1.3749	2.6884	0.967
ELOV1_HUMAN	Q9BW60	ELOVL1	Elongation of very long chain fatty acids protein 1	15	7.4329	14.4758	0.962
COX5B_HUMAN	P10606	COX5B	Cytochrome c oxidase subunit 5B, mitochondrial	7	4.1453	8.0426	0.956
ERD22_HUMAN	P33947	KDELR2	ER lumen protein retaining receptor 2	6	1.3441	2.6048	0.955
LAMP1_HUMAN	P11279	LAMP1	Lysosome-associated membrane glycoprotein 1	26	8.08	15.6567	0.954
ENPL_HUMAN	P14625	HSP90B1	Endoplasmin	59	34.7089	67.1543	0.952
RSSA_HUMAN	P08865	RPSA	40S ribosomal protein SA	11	4.9155	9.4931	0.950
ARF4_HUMAN	P18085	ARF4	ADP-ribosylation factor 4	24	11.7686	22.6371	0.944
RAB8B_HUMAN	Q92930	RAB8B	Ras-related protein Rab-8B	19	15.9078	30.53	0.940
PIGS_HUMAN	Q96S52	PIGS	GPI transamidase component PIG-S	7	3.0077	5.7532	0.936
ZDHC5_HUMAN	Q9C0B5	ZDHHC5	Probable palmitoyltransferase ZDHHC5	2	0.7481	1.4275	0.932
RL18A_HUMAN	Q02543	RPL18A	60S ribosomal protein L18a	2	0.8471	1.6038	0.921
RAP1B_HUMAN	P61224	RAP1B	Ras-related protein Rap-1b	47	31.5538	59.659	0.919
CTNA1_HUMAN	P35221	CTNNA1	Catenin alpha-1	113	77.3858	146.064	0.916
Q58F09_HUMAN	Q58F09	GCS1	Glucosidase I	53	40.3409	76.1412	0.916
TM9S2_HUMAN	Q99805	TM9SF2	Transmembrane 9 superfamily member 2	12	3.476	6.5532	0.915
UN84B_HUMAN	Q9UH99	UNC84B	Protein unc-84 homolog B	28	19.3802	36.5128	0.914
Q3B7K3_HUMAN	Q3B7K3	IKIP	IKIP protein (Fragment)	47	16.9824	31.942	0.911

TMM33_HUMAN	P57088	TMEM33	Transmembrane protein 33	8	2.0886	3.9209	0.909
TOM22_HUMAN	Q9NS69	TOMM22	Mitochondrial import receptor subunit TOM22 homolog	8	5.1612	9.6298	0.900
GNAS1_HUMAN	Q5JWF2	GNAS	Guanine nucleotide-binding protein G(s) subunit alpha isoforms XLas	60	48.2756	90.0218	0.899
GSLG1_HUMAN	Q92896	GLG1	Golgi apparatus protein 1	36	13.0871	24.3733	0.897
AL2S4_HUMAN	Q96Q45	ALS2CR4	Amyotrophic lateral sclerosis 2 chromosomal region candidate gene 4 protein	3	0.3844	0.7151	0.896
TMED4_HUMAN	Q7Z7H5	TMED4	Transmembrane emp24 domain-containing protein 4	18	16.7539	31.1121	0.893
TXND5_HUMAN	Q8NBS9	TXNDC5	Thioredoxin domain-containing protein 5	20	6.6311	12.3017	0.892
TMM85_HUMAN	Q5J8M3	TMEM85	Transmembrane protein 85	4	1.8498	3.4248	0.889
CSRP1_HUMAN	P21291	CSRP1	Cysteine and glycine-rich protein 1	3	1.3627	2.5118	0.882
CD166_HUMAN	Q13740	ALCAM	CD166 antigen	132	112.558	207.045	0.879
PHB2_HUMAN	Q99623	PHB2	Prohibitin-2	60	46.7205	85.7006	0.875
TUSC3_HUMAN	Q13454	TUSC3	Tumor suppressor candidate 3	6	2.073	3.7714	0.863
ITB5_HUMAN	P18084	ITGB5	Integrin beta-5	40	14.9639	27.0385	0.854
RPN1_HUMAN	P04843	RPN1	Dolichyl-diphosphooligosaccharide--protein glycosyltransferase subunit 1	192	241.458	433.737	0.845
TMX3_HUMAN	Q96JJ7	TMX3	Protein disulfide-isomerase TMX3	19	6.189	11.1152	0.845
TOM70_HUMAN	O94826	TOMM70A	Mitochondrial import receptor subunit TOM70	11	6.3887	11.4645	0.844
RAB35_HUMAN	Q15286	RAB35	Ras-related protein Rab-35	19	16.354	29.3301	0.843
RAB14_HUMAN	P61106	RAB14	Ras-related protein Rab-14	53	26.9145	48.0873	0.837
TPM4_HUMAN	P67936	TPM4	Tropomyosin alpha-4 chain	49	38.9227	69.32	0.833
IKIP_HUMAN	Q70UQ0	IKIP	Inhibitor of nuclear factor kappa-B kinase-interacting protein	53	19.8797	35.3277	0.830
DREB_HUMAN	Q16643	DBN1	Drebrin	7	2.9906	5.2922	0.823
RAB2A_HUMAN	P61019	RAB2A	Ras-related protein Rab-2A	62	24.0626	42.5802	0.823
VKGC_HUMAN	P38435	GGCX	Vitamin K-dependent gamma-carboxylase	7	1.5694	2.7686	0.819
RS23_HUMAN	P62266	RPS23	40S ribosomal protein S23	6	1.9361	3.401	0.813
ALG5_HUMAN	Q9Y673	ALG5	Dolichyl-phosphate beta-glucosyltransferase	8	1.8869	3.3013	0.807
ADPGK_HUMAN	Q9BRR6	ADPGK	ADP-dependent glucokinase	10	3.8137	6.6413	0.800
MGST3_HUMAN	O14880	MGST3	Microsomal glutathione S-transferase 3	9	2.8488	4.958	0.799
EF1G_HUMAN	P26641	EEF1G	Elongation factor 1-gamma	9	2.9001	5.0059	0.788
AIFM1_HUMAN	O95831	AIFM1	Apoptosis-inducing factor 1, mitochondrial	3	1.4013	2.4175	0.787
ZW10_HUMAN	O43264	ZW10	Centromere/kinetochore protein zw10 homolog	5	0.874	1.5071	0.786
BST1_HUMAN	Q10588	BST1	ADP-ribosyl cyclase 2	19	7.4345	12.7752	0.781
CHP1_HUMAN	Q99653	CHP	Calcium-binding protein p22	7	2.3987	4.1168	0.779
GAPR1_HUMAN	Q9H4G4	GLIPR2	Golgi-associated plant pathogenesis-related protein 1	22	32.7458	55.8983	0.771
GOGA5_HUMAN	Q8TBA6	GOLGA5	Golgin subfamily A member 5	3	1.0394	1.767	0.766
GRP78_HUMAN	P11021	HSPA5	78 kDa glucose-regulated protein	167	181.961	308.407	0.761
PPAC2_HUMAN	Q8IY26	PPAPDC2	Presqualene diphosphate phosphatase	2	0.4863	0.823	0.759
ADA17_HUMAN	P78536	ADAM17	Disintegrin and metalloproteinase domain-containing protein 17	4	1.805	3.0525	0.758

RAB8A_HUMAN	P61006	RAB8A	Ras-related protein Rab-8A	22	18.0352	30.4736	0.757
MYOF_HUMAN	Q9NZM1	MYOF	Myoferlin	242	164.953	278.312	0.755
GALT1_HUMAN	Q10472	GALNT1	Polypeptide N-acetylgalactosaminyltransferase 1	11	5.9418	9.9942	0.750
EBP_HUMAN	Q15125	EBP	3-beta-hydroxysteroid-Delta(8),Delta(7)-isomerase	10	12.5444	21.0821	0.749
CTGE5_HUMAN	O15320	CTAGE5	Cutaneous T-cell lymphoma-associated antigen 5	7	3.0403	5.1065	0.748
COX41_HUMAN	P13073	COX4I1	Cytochrome c oxidase subunit 4 isoform 1, mitochondrial	17	15.1311	25.3766	0.746
LAMP2_HUMAN	P13473	LAMP2	Lysosome-associated membrane glycoprotein 2	17	7.5575	12.5478	0.731
RAB10_HUMAN	P61026	RAB10	Ras-related protein Rab-10	38	38.4627	63.8014	0.730
ENPP2_HUMAN	Q13822	ENPP2	Ectonucleotide pyrophosphatase/phosphodiesterase family member 2	2	1.4569	2.4155	0.729
NSDHL_HUMAN	Q15738	NSDHL	Sterol-4-alpha-carboxylate 3-dehydrogenase, decarboxylating	18	20.0109	32.7879	0.712
ATPK_HUMAN	P56134	ATP5J2	ATP synthase subunit f, mitochondrial	18	8.8562	14.4162	0.703
FLOT1_HUMAN	O75955	FLOT1	Flotillin-1	23	10.9445	17.7799	0.700
CLH1_HUMAN	Q00610	CLTC	Clathrin heavy chain 1	134	54.7223	88.8763	0.700
TFR1_HUMAN	P02786	TFRC	Transferrin receptor protein 1	17	4.7545	7.719	0.699
KIRR1_HUMAN	Q96J84	KIRREL	Kin of IRRE-like protein 1	7	4.5523	7.3796	0.697
EFTU_HUMAN	P49411	TUFM	Elongation factor Tu, mitochondrial	12	4.1121	6.6611	0.696
RB11B_HUMAN	Q15907	RAB11B	Ras-related protein Rab-11B	35	14.8348	24.0069	0.694
DNJC1_HUMAN	Q96KC8	DNAJC1	DnaJ homolog subfamily C member 1	7	3.085	4.9818	0.691
LRC59_HUMAN	Q96AG4	LRRC59	Leucine-rich repeat-containing protein 59	78	88.8601	143.302	0.689
TOM40_HUMAN	O96008	TOMM40	Mitochondrial import receptor subunit TOM40 homolog	5	3.0912	4.97	0.685
TM214_HUMAN	Q6NUQ4	TMEM214	Transmembrane protein 214	22	8.7471	14.0199	0.681
RRAS2_HUMAN	P62070	RRAS2	Ras-related protein R-Ras2	25	12.4988	20.0242	0.680
ESYT1_HUMAN	Q9BSJ8	ESYT1	Extended synaptotagmin-1	56	27.8689	44.6048	0.679
SCFD1_HUMAN	Q8WVM8	SCFD1	Sec1 family domain-containing protein 1	5	1.4218	2.267	0.673
CAND1_HUMAN	Q86VP6	CAND1	Cullin-associated NEDD8-dissociated protein 1	7	2.229	3.5517	0.672
MYO1C_HUMAN	O00159	MYO1C	Myosin-Ic	67	27.6255	43.9381	0.669
RB11A_HUMAN	P62491	RAB11A	Ras-related protein Rab-11A	36	15.1647	24.0328	0.664
PDIA1_HUMAN	P07237	P4HB	Protein disulfide-isomerase	97	87.9463	138.627	0.657
PIGT_HUMAN	Q969N2	PIGT	GPI transamidase component PIG-T	16	4.8668	7.6614	0.655
UCRI_HUMAN	P47985	UQCRFS1	Cytochrome b-c1 complex subunit Rieske, mitochondrial	2	1.3495	2.1238	0.654
UCRI_HUMAN	P47985	UQCRFS1	Cytochrome b-c1 complex subunit Rieske, mitochondrial	4	1.3495	2.1238	0.654
LETM1_HUMAN	O95202	LETM1	LETM1 and EF-hand domain-containing protein 1, mitochondrial	4	1.159	1.8184	0.650
A4D1N4_HUMAN	A4D1N4	CHCHD3	Coiled-coil-helix-coiled-coil-helix domain containing 3, isoform CRA_d	10	8.1405	12.7359	0.646
GNA13_HUMAN	Q14344	GNA13	Guanine nucleotide-binding protein subunit alpha-13	40	40.3129	62.8722	0.641
STML2_HUMAN	Q9UJZ1	STOML2	Stomatin-like protein 2	11	4.4683	6.9517	0.638
RAB1B_HUMAN	Q9H0U4	RAB1B	Ras-related protein Rab-1B	68	71.6582	111.108	0.633
RLA0_HUMAN	P05388	RPLP0	60S acidic ribosomal protein P0	7	4.1569	6.4216	0.627
C9JSZ5_HUMAN	C9JSZ5	DDRGK1	Putative uncharacterized protein DDRGK1	12	4.955	7.645	0.626
PDIA3_HUMAN	P30101	PDIA3	Protein disulfide-isomerase A3	96	114.28	175.569	0.619

ESYT2_HUMAN	A0FGR8	ESYT2	Extended synaptotagmin-2	12	5.4254	8.3324	0.619
Q5SV24_HUMAN	Q5SV24	ATAD3A	ATPase family, AAA domain containing 3A (Fragment)	40	17.3748	26.6819	0.619
B8ZZQ7_HUMAN	B8ZZQ7	IMMT	Putative uncharacterized protein IMMT	40	30.4799	46.7794	0.618
PROF1_HUMAN	P07737	PFN1	Profilin-1	19	13.8514	21.2228	0.616
SCMC1_HUMAN	Q6NUK1	SLC25A24	Calcium-binding mitochondrial carrier protein SCaMC-1	15	4.9436	7.5561	0.612
F176B_HUMAN	Q9NVM1	FAM176B	Protein FAM176B	2	1.2551	1.9163	0.611
SC22B_HUMAN	O75396	SEC22B	Vesicle-trafficking protein SEC22b	79	57.198	87.1009	0.607
DAD1_HUMAN	P61803	DAD1	Dolichyl-diphosphooligosaccharide--protein glycosyltransferase subunit DAD1	9	3.0343	4.5886	0.597
CK059_HUMAN	Q6IAA8	C11orf59	RhoA activator C11orf59	15	5.2846	7.9779	0.594
PALM_HUMAN	O75781	PALM	Paralemmin	3	1.8448	2.7759	0.589
RASH_HUMAN	P01112	HRAS	GTPase HRas	15	15.8696	23.8725	0.589
ERG7_HUMAN	P48449	LSS	Lanosterol synthase	10	3.3786	5.0737	0.587
TTC35_HUMAN	Q15006	TTC35	Tetratricopeptide repeat protein 35	2	0.9186	1.378	0.585
RS18_HUMAN	P62269	RPS18	40S ribosomal protein S18	15	5.5972	8.3959	0.585
DHC24_HUMAN	Q15392	DHCR24	24-dehydrocholesterol reductase	15	4.2674	6.3931	0.583
PCAT1_HUMAN	Q8NF37	LPCAT1	Lysophosphatidylcholine acyltransferase 1	3	0.6868	1.0286	0.583
DHB12_HUMAN	Q53GQ0	HSD17B12	Estradiol 17-beta-dehydrogenase 12	11	4.418	6.5906	0.577
RS17_HUMAN	P08708	RPS17	40S ribosomal protein S17	2	1.8111	2.6988	0.575
PRAF2_HUMAN	O60831	PRAF2	PRA1 family protein 2	12	15.388	22.9266	0.575
Q3B7A4_HUMAN	Q3B7A4	RPLP0	RPLP0 protein	9	4.7687	7.1035	0.575
TPBG_HUMAN	Q13641	TPBG	Trophoblast glycoprotein	11	3.9414	5.8637	0.573
MAGT1_HUMAN	Q9H0U3	MAGT1	Magnesium transporter protein 1	5	1.3246	1.9634	0.568
VAPB_HUMAN	O95292	VAPB	Vesicle-associated membrane protein-associated protein B/C	17	19.9233	29.4876	0.566
RAB7A_HUMAN	P51149	RAB7A	Ras-related protein Rab-7a	80	59.6583	88.1145	0.563
FKB11_HUMAN	Q9NYL4	FKBP11	FK506-binding protein 11	27	18.6827	27.5039	0.558
Q8TEP9_HUMAN	Q8TEP9	FLJ00144	FLJ00144 protein (Fragment)	64	58.8201	86.411	0.555
NSF_HUMAN	P46459	NSF	Vesicle-fusing ATPase	5	1.6058	2.3512	0.550
PDIA6_HUMAN	Q15084	PDIA6	Protein disulfide-isomerase A6	28	13.3992	19.5541	0.545
LRP1_HUMAN	Q07954	LRP1	Prolow-density lipoprotein receptor-related protein 1	160	114.803	166.385	0.535
CATB_HUMAN	P07858	CTSB	Cathepsin B	5	5.2866	7.661	0.535
BET1_HUMAN	O15155	BET1	BET1 homolog	2	1.3056	1.8865	0.531
TMED9_HUMAN	Q9BVK6	TMED9	Transmembrane emp24 domain-containing protein 9	21	11.4729	16.5618	0.530
PTN1_HUMAN	P18031	PTPN1	Tyrosine-protein phosphatase non-receptor type 1	6	2.8896	4.1603	0.526
PDCD6_HUMAN	O75340	PDCD6	Programmed cell death protein 6	5	0.9129	1.3099	0.521
RL3L_HUMAN	Q92901	RPL3L	60S ribosomal protein L3-like	2	2.1548	3.0829	0.517
PGRC2_HUMAN	O15173	PGRMC2	Membrane-associated progesterone receptor component 2	44	43.8483	62.4534	0.510
TX1B3_HUMAN	O14907	TAX1BP3	Tax1-binding protein 3	2	0.61	0.8669	0.507
RHOG_HUMAN	P84095	RHOG	Rho-related GTP-binding protein RhoG	10	14.6506	20.7762	0.504

1433B_HUMAN	P31946	YWHAB	14-3-3 protein beta/alpha	9	2.8756	4.0582	0.497
TECR_HUMAN	Q9NZ01	TECR	Trans-2,3-enoyl-CoA reductase	16	11.8856	16.7247	0.493
MFS10_HUMAN	Q14728	MFSD10	Major facilitator superfamily domain-containing protein 10	5	1.345	1.8893	0.490
SC11A_HUMAN	P67812	SEC11A	Signal peptidase complex catalytic subunit SEC11A	17	4.8806	6.8555	0.490
ATLA3_HUMAN	Q6DD88	ATL3	Atlastin-3	94	80.2557	112.53	0.488
C7FDR3_HUMAN	C7FDR3	HLA-B	MHC class I antigen (Fragment)	25	15.657	21.8637	0.482
MPKS1_HUMAN	Q9UHA4	MAPKSP1	Mitogen-activated protein kinase scaffold protein 1	2	0.4812	0.6717	0.481
USO1_HUMAN	O60763	USO1	General vesicular transport factor p115	6	1.7829	2.4796	0.476
SYFA_HUMAN	Q9Y285	FARSA	Phenylalanyl-tRNA synthetase alpha chain	2	0.3245	0.4502	0.472
K1C18_HUMAN	P05783	KRT18	Keratin, type I cytoskeletal 18	11	6.0087	8.3055	0.467
S61A1_HUMAN	P61619	SEC61A1	Protein transport protein Sec61 subunit alpha isoform 1	4	1.4667	2.02	0.462
CNN2_HUMAN	Q99439	CNN2	Calponin-2	8	3.4702	4.7697	0.459
RETST_HUMAN	Q6NUM9	RETSAT	All-trans-retinol 13,14-reductase	3	0.7746	1.0596	0.452
CD63_HUMAN	P08962	CD63	CD63 antigen	4	4.8565	6.6406	0.451
RL23A_HUMAN	P62750	RPL23A	60S ribosomal protein L23a	2	1.3537	1.8453	0.447
PTAD1_HUMAN	Q9P035	PTPLAD1	Protein tyrosine phosphatase-like protein PTPLAD1	15	7.0618	9.597	0.443
RS3_HUMAN	P23396	RPS3	40S ribosomal protein S3	13	5.0108	6.7994	0.440
IMB1_HUMAN	Q14974	KPNB1	Importin subunit beta-1	15	4.2843	5.8058	0.438
IMMT_HUMAN	Q16891	IMMT	Mitochondrial inner membrane protein	51	43.5709	58.9918	0.437
QCR6_HUMAN	P07919	UQCRH	Cytochrome b-c1 complex subunit 6, mitochondrial	4	0.7473	1.0092	0.433
CO6A3_HUMAN	P12111	COL6A3	Collagen alpha-3(VI) chain	7	1.6336	2.204	0.432
NDUV1_HUMAN	P49821	NDUFV1	NADH dehydrogenase [ubiquinone] flavoprotein 1, mitochondrial	3	0.7529	1.0152	0.431
GNAI3_HUMAN	P08754	GNAI3	Guanine nucleotide-binding protein G(k) subunit alpha	77	64.163	86.4467	0.430
PRDX6_HUMAN	P30041	PRDX6	Peroxiredoxin-6	6	2.0989	2.8266	0.429
GANAB_HUMAN	Q14697	GANAB	Neutral alpha-glucosidase AB	57	36.2188	48.5786	0.424
RRAS_HUMAN	P10301	RRAS	Ras-related protein R-Ras	27	15.0165	20.0312	0.416
CA2D1_HUMAN	P54289	CACNA2D1	Voltage-dependent calcium channel subunit alpha-2/delta-1	17	6.0878	8.1198	0.416
SPCS2_HUMAN	Q15005	SPCS2	Signal peptidase complex subunit 2	17	7.1922	9.5832	0.414
TMED7_HUMAN	Q9Y3B3	TMED7	Transmembrane emp24 domain-containing protein 7	31	18.3609	24.4494	0.413
HXK1_HUMAN	P19367	HK1	Hexokinase-1	19	10.2965	13.6783	0.410
DEST_HUMAN	P60981	DSTN	Destrin	4	1.5377	2.0334	0.403
ARF6_HUMAN	P62330	ARF6	ADP-ribosylation factor 6	2	0.5514	0.7247	0.394
Q53TP5_HUMAN	Q53TP5	FAP	Fibroblast activation protein, alpha, isoform CRA_a	135	97.4545	127.671	0.390
P3H1_HUMAN	Q32P28	LEPRE1	Prolyl 3-hydroxylase 1	7	1.857	2.4284	0.387
DGAT1_HUMAN	O75907	DGAT1	Diacylglycerol O-acyltransferase 1	3	0.7464	0.9743	0.384
PRDX4_HUMAN	Q13162	PRDX4	Peroxiredoxin-4	20	9.4233	12.253	0.379
GPC6_HUMAN	Q9Y625	GPC6	Glypican-6	2	0.4482	0.5827	0.379
STT3B_HUMAN	Q8TCJ2	STT3B	Dolichyl-diphosphooligosaccharide--protein glycosyltransferase subunit STT3B	19	9.3814	12.1764	0.376

NB5R3_HUMAN	P00387	CYB5R3	NADH-cytochrome b5 reductase 3	129	145.23	188.142	0.373
NDUA8_HUMAN	P51970	NDUFA8	NADH dehydrogenase [ubiquinone] 1 alpha subcomplex subunit 8	4	5.0873	6.5892	0.373
FACE1_HUMAN	O75844	ZMPSTE24	CAAX prenyl protease 1 homolog	5	1.1749	1.5183	0.370
EFR3A_HUMAN	Q14156	EFR3A	Protein EFR3 homolog A	11	3.7991	4.9046	0.368
RS2_HUMAN	P15880	RPS2	40S ribosomal protein S2	18	9.1614	11.8103	0.366
IBP5_HUMAN	P24593	IGFBP5	Insulin-like growth factor-binding protein 5	4	1.2023	1.5479	0.365
KAP0_HUMAN	P10644	PRKAR1A	cAMP-dependent protein kinase type I-alpha regulatory subunit	3	0.6434	0.8262	0.361
TMM43_HUMAN	Q9BTV4	TMEM43	Transmembrane protein 43	76	74.7556	95.7776	0.358
B4DPY0_HUMAN	B4DPY0	GPX8	Glutathione peroxidase	31	39.2838	50.2865	0.356
HYEP_HUMAN	P07099	EPHX1	Epoxide hydrolase 1	18	7.333	9.3719	0.354
GLU2B_HUMAN	P14314	PRKCSH	Glucosidase 2 subunit beta	11	8.3014	10.5516	0.346
SNP23_HUMAN	O00161	SNAP23	Synaptosomal-associated protein 23	11	3.2686	4.1218	0.335
CY1_HUMAN	P08574	CYC1	Cytochrome c1, heme protein, mitochondrial	5	2.0736	2.6086	0.331
COPE_HUMAN	O14579	COPE	Coatomer subunit epsilon	3	1.6304	2.0421	0.325
DPM1_HUMAN	O60762	DPM1	Dolichol-phosphate mannosyltransferase	8	4.5576	5.7032	0.323
ARL8B_HUMAN	Q9NVJ2	ARL8B	ADP-ribosylation factor-like protein 8B	12	6.393	7.9868	0.321
1433T_HUMAN	P27348	YWHAQ	14-3-3 protein theta	12	4.2874	5.3401	0.317
ITA2_HUMAN	P17301	ITGA2	Integrin alpha-2	2	1.0778	1.3409	0.315
THY1_HUMAN	P04216	THY1	Thy-1 membrane glycoprotein	87	210.276	261.163	0.313
NDUB9_HUMAN	Q9Y6M9	NDUFB9	NADH dehydrogenase [ubiquinone] 1 beta subcomplex subunit 9	5	1.6826	2.0896	0.313
GNAI1_HUMAN	P63096	GNAI1	Guanine nucleotide-binding protein G(i), alpha-1 subunit	76	65.6283	81.4775	0.312
GNAI2_HUMAN	P04899	GNAI2	Guanine nucleotide-binding protein G(i), alpha-2 subunit	117	101.185	125.365	0.309
AT2B1_HUMAN	P20020	ATP2B1	Plasma membrane calcium-transporting ATPase 1	25	17.0574	21.1141	0.308
MRP1_HUMAN	P33527	ABCC1	Multidrug resistance-associated protein 1	9	4.6593	5.7141	0.294
LRRC1_HUMAN	Q9BTT6	LRRC1	Leucine-rich repeat-containing protein 1	2	0.4486	0.5491	0.292
RALA_HUMAN	P11233	RALA	Ras-related protein Ral-A	17	7.9341	9.6892	0.288
GNA11_HUMAN	P29992	GNA11	Guanine nucleotide-binding protein subunit alpha-11	28	14.2853	17.2807	0.275
HS71L_HUMAN	P34931	HSPA1L	Heat shock 70 kDa protein 1L	25	14.6058	17.5776	0.267
PBIP1_HUMAN	Q96AQ6	PBXIP1	Pre-B-cell leukemia transcription factor-interacting protein 1	14	7.9827	9.5752	0.262
RGRF1_HUMAN	Q13972	RASGRF1	Ras-specific guanine nucleotide-releasing factor 1	2	1.0938	1.3108	0.261
MYO1B_HUMAN	O43795	MYO1B	Myosin-Ib	48	25.7057	30.6795	0.255
PHB_HUMAN	P35232	PHB	Prohibitin	43	23.0792	27.5163	0.254
LRC32_HUMAN	Q14392	LRRC32	Leucine-rich repeat-containing protein 32	4	1.3494	1.6035	0.249
ACTA_HUMAN	P62736	ACTA2	Actin, aortic smooth muscle	224	668.539	794.199	0.248
MBLC2_HUMAN	Q68D91	MBLAC2	Metallo-beta-lactamase domain-containing protein 2	2	0.7849	0.9321	0.248
TRPM4_HUMAN	Q8TD43	TRPM4	Transient receptor potential cation channel subfamily M member 4	4	1.9541	2.3076	0.240
LMNA_HUMAN	P02545	LMNA	Lamin-A/C	32	21.5675	25.4282	0.238
TM9S3_HUMAN	Q9HD45	TM9SF3	Transmembrane 9 superfamily member 3	4	2.1612	2.547	0.237
ANX11_HUMAN	P50995	ANXA11	Annexin A11	5	1.5924	1.8755	0.236

SGCD_HUMAN	Q92629	SGCD	Delta-sarcoglycan	9	5.5894	6.5752	0.234
RPN2_HUMAN	P04844	RPN2	Dolichyl-diphosphooligosaccharide--protein glycosyltransferase subunit 2	84	67.407	78.9994	0.229
MGST1_HUMAN	P10620	MGST1	Microsomal glutathione S-transferase 1	18	6.2217	7.2769	0.226
ACTG_HUMAN	P63261	ACTG1	Actin, cytoplasmic 2	406	1454.28	1694.81	0.221
ACTB_HUMAN	P60709	ACTB	Actin, cytoplasmic 1	402	1452.91	1692.81	0.220
UBIQ_HUMAN	P62988	RPS27A	Ubiquitin	29	66.4722	77.007	0.212
BASI_HUMAN	P35613	BSG	Basigin	20	26.8201	31.0319	0.210
RCN1_HUMAN	Q15293	RCN1	Reticulocalbin-1	5	5.26	6.0808	0.209
ATPO_HUMAN	P48047	ATP5O	ATP synthase subunit O, mitochondrial	22	15.8454	18.2812	0.206
ILK_HUMAN	Q13418	ILK	Integrin-linked protein kinase	4	1.6646	1.9175	0.204
GNAQ_HUMAN	P50148	GNAQ	Guanine nucleotide-binding protein G(q) subunit alpha	23	10.4509	12.0103	0.201
Q49AG2_HUMAN	Q49AG2	TMED5	TMED5 protein	4	1.1613	1.3321	0.198
LMAN2_HUMAN	Q12907	LMAN2	Vesicular integral-membrane protein VIP36	28	17.5233	20.0698	0.196
MLEC_HUMAN	Q14165	MLEC	Malectin	16	11.0596	12.6522	0.194
STXB3_HUMAN	O00186	STXBP3	Syntaxin-binding protein 3	4	1.2543	1.4315	0.191
AT1A1_HUMAN	P05023	ATP1A1	Sodium/potassium-transporting ATPase subunit alpha-1	73	87.4077	99.6832	0.190
EPHA2_HUMAN	P29317	EPHA2	Ephrin type-A receptor 2	19	11.6656	13.2922	0.188
PERI_HUMAN	P41219	PRPH	Peripherin	53	60.8584	69.2421	0.186
LEMD2_HUMAN	Q8NC56	LEMD2	LEM domain-containing protein 2	6	4.9463	5.594	0.178
HNRPK_HUMAN	P61978	HNRNPK	Heterogeneous nuclear ribonucleoprotein K	4	1.452	1.6407	0.176
MPIP1_HUMAN	P30304	CDC25A	M-phase inducer phosphatase 1	5	2.4463	2.7514	0.170
ACTC_HUMAN	P68032	ACTC1	Actin, alpha cardiac muscle 1	245	797.623	893.709	0.164
STX4_HUMAN	Q12846	STX4	Syntaxin-4	15	12.3406	13.7782	0.159
RL18_HUMAN	Q07020	RPL18	60S ribosomal protein L18	8	8.3209	9.2742	0.156
TOLIP_HUMAN	Q9H0E2	TOLLIP	Toll-interacting protein	2	0.388	0.4324	0.156
OPRS1_HUMAN	Q99720	OPRS1	Sigma 1-type opioid receptor	2	0.5889	0.65	0.142
RS7_HUMAN	P62081	RPS7	40S ribosomal protein S7	5	1.5143	1.671	0.142
RL7A_HUMAN	P62424	RPL7A	60S ribosomal protein L7a	21	18.1605	20.0376	0.142
GBB1_HUMAN	P62873	GNB1	Guanine nucleotide-binding protein G(I)/G(S)/G(T) subunit beta-1	80	75.6901	83.251	0.137
SC61G_HUMAN	P60059	SEC61G	Protein transport protein Sec61 subunit gamma	18	7.2498	7.9553	0.134
BAK_HUMAN	Q16611	BAK1	Bcl-2 homologous antagonist/killer	2	0.9316	1.0204	0.131
TRAM1_HUMAN	Q15629	TRAM1	Translocating chain-associated membrane protein 1	9	2.8439	3.113	0.130
HS90B_HUMAN	P08238	HSP90AB1	Heat shock protein HSP 90-beta	37	21.661	23.6796	0.129
VPP3_HUMAN	Q13488	TCIRG1	V-type proton ATPase 116 kDa subunit a isoform 3	4	1.2831	1.4022	0.128
PGFRB_HUMAN	P09619	PDGFRB	Beta-type platelet-derived growth factor receptor	82	57.0831	62.1748	0.123
RL24_HUMAN	P83731	RPL24	60S ribosomal protein L24	13	6.8762	7.4801	0.121
SREC2_HUMAN	Q96GP6	SCARF2	Scavenger receptor class F member 2	2	0.7518	0.8171	0.120
CE043_HUMAN	Q7Z3B0	C5orf43	UPF0542 protein C5orf43	3	0.8026	0.8719	0.119

DAG1_HUMAN	Q14118	DAG1	Dystroglycan	15	10.541	11.3799	0.110
RAB9A_HUMAN	P51151	RAB9A	Ras-related protein Rab-9A	4	0.8915	0.9613	0.109
ATPB_HUMAN	P06576	ATP5B	ATP synthase subunit beta, mitochondrial	13	3.6015	3.8798	0.107
Q5HYD9_HUMAN	Q5HYD9	CYB5B	Putative uncharacterized protein DKFZp686M0619 (Fragment)	20	26.5737	28.5128	0.102
NSMA3_HUMAN	Q9NXE4	SMPD4	Sphingomyelin phosphodiesterase 4	2	0.5736	0.6141	0.098
LNP_HUMAN	Q9C0E8	LNP	Protein lunapark	7	4.1344	4.399	0.089
MFF_HUMAN	Q9GZY8	MFF	Mitochondrial fission factor	2	0.434	0.4599	0.084
S10AD_HUMAN	Q99584	S100A13	Protein S100-A13	7	1.9073	2.0135	0.078
S39AE_HUMAN	Q15043	SLC39A14	Zinc transporter ZIP14	11	5.8952	6.2176	0.077
TOIP1_HUMAN	Q5JTV8	TOR1AIP1	Torsin-1A-interacting protein 1	17	10.2646	10.8137	0.075
NDUA9_HUMAN	Q16795	NDUFA9	NADH dehydrogenase [ubiquinone] 1 alpha subcomplex subunit 9, mitochondrial	11	5.5373	5.8102	0.069
LMAN1_HUMAN	P49257	LMAN1	Protein ERGIC-53	84	67.201	70.3503	0.066
CD59_HUMAN	P13987	CD59	CD59 glycoprotein	21	41.4165	43.3338	0.065
MPU1_HUMAN	O75352	MPDU1	Mannose-P-dolichol utilization defect 1 protein	4	1.5238	1.5874	0.059
SGCB_HUMAN	Q16585	SGCB	Beta-sarcoglycan	2	1.2619	1.3113	0.055
RAB5C_HUMAN	P51148	RAB5C	Ras-related protein Rab-5C	45	34.8642	36.1685	0.053
MOXD1_HUMAN	Q6UVY6	MOXD1	DBH-like monooxygenase protein 1	39	30.7488	31.8672	0.052
KAP2_HUMAN	P13861	PRKAR2A	cAMP-dependent protein kinase type II-alpha regulatory subunit	2	1.275	1.3195	0.049
CALD1_HUMAN	Q05682	CALD1	Caldesmon	39	88.29	90.9554	0.043
PDLI7_HUMAN	Q9NR12	PDLIM7	PDZ and LIM domain protein 7	6	2.532	2.6016	0.039
RASK_HUMAN	P01116	KRAS	GTPase KRas	10	8.4842	8.6782	0.033
FKBP8_HUMAN	Q14318	FKBP8	FK506-binding protein 8	10	5.2338	5.3413	0.029
ENPP1_HUMAN	P22413	ENPP1	Ectonucleotide pyrophosphatase/phosphodiesterase family member 1	55	33.2928	33.9457	0.028
UBE2N_HUMAN	P61088	UBE2N	Ubiquitin-conjugating enzyme E2 N	2	1.1614	1.1829	0.026
CAD11_HUMAN	P55287	CDH11	Cadherin-11	20	14.2563	14.5169	0.026
COX5A_HUMAN	P20674	COX5A	Cytochrome c oxidase subunit 5A, mitochondrial	4	3.0864	3.1353	0.023
T179B_HUMAN	Q7Z7N9	TMEM179B	Transmembrane protein 179B	2	0.2934	0.2967	0.016
ITA3_HUMAN	P26006	ITGA3	Integrin alpha-3	8	6.995	7.0651	0.014
Q6IPH7_HUMAN	Q6IPH7	RPL14	RPL14 protein	15	16.1905	16.2769	0.008
ABD12_HUMAN	Q8N2K0	ABHD12	Monoacylglycerol lipase ABHD12	4	2.504	2.5167	0.007
CP51A_HUMAN	Q16850	CYP51A1	Lanosterol 14-alpha demethylase	7	3.8715	3.8888	0.006
SCPDH_HUMAN	Q8NBX0	SCCPDH	Probable saccharopine dehydrogenase	8	3.8245	3.8152	-0.004
ERLN1_HUMAN	O75477	ERLIN1	Erlin-1	17	11.2467	11.1558	-0.012
PPIC_HUMAN	P45877	PPIC	Peptidyl-prolyl cis-trans isomerase C	2	1.3351	1.3229	-0.013
MYO1D_HUMAN	O94832	MYO1D	Myosin-Id	40	16.3328	16.1777	-0.014
FAM3C_HUMAN	Q92520	FAM3C	Protein FAM3C	12	5.587	5.5315	-0.014
RAB32_HUMAN	Q13637	RAB32	Ras-related protein Rab-32	5	4.6141	4.5438	-0.022
AT131_HUMAN	Q9HD20	ATP13A1	Probable cation-transporting ATPase 13A1	8	8.24	8.1116	-0.023

DCBD2_HUMAN	Q96PD2	DCBLD2	Discoidin, CUB and LCCL domain-containing protein 2	2	0.5741	0.5649	-0.023
ARPC2_HUMAN	O15144	ARPC2	Actin-related protein 2/3 complex subunit 2	6	3.8869	3.8183	-0.026
ITM2B_HUMAN	Q9Y287	ITM2B	Integral membrane protein 2B	2	0.5246	0.5152	-0.026
TCPG_HUMAN	P49368	CCT3	T-complex protein 1 subunit gamma	16	6.0492	5.9231	-0.030
RL5_HUMAN	P46777	RPL5	60S ribosomal protein L5	5	11.1873	10.9507	-0.031
PPIB_HUMAN	P23284	PPIB	Peptidyl-prolyl cis-trans isomerase B	94	85.9791	84.0146	-0.033
HSP71_HUMAN	P08107	HSPA1A	Heat shock 70 kDa protein 1	26	14.9548	14.6125	-0.033
FLNA_HUMAN	P21333	FLNA	Filamin-A	248	295.79	288.854	-0.034
PLXB2_HUMAN	O15031	PLXNB2	Plexin-B2	34	22.1692	21.6386	-0.035
OSTC_HUMAN	Q9NRP0	OSTC	Oligosaccharyltransferase complex subunit OSTC	2	0.6576	0.6391	-0.041
1433G_HUMAN	P61981	YWHAG	14-3-3 protein gamma	11	4.3995	4.2635	-0.045
SNAA_HUMAN	P54920	NAPA	Alpha-soluble NSF attachment protein	2	0.5765	0.5581	-0.047
CISD1_HUMAN	Q9NZ45	CISD1	CDGSH iron sulfur domain-containing protein 1	2	1.2268	1.1872	-0.047
PRDBP_HUMAN	Q969G5	PRKCDBP	Protein kinase C delta-binding protein	40	54.4263	52.3419	-0.056
H33_HUMAN	P84243	H3F3A	Histone H3.3	2	2.6616	2.55	-0.062
HP1B3_HUMAN	Q5SSJ5	HP1BP3	Heterochromatin protein 1-binding protein 3	2	0.5933	0.5669	-0.066
PON2_HUMAN	Q15165	PON2	Serum paraoxonase/arylesterase 2	5	5.536	5.2774	-0.069
SPNS1_HUMAN	Q9H2V7	SPNS1	Protein spinster homolog 1	2	0.4852	0.4622	-0.070
OST48_HUMAN	P39656	DDOST	Dolichyl-diphosphooligosaccharide--protein glycosyltransferase 48 kDa subunit	132	195.433	185.554	-0.075
PANX1_HUMAN	Q96RD7	PANX1	Pannexin-1	4	1.6093	1.5224	-0.080
COX2_HUMAN	P00403	MT-CO2	Cytochrome c oxidase subunit 2	15	17.908	16.9265	-0.081
CISD2_HUMAN	Q8N5K1	CISD2	CDGSH iron sulfur domain-containing protein 2	7	3.366	3.1739	-0.085
ANXA6_HUMAN	P08133	ANXA6	Annexin A6	282	221.457	208.379	-0.088
COPG_HUMAN	Q9Y678	COPG	Coatomer subunit gamma	5	1.802	1.6944	-0.089
ERO1A_HUMAN	Q96HE7	ERO1L	ERO1-like protein alpha	2	0.6879	0.6463	-0.090
ASPH_HUMAN	Q12797	ASPH	Aspartyl/asparaginyl beta-hydroxylase	68	62.8827	58.862	-0.095
K2C7_HUMAN	P08729	KRT7	Keratin, type II cytoskeletal 7	48	28.6328	26.799	-0.095
DDAH2_HUMAN	O95865	DDAH2	N(G),N(G)-dimethylarginine dimethylaminohydrolase 2	9	7.0355	6.5823	-0.096
APMAP_HUMAN	Q9HDC9	APMAP	Adipocyte plasma membrane-associated protein	48	57.941	54.115	-0.099
RS10_HUMAN	P46783	RPS10	40S ribosomal protein S10	4	1.1036	1.0268	-0.104
IDHC_HUMAN	O75874	IDH1	Isocitrate dehydrogenase [NADP] cytoplasmic	2	0.723	0.6725	-0.104
LMNB1_HUMAN	P20700	LMNB1	Lamin-B1	15	9.9953	9.2712	-0.108
PPGB_HUMAN	P10619	CTSA	Lysosomal protective protein	2	0.8475	0.7855	-0.110
EMD_HUMAN	P50402	EMD	Emerin	34	46.2675	42.7406	-0.114
SKP1_HUMAN	P63208	SKP1	S-phase kinase-associated protein 1	2	1.429	1.3167	-0.118
FAS_HUMAN	P49327	FASN	Fatty acid synthase	35	15.0151	13.8142	-0.120
SQSTM_HUMAN	Q13501	SQSTM1	Sequestosome-1	2	2.1905	2.0105	-0.124
S12A2_HUMAN	P55011	SLC12A2	Solute carrier family 12 member 2	8	4.3792	4.0151	-0.125

CALM_HUMAN	P62158	CALM1	Calmodulin	70	63.1701	57.8945	-0.126
PGRC1_HUMAN	O00264	PGRMC1	Membrane-associated progesterone receptor component 1	43	26.2378	24.0215	-0.127
RASN_HUMAN	P01111	NRAS	GTPase NRas	16	10.9714	10.0253	-0.130
BASP1_HUMAN	P80723	BASP1	Brain acid soluble protein 1	13	82.8329	75.422	-0.135
ANO6_HUMAN	Q4KMQ2	ANO6	Anoctamin-6	6	4.5001	4.0846	-0.140
AR6P6_HUMAN	Q8N6S5	ARL6IP6	ADP-ribosylation factor-like protein 6-interacting protein 6	2	1.0691	0.9685	-0.143
LPHN2_HUMAN	O95490	LPHN2	Latrophilin-2	2	0.5727	0.5184	-0.144
AT2B4_HUMAN	P23634	ATP2B4	Plasma membrane calcium-transporting ATPase 4	35	44.6346	40.3184	-0.147
NDUAC_HUMAN	Q9UI09	NDUFA12	NADH dehydrogenase [ubiquinone] 1 alpha subcomplex subunit 12	2	1.4803	1.3357	-0.148
GBB4_HUMAN	Q9HAV0	GNB4	Guanine nucleotide-binding protein subunit beta-4	48	56.8428	50.9502	-0.158
CD276_HUMAN	Q5ZPR3	CD276	CD276 antigen	4	6.8874	6.1639	-0.160
HMOX1_HUMAN	P09601	HMOX1	Heme oxygenase 1	3	1.3547	1.2085	-0.165
NDUS8_HUMAN	O00217	NDUFS8	NADH dehydrogenase [ubiquinone] iron-sulfur protein 8, mitochondrial	6	2.3466	2.091	-0.166
NDUAD_HUMAN	Q9P0J0	NDUFA13	NADH dehydrogenase [ubiquinone] 1 alpha subcomplex subunit 13	6	6.3222	5.6052	-0.174
PEX14_HUMAN	O75381	PEX14	Peroxisomal membrane protein PEX14	2	0.4109	0.3642	-0.174
TPSN_HUMAN	O15533	TAPBP	Tapasin	6	2.5042	2.2189	-0.175
GBB2_HUMAN	P62879	GNB2	Guanine nucleotide-binding protein G(I)/G(S)/G(T) subunit beta-2	80	95.6492	84.4723	-0.179
RL28_HUMAN	P46779	RPL28	60S ribosomal protein L28	10	9.7408	8.6024	-0.179
RER1_HUMAN	O15258	RER1	Protein RER1	2	3.8541	3.4005	-0.181
TMED2_HUMAN	Q15363	TMED2	Transmembrane emp24 domain-containing protein 2	20	9.3724	8.2453	-0.185
HMOX2_HUMAN	P30519	HMOX2	Heme oxygenase 2	20	14.5257	12.743	-0.189
RS20_HUMAN	P60866	RPS20	40S ribosomal protein S20	2	1.5615	1.3687	-0.190
VDAC1_HUMAN	P21796	VDAC1	Voltage-dependent anion-selective channel protein 1	78	79.6689	69.5534	-0.196
1C12_HUMAN	P30508	HLA-C	HLA class I histocompatibility antigen, Cw-12 alpha chain	82	53.9869	46.8827	-0.204
VIME_HUMAN	P08670	VIM	Vimentin	318	981.79	846.834	-0.213
E41L2_HUMAN	O43491	EPB41L2	Band 4.1-like protein 2	36	46.7733	40.3277	-0.214
NNMT_HUMAN	P40261	NNMT	Nicotinamide N-methyltransferase	5	2.7265	2.3507	-0.214
CAB45_HUMAN	Q9BRK5	SDF4	45 kDa calcium-binding protein	5	4.3628	3.7614	-0.214
TRPV2_HUMAN	Q9Y5S1	TRPV2	Transient receptor potential cation channel subfamily V member 2	3	2.5967	2.2362	-0.216
ACTZ_HUMAN	P61163	ACTR1A	Alpha-centractin	2	0.6693	0.575	-0.219
Q7L7Q6_HUMAN	Q7L7Q6	RTN4	RTN4 isoform B1	81	446.159	383.227	-0.219
CC50A_HUMAN	Q9NV96	TMEM30A	Cell cycle control protein 50A	2	0.5482	0.4696	-0.223
HNRPF_HUMAN	P52597	HNRNPF	Heterogeneous nuclear ribonucleoprotein F	2	0.4498	0.3846	-0.226
ATPA_HUMAN	P25705	ATP5A1	ATP synthase subunit alpha, mitochondrial	39	31.628	26.9854	-0.229
ATP5H_HUMAN	O75947	ATP5H	ATP synthase subunit d, mitochondrial	11	7.4282	6.3313	-0.231
TM87A_HUMAN	Q8NBN3	TMEM87A	Transmembrane protein 87A	2	1.4136	1.1971	-0.240
EF1A1_HUMAN	P68104	EEF1A1	Elongation factor 1-alpha 1	74	221.455	186.482	-0.248
RS6_HUMAN	P62753	RPS6	40S ribosomal protein S6	16	10.2277	8.5991	-0.250
VDAC3_HUMAN	Q9Y277	VDAC3	Voltage-dependent anion-selective channel protein 3	36	37.3622	31.4049	-0.251

PKHO2_HUMAN	Q8TD55	PLEKHO2	Pleckstrin homology domain-containing family O member 2	3	1.9945	1.6716	-0.255
IPO7_HUMAN	O95373	IPO7	Importin-7	4	1.0292	0.8617	-0.256
LAP2B_HUMAN	P42167	TMPO	Lamina-associated polypeptide 2, isoforms beta/gamma	29	32.8043	27.4524	-0.257
MPCP_HUMAN	Q00325	SLC25A3	Phosphate carrier protein, mitochondrial	35	31.6238	26.4585	-0.257
VDAC2_HUMAN	P45880	VDAC2	Voltage-dependent anion-selective channel protein 2	28	37.1454	31.0647	-0.258
S10AB_HUMAN	P31949	S100A11	Protein S100-A11	24	15.8854	13.2737	-0.259
STIM1_HUMAN	Q13586	STIM1	Stromal interaction molecule 1	5	2.8181	2.3481	-0.263
MYH9_HUMAN	P35579	MYH9	Myosin-9	160	143.116	119.032	-0.266
P4K2A_HUMAN	Q9BTU6	PI4K2A	Phosphatidylinositol 4-kinase type 2-alpha	2	1.7631	1.4573	-0.275
TM87B_HUMAN	Q96K49	TMEM87B	Transmembrane protein 87B	2	0.4658	0.384	-0.279
TM173_HUMAN	Q86WV6	TMEM173	Transmembrane protein 173	17	19.7541	16.2218	-0.284
CD44_HUMAN	P16070	CD44	CD44 antigen	102	116.355	95.5456	-0.284
RL6_HUMAN	Q02878	RPL6	60S ribosomal protein L6	15	13.7727	11.3059	-0.285
GDIB_HUMAN	P50395	GDI2	Rab GDP dissociation inhibitor beta	8	3.8297	3.1353	-0.289
VAMP3_HUMAN	Q15836	VAMP3	Vesicle-associated membrane protein 3	8	3.7752	3.0867	-0.290
RS9_HUMAN	P46781	RPS9	40S ribosomal protein S9	11	10.2138	8.341	-0.292
ILVBL_HUMAN	A1L0T0	ILVBL	Acetolactate synthase-like protein	11	13.2955	10.8536	-0.293
TMEDA_HUMAN	P49755	TMED10	Transmembrane emp24 domain-containing protein 10	62	118.29	96.0195	-0.301
RAB5B_HUMAN	P61020	RAB5B	Ras-related protein Rab-5B	27	27.462	22.251	-0.304
BAG2_HUMAN	O95816	BAG2	BAG family molecular chaperone regulator 2	4	0.998	0.8071	-0.306
MTCH1_HUMAN	Q9NZJ7	MTCH1	Mitochondrial carrier homolog 1	3	2.7961	2.2559	-0.310
Q54A51_HUMAN	Q54A51	BSG	Cervical EMMPRIN	35	63.3796	51.0475	-0.312
ACAM_HUMAN	Q9H6B4	ACAM	Adipocyte adhesion molecule	3	1.0982	0.8832	-0.314
RL23_HUMAN	P62829	RPL23	60S ribosomal protein L23	5	2.0445	1.6408	-0.317
FARP1_HUMAN	Q9Y4F1	FARP1	FERM, RhoGEF and pleckstrin domain-containing protein 1	10	4.8947	3.9208	-0.320
VAPA_HUMAN	Q9P0L0	VAPA	Vesicle-associated membrane protein-associated protein A	18	40.3231	32.2947	-0.320
AR6P1_HUMAN	Q15041	ARL6IP1	ADP-ribosylation factor-like protein 6-interacting protein 1	2	0.8378	0.6708	-0.321
ANXA2_HUMAN	P07355	ANXA2	Annexin A2	744	2233.84	1781.56	-0.326
PLXD1_HUMAN	Q9Y4D7	PLXND1	Plexin-D1	5	1.235	0.9833	-0.329
NPTN_HUMAN	Q9Y639	NPTN	Neuroplastin	11	12.7585	10.1489	-0.330
FLOT2_HUMAN	Q14254	FLOT2	Flotillin-2	26	11.4698	9.1149	-0.332
SCRIB_HUMAN	Q14160	SCRIB	Protein scribble homolog	4	1.1092	0.8761	-0.340
MYPT1_HUMAN	O14974	PPP1R12A	Protein phosphatase 1 regulatory subunit 12A	2	0.9635	0.761	-0.340
RL4_HUMAN	P36578	RPL4	60S ribosomal protein L4	21	23.8172	18.7169	-0.348
A8K5C2_HUMAN	A8K5C2	KIAA1609	cDNA FLJ75055	10	5.3108	4.1454	-0.357
USE1_HUMAN	Q9NZ43	USE1	Vesicle transport protein USE1	2	0.8325	0.6489	-0.359
RL13A_HUMAN	P40429	RPL13A	60S ribosomal protein L13a	4	4.8401	3.7705	-0.360
RAB5A_HUMAN	P20339	RAB5A	Ras-related protein Rab-5A	26	31.8494	24.6327	-0.371
HS90A_HUMAN	P07900	HSP90AA1	Heat shock protein HSP 90-alpha	38	22.6472	17.495	-0.372

CD9_HUMAN	P21926	CD9	CD9 antigen	5	7.7088	5.94	-0.376
SFXN1_HUMAN	Q9H9B4	SFXN1	Sideroflexin-1	15	9.7594	7.5196	-0.376
RAB18_HUMAN	Q9NP72	RAB18	Ras-related protein Rab-18	12	7.3018	5.6253	-0.376
MMP2_HUMAN	P08253	MMP2	72 kDa type IV collagenase	2	0.7092	0.5446	-0.381
PTRF_HUMAN	Q6NZI2	PTRF	Polymerase I and transcript release factor	105	105.16	80.7452	-0.381
ACTN1_HUMAN	P12814	ACTN1	Alpha-actinin-1	54	27.4284	21.0058	-0.385
ICAM1_HUMAN	P05362	ICAM1	Intercellular adhesion molecule 1	9	4.7495	3.6183	-0.392
RLA2_HUMAN	P05387	RPLP2	60S acidic ribosomal protein P2	10	5.1115	3.8848	-0.396
VKOR1_HUMAN	Q9BQB6	VKORC1	Vitamin K epoxide reductase complex subunit 1	3	1.749	1.3266	-0.399
CS010_HUMAN	Q969H8	C19orf10	UPF0556 protein C19orf10	3	3.1471	2.3854	-0.400
K1C19_HUMAN	P08727	KRT19	Keratin, type I cytoskeletal 19	12	8.102	6.1333	-0.402
CYFP1_HUMAN	Q7L576	CYFIP1	Cytoplasmic FMR1-interacting protein 1	9	3.0415	2.2977	-0.405
SE1L1_HUMAN	Q9UBV2	SEL1L	Protein sel-1 homolog 1	4	0.9805	0.7396	-0.407
SFXN3_HUMAN	Q9BWM7	SFXN3	Sideroflexin-3	17	9.7573	7.3507	-0.409
TMED5_HUMAN	Q9Y3A6	TMED5	Transmembrane emp24 domain-containing protein 5	6	2.5255	1.9017	-0.409
FNDC1_HUMAN	Q4ZHG4	FNDC1	Fibronectin type III domain-containing protein 1	3	1.9957	1.4946	-0.417
AHNK_HUMAN	Q09666	AHNAK	Neuroblast differentiation-associated protein AHNAK	1047	2374.67	1772.73	-0.422
Q29669_HUMAN	Q29669	HLA-B35	MHC class I antigen HLA-B35	91	73.547	54.7107	-0.427
RL17_HUMAN	P18621	RPL17	60S ribosomal protein L17	12	8.3456	6.1875	-0.432
SYPL1_HUMAN	Q16563	SYPL1	Synaptophysin-like protein 1	4	1.4549	1.0752	-0.436
MTX1_HUMAN	Q13505	MTX1	Metaxin-1	2	1.0983	0.8095	-0.440
UGGG1_HUMAN	Q9NYU2	UGGT1	UDP-glucose:glycoprotein glucosyltransferase 1	9	3.8484	2.8348	-0.441
RHOA_HUMAN	P61586	RHOA	Transforming protein RhoA	30	20.3659	14.9512	-0.446
SFPQ_HUMAN	P23246	SFPQ	Splicing factor, proline- and glutamine-rich	2	1.6884	1.2392	-0.446
AT1B3_HUMAN	P54709	ATP1B3	Sodium/potassium-transporting ATPase subunit beta-3	20	32.9048	24.0733	-0.451
HSPB1_HUMAN	P04792	HSPB1	Heat shock protein beta-1	15	26.9229	19.6727	-0.453
ANPRB_HUMAN	P20594	NPR2	Atrial natriuretic peptide receptor B	9	3.4408	2.5077	-0.456
BAX_HUMAN	Q07812	BAX	Apoptosis regulator BAX	2	0.5684	0.4133	-0.460
NDUA3_HUMAN	O95167	NDUFA3	NADH dehydrogenase [ubiquinone] 1 alpha subcomplex subunit 3	5	2.7519	2.0007	-0.460
NOTC2_HUMAN	Q04721	NOTCH2	Neurogenic locus notch homolog protein 2	16	21.7897	15.8355	-0.460
VKORL_HUMAN	Q8N0U8	VKORC1L1	Vitamin K epoxide reductase complex subunit 1-like protein 1	4	1.6691	1.2098	-0.464
SEPT9_HUMAN	Q9UHD8	SEPT9	Septin-9	4	4.5974	3.3265	-0.467
PSMD6_HUMAN	Q15008	PSMD6	26S proteasome non-ATPase regulatory subunit 6	2	1.4685	1.0609	-0.469
RAB34_HUMAN	Q9BZG1	RAB34	Ras-related protein Rab-34	2	0.2735	0.1967	-0.476
B4DMH5_HUMAN	B4DMH5	CDC42	cDNA FLJ55107, highly similar to Cell division control protein 42 homolog	26	24.1945	17.3975	-0.476
SCRB2_HUMAN	Q14108	SCARB2	Lysosome membrane protein 2	27	20.1086	14.4356	-0.478
ITA4_HUMAN	P13612	ITGA4	Integrin alpha-4	4	1.7479	1.2508	-0.483
HAS1_HUMAN	Q92839	HAS1	Hyaluronan synthase 1	2	0.8172	0.5845	-0.483

FSCN1_HUMAN	Q16658	FSCN1	Fascin	6	5.4744	3.9127	-0.485
Q4W6C4_HUMAN	Q4W6C4	HLA-B	MHC class I antigen (Fragment)	83	65.4695	46.5898	-0.491
MARCS_HUMAN	P29966	MARCKS	Myristoylated alanine-rich C-kinase substrate	48	137.649	97.8469	-0.492
CS052_HUMAN	Q9BSF4	C19orf52	Uncharacterized protein C19orf52	2	0.7002	0.4959	-0.498
S10AG_HUMAN	Q96FQ6	S100A16	Protein S100-A16	3	0.8335	0.5869	-0.506
YIF1A_HUMAN	O95070	YIF1A	Protein YIF1A	11	6.523	4.5491	-0.520
SPCS3_HUMAN	P61009	SPCS3	Signal peptidase complex subunit 3	5	1.8813	1.3113	-0.521
FERM2_HUMAN	Q96AC1	FERMT2	Fermitin family homolog 2	5	2.6627	1.8538	-0.522
TPP1_HUMAN	O14773	TPP1	Tripeptidyl-peptidase 1	3	0.651	0.4511	-0.529
NNTM_HUMAN	Q13423	NNT	NAD(P) transhydrogenase, mitochondrial	19	8.983	6.221	-0.530
PSD11_HUMAN	O00231	PSMD11	26S proteasome non-ATPase regulatory subunit 11	2	0.7461	0.5163	-0.531
RAB13_HUMAN	P51153	RAB13	Ras-related protein Rab-13	4	1.3819	0.956	-0.532
Q8MGZ8_HUMAN	Q8MGZ8	HLA-B	MHC class I antigen (Fragment)	92	73.3066	50.6707	-0.533
QCR1_HUMAN	P31930	UQCRC1	Cytochrome b-c1 complex subunit 1, mitochondrial	16	10.8515	7.4828	-0.536
RL22_HUMAN	P35268	RPL22	60S ribosomal protein L22	5	3.2026	2.2071	-0.537
QCR8_HUMAN	O14949	UQCRQ	Cytochrome b-c1 complex subunit 8	4	1.4783	1.0178	-0.538
ZNT9_HUMAN	Q6PML9	SLC30A9	Zinc transporter 9	2	0.9391	0.6451	-0.542
ANXA4_HUMAN	P09525	ANXA4	Annexin A4	15	7.1595	4.9124	-0.543
AT5F1_HUMAN	P24539	ATP5F1	ATP synthase subunit b, mitochondrial	33	29.0187	19.8656	-0.547
ITA5_HUMAN	P08648	ITGA5	Integrin alpha-5	54	53.1691	36.379	-0.547
ATP5L_HUMAN	O75964	ATP5L	ATP synthase subunit g, mitochondrial	4	1.1621	0.7932	-0.551
B3KUB6_HUMAN	B3KUB6	EPB41L1	cDNA FLJ39529 fis, clone PUAEN2004067, highly similar to Band 4.1-like protein 1	5	6.1497	4.1841	-0.556
XRP2_HUMAN	O75695	RP2	Protein XRP2	7	4.5779	3.1134	-0.556
B2R7T6_HUMAN	B2R7T6	SQRDL	cDNA, FLJ93596, highly similar to Homo sapiens sulfide quinone reductase-like (yeast) (SQRDL), mRNA	14	15.7931	10.6746	-0.565
H15_HUMAN	P16401	HIST1H1B	Histone H1.5	3	3.4569	2.3295	-0.569
SEP11_HUMAN	Q9NVA2	SEPT11	Septin-11	16	23.9772	16.1333	-0.572
RS24_HUMAN	P62847	RPS24	40S ribosomal protein S24	2	0.6881	0.4619	-0.575
VASN_HUMAN	Q6EMK4	VASN	Vasorin	13	8.7202	5.8523	-0.575
RS4X_HUMAN	P62701	RPS4X	40S ribosomal protein S4, X isoform	2	0.698	0.4682	-0.576
SC23A_HUMAN	Q15436	SEC23A	Protein transport protein Sec23A	3	0.8052	0.5376	-0.583
NDUS1_HUMAN	P28331	NDUFS1	NADH-ubiquinone oxidoreductase 75 kDa subunit, mitochondrial	3	0.8781	0.5861	-0.583
PLXA1_HUMAN	Q9UIW2	PLXNA1	Plexin-A1	8	3.6884	2.4568	-0.586
LMNB2_HUMAN	Q03252	LMNB2	Lamin-B2	15	9.0024	5.9772	-0.591
PTPRD_HUMAN	P23468	PTPRD	Receptor-type tyrosine-protein phosphatase delta	2	1.4756	0.9769	-0.595
PGS1_HUMAN	P21810	BGN	Biglycan	4	2.6584	1.7425	-0.609
TAP2_HUMAN	Q03519	TAP2	Antigen peptide transporter 2	3	0.8762	0.5743	-0.609
MRC2_HUMAN	Q9UBG0	MRC2	C-type mannose receptor 2	77	97.5048	63.2833	-0.624

MYH10_HUMAN	P35580	MYH10	Myosin-10	14	11.1215	7.2077	-0.626
PPBT_HUMAN	P05186	ALPL	Alkaline phosphatase, tissue-nonspecific isozyme	4	1.7962	1.1627	-0.627
ARPC3_HUMAN	O15145	ARPC3	Actin-related protein 2/3 complex subunit 3	4	2.9205	1.8855	-0.631
LANC1_HUMAN	O43813	LANCL1	LanC-like protein 1	2	0.8694	0.5581	-0.639
PCDG4_HUMAN	Q9Y5G9	PCDHGA4	Protocadherin gamma-A4	2	1.5838	1.0154	-0.641
F162A_HUMAN	Q96A26	FAM162A	UPF0389 protein FAM162A	3	2.1864	1.3974	-0.646
RADI_HUMAN	P35241	RDX	Radixin	11	8.5498	5.4442	-0.651
GCN1L_HUMAN	Q92616	GCN1L1	Translational activator GCN1	6	2.686	1.7062	-0.655
RL10_HUMAN	P27635	RPL10	60S ribosomal protein L10	5	4.1609	2.6375	-0.658
CCD56_HUMAN	Q9Y2R0	CCDC56	Coiled-coil domain-containing protein 56	2	0.4595	0.2911	-0.659
NDUB5_HUMAN	O43674	NDUFB5	NADH dehydrogenase [ubiquinone] 1 beta subcomplex subunit 5, mitochondrial	7	3.3977	2.1517	-0.659
B5A9M9_HUMAN	B5A9M9	HLA-A	MHC class I antigen (Fragment)	93	88.0525	55.6631	-0.662
RECK_HUMAN	O95980	RECK	Reversion-inducing cysteine-rich protein with Kazal motifs	21	20.944	13.2235	-0.663
RS16_HUMAN	P62249	RPS16	40S ribosomal protein S16	18	20.6846	13.0453	-0.665
ACTN4_HUMAN	O43707	ACTN4	Alpha-actinin-4	55	31.6817	19.9682	-0.666
DYHC1_HUMAN	Q14204	DYNC1H1	Cytoplasmic dynein 1 heavy chain 1	19	8.6964	5.479	-0.667
CPNE2_HUMAN	Q96FN4	CPNE2	Copine-2	2	1.2184	0.7619	-0.677
MCFD2_HUMAN	Q8NI22	MCFD2	Multiple coagulation factor deficiency protein 2	2	1.6816	1.0513	-0.678
ERLN2_HUMAN	O94905	ERLIN2	Erlin-2	27	35.8734	22.4258	-0.678
ANO10_HUMAN	Q9NW15	ANO10	Anoctamin-10	8	8.6672	5.4162	-0.678
COX6C_HUMAN	P09669	COX6C	Cytochrome c oxidase subunit 6C	7	6.7742	4.2318	-0.679
H12_HUMAN	P16403	HIST1H1C	Histone H1.2	23	25.7493	16.033	-0.683
EZRI_HUMAN	P15311	EZR	Ezrin	13	9.7353	6.0527	-0.686
HSP7C_HUMAN	P11142	HSPA8	Heat shock cognate 71 kDa protein	71	66.7976	41.2102	-0.697
CYB5_HUMAN	P00167	CYB5A	Cytochrome b5	11	11.8274	7.2687	-0.702
TCPE_HUMAN	P48643	CCT5	T-complex protein 1 subunit epsilon	5	2.4959	1.5331	-0.703
Q32Q12_HUMAN	Q32Q12	NME2	Nucleoside diphosphate kinase	13	12.5596	7.6914	-0.707
RL7_HUMAN	P18124	RPL7	60S ribosomal protein L7	18	9.0204	5.4816	-0.719
1433E_HUMAN	P62258	YWHAE	14-3-3 protein epsilon	13	5.7764	3.5049	-0.721
B4DDF9_HUMAN	B4DDF9	ANXA4	cDNA FLJ52218, highly similar to Annexin A4	7	3.171	1.9232	-0.721
1B58_HUMAN	P10319	HLA-B	HLA class I histocompatibility antigen, B-58 alpha chain	91	74.9493	45.4432	-0.722
PLPL6_HUMAN	Q8IY17	PNPLA6	Neuropathy target esterase	9	4.3788	2.6461	-0.727
BAP31_HUMAN	P51572	BCAP31	B-cell receptor-associated protein 31	8	3.0285	1.821	-0.734
KPCA_HUMAN	P17252	PRKCA	Protein kinase C alpha type	8	3.105	1.8648	-0.736
AL3A2_HUMAN	P51648	ALDH3A2	Fatty aldehyde dehydrogenase	3	1.0727	0.6425	-0.739
RS11_HUMAN	P62280	RPS11	40S ribosomal protein S11	4	3.0856	1.8306	-0.753
B7Z9B1_HUMAN	B7Z9B1	CDH13	cDNA FLJ52398, highly similar to Cadherin-13	15	11.9991	7.1054	-0.756
ARF5_HUMAN	P84085	ARF5	ADP-ribosylation factor 5	5	1.7668	1.0448	-0.758

AMPN_HUMAN	P15144	ANPEP	Aminopeptidase N	423	758.903	448.534	-0.759
1433Z_HUMAN	P63104	YWHAZ	14-3-3 protein zeta/delta	17	9.0909	5.3634	-0.761
PGFRA_HUMAN	P16234	PDGFRA	Alpha-type platelet-derived growth factor receptor	5	2.2177	1.3053	-0.765
PLEC1_HUMAN	Q15149	PLEC1	Plectin-1	34	21.8876	12.7483	-0.780
QCR2_HUMAN	P22695	UQCRC2	Cytochrome b-c1 complex subunit 2, mitochondrial	19	16.178	9.4121	-0.781
RAP2B_HUMAN	P61225	RAP2B	Ras-related protein Rap-2b	20	13.5077	7.8224	-0.788
EGFR_HUMAN	P00533	EGFR	Epidermal growth factor receptor	9	5.3386	3.0877	-0.790
LDHA_HUMAN	P00338	LDHA	L-lactate dehydrogenase A chain	20	12.6797	7.3324	-0.790
CO6A1_HUMAN	P12109	COL6A1	Collagen alpha-1(VI) chain	10	3.8937	2.2474	-0.793
RALB_HUMAN	P11234	RALB	Ras-related protein Ral-B	10	6.7268	3.8695	-0.798
NP1L1_HUMAN	P55209	NAP1L1	Nucleosome assembly protein 1-like 1	4	3.2529	1.8641	-0.803
MMP14_HUMAN	P50281	MMP14	Matrix metalloproteinase-14	17	27.7261	15.8677	-0.805
MFN2_HUMAN	O95140	MFN2	Mitofusin-2	2	0.4531	0.2584	-0.810
T161A_HUMAN	Q9NX61	TMEM161A	Transmembrane protein 161A	2	1.1412	0.6446	-0.824
POK2_HUMAN	Q9BXR3	ERVK6	HERV-K_7p22.1 provirus ancestral Pol protein	5	10.8957	6.1418	-0.827
RS8_HUMAN	P62241	RPS8	40S ribosomal protein S8	20	17.6766	9.9479	-0.829
TMED1_HUMAN	Q13445	TMED1	Transmembrane emp24 domain-containing protein 1	5	1.191	0.6691	-0.832
C109A_HUMAN	Q8NE86	CCDC109A	Coiled-coil domain-containing protein 109A	3	0.783	0.4392	-0.834
CAZA1_HUMAN	P52907	CAPZA1	F-actin-capping protein subunit alpha-1	8	5.8999	3.3035	-0.837
ACLY_HUMAN	P53396	ACLY	ATP-citrate synthase	12	5.5679	3.108	-0.841
H4_HUMAN	P62805	HIST1H4A	Histone H4	79	182.01	100.985	-0.850
WDR1_HUMAN	O75083	WDR1	WD repeat-containing protein 1	8	11.9489	6.6215	-0.852
ML12A_HUMAN	P19105	MYL12A	Myosin regulatory light chain 12A	10	12.0659	6.6596	-0.857
SYRC_HUMAN	P54136	RARS	Arginyl-tRNA synthetase, cytoplasmic	3	0.8245	0.4497	-0.875
TLN1_HUMAN	Q9Y490	TLN1	Talin-1	80	53.1186	28.9692	-0.875
A8ILL3_HUMAN	A8ILL3	HLA-A	MHC class I antigen (Fragment)	83	105.954	57.0602	-0.893
RL21_HUMAN	P46778	RPL21	60S ribosomal protein L21	6	13.0273	6.9832	-0.900
COR1B_HUMAN	Q9BR76	CORO1B	Coronin-1B	2	0.5384	0.2883	-0.901
FLNC_HUMAN	Q14315	FLNC	Filamin-C	100	123.916	66.3373	-0.901
CAZA2_HUMAN	P47755	CAPZA2	F-actin-capping protein subunit alpha-2	7	4.8035	2.5703	-0.902
CNTP1_HUMAN	P78357	CNTNAP1	Contactin-associated protein 1	4	2.2632	1.2058	-0.908
OCC1_HUMAN	Q8TAD7	OCC1	Putative overexpressed in colon carcinoma 1 protein	3	2.2451	1.1864	-0.920
B2L13_HUMAN	Q9BXK5	BCL2L13	Bcl-2-like protein 13	2	1.406	0.7428	-0.921
B2RDY9_HUMAN	B2RDY9	CAP1	Adenylyl cyclase-associated protein	15	9.533	5.0206	-0.925
RAP2C_HUMAN	Q9Y3L5	RAP2C	Ras-related protein Rap-2c	14	9.4286	4.9558	-0.928
DDR2_HUMAN	Q16832	DDR2	Discoidin domain-containing receptor 2	3	2.9239	1.5301	-0.934
STOM_HUMAN	P27105	STOM	Erythrocyte band 7 integral membrane protein	22	29.1603	15.1857	-0.941
Q1EJP6_HUMAN	Q1EJP6	HLA-A	MHC class I antigen (Fragment)	95	96.3954	49.7194	-0.955
PALLD_HUMAN	Q8WX93	PALLD	Palladin	2	2.1222	1.0942	-0.956

MYADM_HUMAN	Q96S97	MYADM	Myeloid-associated differentiation marker	6	11.2227	5.7422	-0.967
PDC6I_HUMAN	Q8WUM4	PDCD6IP	Programmed cell death 6-interacting protein	5	1.6402	0.8388	-0.967
Q52YL7_HUMAN	Q52YL7	HLA-A	MHC class I antigen	91	121.116	61.7276	-0.972
B1B6Q4_HUMAN	B1B6Q4	HLA-A	MHC class I antigen (Fragment)	85	116.365	59.2426	-0.974
ANXA1_HUMAN	P04083	ANXA1	Annexin A1	94	88.3046	44.6519	-0.984
CAV2_HUMAN	P51636	CAV2	Caveolin-2	3	1.2271	0.6193	-0.987
PRDX1_HUMAN	Q06830	PRDX1	Peroxiredoxin-1	19	20.6329	10.4091	-0.987
2AAA_HUMAN	P30153	PPP2R1A	Serine/threonine-protein phosphatase 2A 65 kDa regulatory subunit A alpha isoform	2	2.0253	1.021	-0.988
TM109_HUMAN	Q9BVC6	TMEM109	Transmembrane protein 109	10	27.4707	13.8263	-0.990
TERA_HUMAN	P55072	VCP	Transitional endoplasmic reticulum ATPase	21	8.2558	4.1235	-1.002
ERGI1_HUMAN	Q969X5	ERGIC1	Endoplasmic reticulum-Golgi intermediate compartment protein 1	9	4.9313	2.4604	-1.003
PPIA_HUMAN	P62937	PPIA	Peptidyl-prolyl cis-trans isomerase A	32	24.6804	12.276	-1.008
SEPT7_HUMAN	Q16181	SEPT7	Septin-7	11	11.3928	5.6506	-1.012
GRP75_HUMAN	P38646	HSPA9	Stress-70 protein, mitochondrial	7	7.5356	3.7321	-1.014
RASEF_HUMAN	Q8IZ41	RASEF	RAS and EF-hand domain-containing protein	4	1.5188	0.7463	-1.025
TM119_HUMAN	Q4V9L6	TMEM119	Transmembrane protein 119	13	30.8449	15.0882	-1.032
UBA1_HUMAN	P22314	UBA1	Ubiquitin-like modifier-activating enzyme 1	3	1.5347	0.7507	-1.032
Q86X69_HUMAN	Q86X69	PON2	PON2 protein	3	1.781	0.87	-1.034
DAB2_HUMAN	P98082	DAB2	Disabled homolog 2	3	1.6092	0.7816	-1.042
EHD1_HUMAN	Q9H4M9	EHD1	EH domain-containing protein 1	5	23.8189	11.5461	-1.045
PCYOX_HUMAN	Q9UHG3	PCYOX1	Prenylcysteine oxidase 1	54	67.5116	32.3523	-1.061
S10A6_HUMAN	P06703	S100A6	Protein S100-A6	6	5.5335	2.6397	-1.068
TMOD3_HUMAN	Q9NYL9	TMOD3	Tropomodulin-3	2	1.7234	0.8182	-1.075
SGCE_HUMAN	O43556	SGCE	Epsilon-sarcoglycan	5	3.3436	1.5817	-1.080
NRP1_HUMAN	O14786	NRP1	Neuropilin-1	7	13.8994	6.5601	-1.083
A8K968_HUMAN	A8K968	EPB41L3	cDNA FLJ77757	32	39.0312	18.3455	-1.089
VAT1_HUMAN	Q99536	VAT1	Synaptic vesicle membrane protein VAT-1 homolog	12	13.1203	6.1597	-1.091
ATPG_HUMAN	P36542	ATP5C1	ATP synthase subunit gamma, mitochondrial	8	8.8272	4.0609	-1.120
MAP4_HUMAN	P27816	MAP4	Microtubule-associated protein 4	2	1.1316	0.5185	-1.126
SNTB2_HUMAN	Q13425	SNTB2	Beta-2-syntrophin	8	4.6293	2.1211	-1.126
VINC_HUMAN	P18206	VCL	Vinculin	27	23.874	10.6127	-1.170
EF2_HUMAN	P13639	EEF2	Elongation factor 2	36	43.172	19.0292	-1.182
EHD2_HUMAN	Q9NZN4	EHD2	EH domain-containing protein 2	19	34.0181	14.95	-1.186
RL38_HUMAN	P63173	RPL38	60S ribosomal protein L38	2	1.1536	0.5029	-1.198
S10AA_HUMAN	P60903	S100A10	Protein S100-A10	104	345.839	150.174	-1.203
RAP2A_HUMAN	P10114	RAP2A	Ras-related protein Rap-2a	12	9.3906	4.0519	-1.213
TBB6_HUMAN	Q9BUF5	TUBB6	Tubulin beta-6 chain	128	164.4	70.8742	-1.214
CHMP6_HUMAN	Q96FZ7	CHMP6	Charged multivesicular body protein 6	2	0.9213	0.3958	-1.219

COPB_HUMAN	P53618	COPB1	Coatomer subunit beta	7	2.6107	1.1058	-1.239
TBB4_HUMAN	P04350	TUBB4	Tubulin beta-4 chain	183	282.878	119.657	-1.241
RINI_HUMAN	P13489	RNH1	Ribonuclease inhibitor	5	2.1298	0.8995	-1.244
TBB2C_HUMAN	P68371	TUBB2C	Tubulin beta-2C chain	197	309.898	130.828	-1.244
TBB2A_HUMAN	Q13885	TUBB2A	Tubulin beta-2A chain	171	252.571	106.272	-1.249
KINH_HUMAN	P33176	KIF5B	Kinesin-1 heavy chain	3	0.9119	0.3835	-1.250
CMC1_HUMAN	O75746	SLC25A12	Calcium-binding mitochondrial carrier protein Aralar1	3	1.1041	0.4633	-1.253
C9JGY8_HUMAN	C9JGY8	JAZF1	Putative uncharacterized protein JAZF1	2	1.9385	0.8009	-1.275
GSTP1_HUMAN	P09211	GSTP1	Glutathione S-transferase P	11	7.1453	2.9331	-1.285
FLNB_HUMAN	O75369	FLNB	Filamin-B	52	55.684	22.5859	-1.302
TBB5_HUMAN	P07437	TUBB	Tubulin beta chain	236	381.145	154.192	-1.306
TITIN_HUMAN	Q8WZ42	TTN	Titin	2	0.5373	0.2168	-1.309
ARP3_HUMAN	P61158	ACTR3	Actin-related protein 3	7	3.4541	1.3817	-1.322
SYNC_HUMAN	O43776	NARS	Asparaginyl-tRNA synthetase, cytoplasmic	3	1.0891	0.4267	-1.352
CN37_HUMAN	P09543	CNP	2~,3~-cyclic-nucleotide 3~-phosphodiesterase	3	1.7879	0.699	-1.355
ARF1_HUMAN	P84077	ARF1	ADP-ribosylation factor 1	12	7.3307	2.8443	-1.366
5NTD_HUMAN	P21589	NT5E	5~-nucleotidase	186	405.904	157.324	-1.367
MVP_HUMAN	Q14764	MVP	Major vault protein	18	20.6783	7.8638	-1.395
KPYM_HUMAN	P14618	PKM2	Pyruvate kinase isozymes M1/M2	127	132.381	50.0873	-1.402
SERA_HUMAN	O43175	PHGDH	D-3-phosphoglycerate dehydrogenase	3	1.6725	0.6327	-1.402
PTGIS_HUMAN	Q16647	PTGIS	Prostacyclin synthase	70	78.6646	29.5566	-1.412
TBB3_HUMAN	Q13509	TUBB3	Tubulin beta-3 chain	161	285.44	107.076	-1.415
MX2_HUMAN	P20592	MX2	Interferon-induced GTP-binding protein Mx2	3	1.0925	0.4084	-1.420
COR1C_HUMAN	Q9ULV4	CORO1C	Coronin-1C	15	7.7984	2.8892	-1.433
SEPT2_HUMAN	Q15019	SEPT2	Septin-2	16	18.8087	6.963	-1.434
VA0D1_HUMAN	P61421	ATP6V0D1	V-type proton ATPase subunit d 1	3	2.0849	0.7694	-1.438
IF5A1_HUMAN	P63241	EIF5A	Eukaryotic translation initiation factor 5A-1	5	5.9778	2.2021	-1.441
PARK7_HUMAN	Q99497	PARK7	Protein DJ-1	2	2.6127	0.9478	-1.463
TM138_HUMAN	Q9NPI0	TMEM138	Transmembrane protein 138	2	1.9853	0.7184	-1.466
GGT5_HUMAN	P36269	GGT5	Gamma-glutamyltransferase 5	6	2.5278	0.9097	-1.474
RFTN1_HUMAN	Q14699	RFTN1	Raftlin	14	13.5388	4.8298	-1.487
Q53XZ0_HUMAN	Q53XZ0	IFITM1	Interferon induced transmembrane protein 1 (9-27)	4	6.6181	2.321	-1.512
K2C8_HUMAN	P05787	KRT8	Keratin, type II cytoskeletal 8	18	11.27	3.9496	-1.513
GBG12_HUMAN	Q9UBI6	GNG12	Guanine nucleotide-binding protein G(I)/G(S)/G(O) subunit gamma-12	42	159.035	55.708	-1.513
MOES_HUMAN	P26038	MSN	Moesin	24	27.3741	9.3275	-1.553
AMRP_HUMAN	P30533	LRPAP1	Alpha-2-macroglobulin receptor-associated protein	3	2.7584	0.9302	-1.568
TCPZ_HUMAN	P40227	CCT6A	T-complex protein 1 subunit zeta	4	2.4753	0.8294	-1.577
VASP_HUMAN	P50552	VASP	Vasodilator-stimulated phosphoprotein	2	0.5581	0.1855	-1.589
TBA1B_HUMAN	P68363	TUBA1B	Tubulin alpha-1B chain	183	380.958	126.224	-1.594

LEG1_HUMAN	P09382	LGALS1	Galectin-1	22	20.0454	6.6133	-1.600
GPDM_HUMAN	P43304	GPD2	Glycerol-3-phosphate dehydrogenase, mitochondrial	13	7.0185	2.3116	-1.602
TPT1L_HUMAN	Q56UQ5	FLJ44635	TPT1-like protein	2	4.5787	1.5065	-1.604
TBA1C_HUMAN	Q9BQE3	TUBA1C	Tubulin alpha-1C chain	178	378.948	123.798	-1.614
RANG_HUMAN	P43487	RANBP1	Ran-specific GTPase-activating protein	3	1.6539	0.54	-1.615
TBA1A_HUMAN	Q71U36	TUBA1A	Tubulin alpha-1A chain	174	373.191	121.522	-1.619
CD81_HUMAN	P60033	CD81	CD81 antigen	34	28.2569	9.1835	-1.621
CAV1_HUMAN	Q03135	CAV1	Caveolin-1	61	140.691	45.2034	-1.638
ARF3_HUMAN	P61204	ARF3	ADP-ribosylation factor 3	9	4.6011	1.4747	-1.642
PRAF3_HUMAN	O75915	ARL6IP5	PRA1 family protein 3	8	6.9673	2.2282	-1.645
ANXA5_HUMAN	P08758	ANXA5	Annexin A5	166	261.506	82.3805	-1.666
IQGA1_HUMAN	P46940	IQGAP1	Ras GTPase-activating-like protein IQGAP1	38	27.7694	8.636	-1.685
SC31A_HUMAN	O94979	SEC31A	Protein transport protein Sec31A	3	1.2469	0.3866	-1.689
ENOA_HUMAN	P06733	ENO1	Alpha-enolase	44	55.6346	17.2104	-1.693
PLIN3_HUMAN	O60664	PLIN3	Perilipin-3	15	15.973	4.9064	-1.703
FRIL_HUMAN	P02792	FTL	Ferritin light chain	3	1.4835	0.4519	-1.715
AK1A1_HUMAN	P14550	AKR1A1	Alcohol dehydrogenase [NADP+]	3	0.809	0.2387	-1.761
TAGL2_HUMAN	P37802	TAGLN2	Transgelin-2	11	8.5057	2.4824	-1.777
KCD12_HUMAN	Q96CX2	KCTD12	BTB/POZ domain-containing protein KCTD12	5	2.982	0.8536	-1.805
MAVS_HUMAN	Q7Z434	MAVS	Mitochondrial antiviral-signaling protein	4	4.9428	1.4132	-1.806
SYG_HUMAN	P41250	GARS	Glycyl-tRNA synthetase	9	5.6767	1.5828	-1.843
RS5_HUMAN	P46782	RPS5	40S ribosomal protein S5	5	1.3106	0.3573	-1.875
MYL6_HUMAN	P60660	MYL6	Myosin light polypeptide 6	22	33.2056	8.9789	-1.887
K22E_HUMAN	P35908	KRT2	Keratin, type II cytoskeletal 2 epidermal	29	70.6481	18.7788	-1.912
EPCR_HUMAN	Q9UNN8	PROCR	Endothelial protein C receptor	16	32.3128	8.3174	-1.958
CAN1_HUMAN	P07384	CAPN1	Calpain-1 catalytic subunit	2	0.7334	0.1855	-1.983
ALDOA_HUMAN	P04075	ALDOA	Fructose-bisphosphate aldolase A	10	19.9082	4.9174	-2.017
SRC8_HUMAN	Q14247	CTTN	Src substrate cortactin	4	2.8914	0.7131	-2.020
K1C10_HUMAN	P13645	KRT10	Keratin, type I cytoskeletal 10	21	18.051	4.4447	-2.022
CD109_HUMAN	Q6YHK3	CD109	CD109 antigen	10	5.9526	1.4226	-2.065
ENDD1_HUMAN	O94919	ENDOD1	Endonuclease domain-containing 1 protein	2	0.7812	0.1855	-2.074
AT11C_HUMAN	Q8NB49	ATP11C	Probable phospholipid-transporting ATPase IG	4	1.7549	0.4143	-2.083
HSP74_HUMAN	P34932	HSPA4	Heat shock 70 kDa protein 4	2	0.8462	0.1855	-2.190
AHNK2_HUMAN	Q8IVF2	AHNAK2	Protein AHNAK2	27	46.624	10.1623	-2.198
ISLR_HUMAN	O14498	ISLR	Immunoglobulin superfamily containing leucine-rich repeat protein	15	16.7365	3.6167	-2.210
VTA1_HUMAN	Q9NP79	VTA1	Vacuolar protein sorting-associated protein VTA1 homolog	7	3.6976	0.7962	-2.215
M2OM_HUMAN	Q02978	SLC25A11	Mitochondrial 2-oxoglutarate/malate carrier protein	9	5.9052	1.2689	-2.218
MIF_HUMAN	P14174	MIF	Macrophage migration inhibitory factor	3	3.6176	0.7747	-2.223
COF1_HUMAN	P23528	CFL1	Cofilin-1	22	30.611	6.4799	-2.240

G3P_HUMAN	P04406	GAPDH	Glyceraldehyde-3-phosphate dehydrogenase	81	200.066	42.0685	-2.250
IPO9_HUMAN	Q96P70	IPO9	Importin-9	2	0.8861	0.1855	-2.256
TCPB_HUMAN	P78371	CCT2	T-complex protein 1 subunit beta	2	0.8869	0.1855	-2.257
A1L0S7_HUMAN	A1L0S7	TNS1	TNS1 protein (Fragment)	23	30.6895	6.2587	-2.294
K2C1_HUMAN	P04264	KRT1	Keratin, type II cytoskeletal 1	137	227.105	46.2046	-2.297
LPP3_HUMAN	O14495	PPAP2B	Lipid phosphate phosphohydrolase 3	9	11.977	2.3897	-2.325
MRP4_HUMAN	O15439	ABCC4	Multidrug resistance-associated protein 4	4	1.9101	0.3794	-2.332
NEP_HUMAN	P08473	MME	Neprilysin	9	4.0288	0.7977	-2.336
ARC1B_HUMAN	O15143	ARPC1B	Actin-related protein 2/3 complex subunit 1B	3	5.3469	1.0519	-2.346
A6NGN7_HUMAN	A6NGN7	hCG_1787790	Ribosomal protein L1	2	0.9707	0.1855	-2.388
KHDR1_HUMAN	Q07666	KHDRBS1	KH domain-containing, RNA-binding, signal transduction-associated protein 1	2	0.9678	0.1803	-2.424
NB5R1_HUMAN	Q9UHQ9	CYB5R1	NADH-cytochrome b5 reductase 1	6	5.2901	0.9826	-2.429
CLIC1_HUMAN	O00299	CLIC1	Chloride intracellular channel protein 1	2	1.0057	0.1855	-2.439
COPB2_HUMAN	P35606	COPB2	Coatomer subunit beta~	4	2.3193	0.4275	-2.440
TCPQ_HUMAN	P50990	CCT8	T-complex protein 1 subunit theta	6	2.9152	0.5142	-2.503
TIAM1_HUMAN	Q13009	TIAM1	T-lymphoma invasion and metastasis-inducing protein 1	2	2.6927	0.4736	-2.507
FPRP_HUMAN	Q9P2B2	PTGFRN	Prostaglandin F2 receptor negative regulator	3	3.892	0.6764	-2.525
COPA_HUMAN	P53621	COPA	Coatomer subunit alpha	5	9.1969	1.5956	-2.527
MK01_HUMAN	P28482	MAPK1	Mitogen-activated protein kinase 1	2	1.0735	0.1855	-2.533
PSD12_HUMAN	O00232	PSMD12	26S proteasome non-ATPase regulatory subunit 12	2	1.0877	0.1855	-2.552
S39A7_HUMAN	Q92504	SLC39A7	Zinc transporter SLC39A7	3	2.6461	0.4458	-2.569
CPNS1_HUMAN	P04632	CAPNS1	Calpain small subunit 1	2	1.1353	0.1855	-2.614
G6PI_HUMAN	P06744	GPI	Glucose-6-phosphate isomerase	2	1.1559	0.1855	-2.640
K2C5_HUMAN	P13647	KRT5	Keratin, type II cytoskeletal 5	14	12.879	2.0541	-2.648
AGK_HUMAN	Q53H12	AGK	Acylglycerol kinase, mitochondrial	3	2.1045	0.3331	-2.659
KDM5B_HUMAN	Q9UGL1	KDM5B	Lysine-specific demethylase 5B	6	5.5974	0.8851	-2.661
K1C14_HUMAN	P02533	KRT14	Keratin, type I cytoskeletal 14	18	14.8541	2.3484	-2.661
KANK2_HUMAN	Q63ZY3	KANK2	KN motif and ankyrin repeat domain-containing protein 2	4	2.1599	0.3311	-2.706
NXP20_HUMAN	Q8IWE2	FAM114A1	Protein NOXP20	5	1.8772	0.2855	-2.717
TAP1_HUMAN	Q03518	TAP1	Antigen peptide transporter 1	6	5.6897	0.8586	-2.728
TMTC3_HUMAN	Q6ZXV5	TMTC3	Transmembrane and TPR repeat-containing protein 3	5	2.1493	0.3229	-2.735
DCTN2_HUMAN	Q13561	DCTN2	Dynactin subunit 2	2	1.2461	0.1855	-2.748
GFPT2_HUMAN	O94808	GFPT2	Glucosamine--fructose-6-phosphate aminotransferase [isomerizing] 2	4	9.0356	1.3132	-2.783
LDHB_HUMAN	P07195	LDHB	L-lactate dehydrogenase B chain	2	1.3089	0.1855	-2.819
IPO5_HUMAN	O00410	IPO5	Importin-5	2	1.3676	0.1855	-2.882
CAN5_HUMAN	O15484	CAPN5	Calpain-5	5	7.1093	0.9209	-2.949
MK04_HUMAN	P31152	MAPK4	Mitogen-activated protein kinase 4	2	1.4385	0.1855	-2.955
Q6S4P3_HUMAN	Q6S4P3	FTL	Ferritin	2	1.4835	0.1855	-3.000

K1C9_HUMAN	P35527	KRT9	Keratin, type I cytoskeletal 9	56	87.6668	10.8321	-3.017
MDHM_HUMAN	P40926	MDH2	Malate dehydrogenase, mitochondrial	3	1.5649	0.1855	-3.077
GELS_HUMAN	P06396	GSN	Gelsolin	16	20.4121	2.3862	-3.097
Q1WWL2_HUMAN	Q1WWL2	PTGFRN	PTGFRN protein (Fragment)	3	4.3543	0.4963	-3.133
RAI14_HUMAN	Q9P0K7	RAI14	Ankycorbin	2	1.6637	0.1855	-3.165
CAN2_HUMAN	P17655	CAPN2	Calpain-2 catalytic subunit	8	10.66	1.1571	-3.204
K2C6C_HUMAN	P48668	KRT6C	Keratin, type II cytoskeletal 6C	20	19.138	2.0541	-3.220
S27A3_HUMAN	Q5K4L6	SLC27A3	Long-chain fatty acid transport protein 3	2	1.7413	0.1855	-3.231
SPTB2_HUMAN	Q01082	SPTBN1	Spectrin beta chain, brain 1	40	40.9777	4.3575	-3.233
TKT_HUMAN	P29401	TKT	Transketolase	3	1.8033	0.1855	-3.281
ADDG_HUMAN	Q9UEY8	ADD3	Gamma-adducin	3	1.8838	0.1855	-3.344
SPTA2_HUMAN	Q13813	SPTAN1	Spectrin alpha chain, brain	33	30.351	2.6438	-3.521
GDIR1_HUMAN	P52565	ARHGDIA	Rho GDP-dissociation inhibitor 1	3	2.1533	0.1855	-3.537
JAK1_HUMAN	P23458	JAK1	Tyrosine-protein kinase JAK1	2	2.1842	0.1855	-3.558
PLST_HUMAN	P13797	PLS3	Plastin-3	9	7.0087	0.5907	-3.569
KCC2D_HUMAN	Q13557	CAMK2D	Calcium/calmodulin-dependent protein kinase type II delta chain	12	8.9901	0.7541	-3.576
DPYL1_HUMAN	Q14194	CRMP1	Dihydropyrimidinase-related protein 1	2	2.2653	0.1855	-3.610
MAP1B_HUMAN	P46821	MAP1B	Microtubule-associated protein 1B	5	5.898	0.4394	-3.747
ZCH18_HUMAN	Q86VM9	ZC3H18	Zinc finger CCCH domain-containing protein 18	3	2.4902	0.1855	-3.747
LASP1_HUMAN	Q14847	LASP1	LIM and SH3 domain protein 1	2	2.514	0.1855	-3.760
XPO2_HUMAN	P55060	CSE1L	Exportin-2	2	2.6539	0.1855	-3.839
K2C6B_HUMAN	P04259	KRT6B	Keratin, type II cytoskeletal 6B	31	37.0671	2.5147	-3.882
ALBU_HUMAN	P02768	ALB	Serum albumin	3	2.7441	0.1855	-3.887
ACE_HUMAN	P12821	ACE	Angiotensin-converting enzyme	4	3.3978	0.1855	-4.195
DCTN1_HUMAN	Q14203	DCTN1	Dynactin subunit 1	4	3.5643	0.1855	-4.264
K6PP_HUMAN	Q01813	PFKP	6-phosphofructokinase type C	4	3.7429	0.1855	-4.335
G6PD_HUMAN	P11413	G6PD	Glucose-6-phosphate 1-dehydrogenase	7	3.7455	0.1855	-4.336
PGK1_HUMAN	P00558	PGK1	Phosphoglycerate kinase 1	3	3.9124	0.1855	-4.399
KCC2B_HUMAN	Q13554	CAMK2B	Calcium/calmodulin-dependent protein kinase type II beta chain	6	4.0368	0.1855	-4.444
KCC2A_HUMAN	Q9UQM7	CAMK2A	Calcium/calmodulin-dependent protein kinase type II alpha chain	6	4.2679	0.1855	-4.524
CRYAB_HUMAN	P02511	CRYAB	Alpha-crystallin B chain	4	4.427	0.1855	-4.577
SYNE3_HUMAN	Q6ZMZ3	C14orf49	Nesprin-3	5	4.5408	0.1855	-4.613
STAT1_HUMAN	P42224	STAT1	Signal transducer and activator of transcription 1-alpha/beta	9	9.4162	0.3739	-4.654
TM14C_HUMAN	Q9P0S9	TMEM14C	Transmembrane protein 14C	2	4.7269	0.1855	-4.671
CP1B1_HUMAN	Q16678	CYP1B1	Cytochrome P450 1B1	2	4.8257	0.1855	-4.701
LEG3_HUMAN	P17931	LGALS3	Galectin-3	10	12.4085	0.4405	-4.816
DPP4_HUMAN	P27487	DPP4	Dipeptidyl peptidase 4	5	5.2906	0.1855	-4.834
A1L3U3_HUMAN	A1L3U3	ABCA8	ABCA8 protein	9	5.4636	0.1855	-4.880
TPIS_HUMAN	P60174	TPI1	Triosephosphate isomerase	6	5.6852	0.1855	-4.938

AKA12_HUMAN	Q02952	AKAP12	A-kinase anchor protein 12	36	77.1562	2.3825	-5.017
UN84A_HUMAN	O94901	UNC84A	Protein unc-84 homolog A	5	6.062	0.1855	-5.030
TRM2_HUMAN	Q96GJ1	TRMT2B	tRNA (uracil-5-)-methyltransferase homolog	3	62.8691	1.9152	-5.037
HSPB6_HUMAN	O14558	HSPB6	Heat shock protein beta-6	2	6.4047	0.1855	-5.110
K1C16_HUMAN	P08779	KRT16	Keratin, type I cytoskeletal 16	12	11.7718	0.3398	-5.115
UGDH_HUMAN	O60701	UGDH	UDP-glucose 6-dehydrogenase	13	19.8059	0.3799	-5.704

Acc: Uniprot accession; Gene: gene name; spec.#: number of identified spectra

TII_{CON} & $TII_{TGF\text{-}\beta 1}$: total sum of TII identified from control/TGF-β1 group

Log2Ratio: Log2(TIITGF-β1/TIICON)
