# Supplementary material for: Proteomic Identification of ADAM12 as a Regulator for TGF-β1-Induced Differentiation of Human Mesenchymal Stem Cells to Smooth Muscle Cells
Source: PLoS One. 2012 Jul 13;7(7):e40820. doi: 10.1371/journal.pone.0040820 (PMC3396647; doi:10.1371/journal.pone.0040820)
Supplement: Table S2 — Subcellular localization and molecular function of identified proteins. (PDF) [file pone.0040820.s004.pdf]

Table S2: Subcellular localization and molecular function of identified proteins

Uniprot ID	Protein	TM§	Localization*	Molecular function
ADA12_HUMAN	Disintegrin and metalloproteinase domain-containing protein 12	1	PM	hydrolase activity
PLOD2_HUMAN	Procollagen-lysine,2-oxoglutarate 5-dioxygenase 2	0	ER	oxidoreductase activity
PVR_HUMAN	Poliovirus receptor	1	PM	
ALG2_HUMAN	Alpha-1,3-mannosyltransferase ALG2	0		
ACOD_HUMAN	Acyl-CoA desaturase	4	ER,Nuc	oxidoreductase activity
SATT_HUMAN	Neutral amino acid transporter A	9	PM	transporter activity
HYOU1_HUMAN	Hypoxia up-regulated protein 1	1	ER	Chaperone,nucleotide binding
FADS2_HUMAN	Fatty acid desaturase 2	4	PM,ER	transport,metalloprotein,oxidoreductase activity
ATP5I_HUMAN	ATP synthase subunit e, mitochondrial	0	Mito	transporter activity,pyrophosphatase activity,ATPase activity
IDHP_HUMAN	Isocitrate dehydrogenase [NADP], mitochondrial	0	Mito	nucleotide binding,oxidoreductase activity
P4HA2_HUMAN	Prolyl 4-hydroxylase subunit alpha-2	0	ER	oxidoreductase activity
RS15_HUMAN	40S ribosomal protein S15	0	Cyto,Ribo	ribonucleoprotein
LDLR_HUMAN	Low-density lipoprotein receptor	1	PM	transport,lipoprotein,calcium ion binding
B4DXK4_HUMAN	cDNA FLJ50908, highly similar to Homo sapiens keratin protein K6irs (K6IRS2), mRNA	0		
RS15A_HUMAN	40S ribosomal protein S15a	0	Cyto,Ribo	ribonucleoprotein
PRKDC_HUMAN	DNA-dependent protein kinase catalytic subunit	0	Nuc	nucleotide binding
PGH1_HUMAN	Prostaglandin G/H synthase 1	0	PM,ER,Golgi	metalloprotein,oxidoreductase activity
FND3B_HUMAN	Fibronectin type III domain-containing protein 3B	1	ER	
PKD2_HUMAN	Polycystin-2	7	PM	transporter activity,calcium ion binding,receptor binding
SEM7A_HUMAN	Semaphorin-7A	0	PM	lipoprotein
BGH3_HUMAN	Transforming growth factor-beta-induced protein ig-h3	1		receptor binding
SGPL1_HUMAN	Sphingosine-1-phosphate lyase 1	0	ER,Nuc	
S27A4_HUMAN	Long-chain fatty acid transport protein 4	2		transport,nucleotide binding,transporter activity
Q8TA92_HUMAN	Similar to AFG3 ATPase family gene 3-like 2 (Yeast) (Fragment)	2	Mito	nucleotide binding,pyrophosphatase activity
RL29_HUMAN	60S ribosomal protein L29	0	Cyto,Ribo	ribonucleoprotein
ANKL2_HUMAN	Ankyrin repeat and LEM domain-containing protein 2	1		
PVRL2_HUMAN	Poliovirus receptor-related protein 2	1	PM	
TMX1_HUMAN	Thioredoxin-related transmembrane protein 1	3	ER,Nuc	oxidoreductase activity
FKBP9_HUMAN	FK506-binding protein 9	0	ER	calcium ion binding
COMP_HUMAN	Cartilage oligomeric matrix protein	0	Ext.	calcium ion binding
RS27L_HUMAN	40S ribosomal protein S27-like protein	0	Ribo,Nuc	ribonucleoprotein
GT251_HUMAN	Procollagen galactosyltransferase 1	0	ER	
MAN1_HUMAN	Inner nuclear membrane protein Man1	2	Nuc	nucleotide binding

Q9UNR6_HUMAN	Squalene epoxidase	4	ER,Nuc	nucleotide binding,oxidoreductase activity
Q495Q6_HUMAN	HCG2043616	1		
SDC1_HUMAN	Syndecan-1	1	PM	
LAMB1_HUMAN	Laminin subunit beta-1	0	PM	
ST1C3_HUMAN	Sulfotransferase 1C3	0	Cyto	
TENC1_HUMAN	Tensin-like C1 domain-containing phosphatase	0	PM	hydrolase activity
PLOD1_HUMAN	Procollagen-lysine,2-oxoglutarate 5-dioxygenase 1	0	ER	oxidoreductase activity
PTPRM_HUMAN	Receptor-type tyrosine-protein phosphatase mu	1	PM	hydrolase activity
AP2M1_HUMAN	AP-2 complex subunit mu	0	PM,Cyto,Mito	transporter activity
OSBL5_HUMAN	Oxysterol-binding protein-related protein 5	0	Cyto	transport
TNR6_HUMAN	Tumor necrosis factor receptor superfamily member 6	0	PM,Cyto	kinase binding
ERP29_HUMAN	Endoplasmic reticulum protein ERp29	0	ER	
FZD7_HUMAN	Frizzled-7	7	PM	
HNRPC_HUMAN	Heterogeneous nuclear ribonucleoproteins C1/C2	0	Nuc	ribonucleoprotein,nucleotide binding
GPI8_HUMAN	GPI-anchor transamidase	2	ER,Nuc	hydrolase activity
MA1B1_HUMAN	Endoplasmic reticulum mannosyl-oligosaccharide 1,2-alpha-mannosidase	1	ER	calcium ion binding,hydrolase activity
GOSR2_HUMAN	Golgi SNAP receptor complex member 2	1	ER,Golgi,Nuc	transporter activity
VPS45_HUMAN	Vacuolar protein sorting-associated protein 45	0	Golgi	transport
VPP1_HUMAN	V-type proton ATPase 116 kDa subunit a isoform 1	7	PM	transporter activity
MUC18_HUMAN	Cell surface glycoprotein MUC18	1	PM	
SRPR_HUMAN	Signal recognition particle receptor subunit alpha	0	ER,Nuc	GTPase activity,pyrophosphatase activity
OSBL8_HUMAN	Oxysterol-binding protein-related protein 8	1		transport
SPRC_HUMAN	SPARC	0	Ext.	calcium ion binding
MIA3_HUMAN	Melanoma inhibitory activity protein 3	0	ER,Nuc	transport
B2CI53_HUMAN	Solute carrier family 4 sodium bicarbonate cotransporter member 7	11		
COCA1_HUMAN	Collagen alpha-1(XII) chain	0	Ext.	
IMPA3_HUMAN	Inositol monophosphatase 3	1		hydrolase activity
FAT1_HUMAN	Protocadherin Fat 1	1	PM	calcium ion binding
AL1B1_HUMAN	Aldehyde dehydrogenase X, mitochondrial	0	Mito	oxidoreductase activity
HNRPM_HUMAN	Heterogeneous nuclear ribonucleoprotein M	0	PM	ribonucleoprotein,nucleotide binding
BET1L_HUMAN	BET1-like protein	1	Golgi	transport
FINC_HUMAN	Fibronectin	0	PM	
VIGLN_HUMAN	Vigilin	0	Cyto,PM	transport,lipoprotein
DERL1_HUMAN	Derlin-1	5	ER,Nuc	transport,receptor binding
USMG5_HUMAN	Up-regulated during skeletal muscle growth protein 5	1	Mito	
PLCE1_HUMAN	1-phosphatidylinositol-4,5-bisphosphate phosphodiesterase epsilon-1	0	PM,Cyto,Golgi	calcium ion binding,hydrolase activity

SCAM3_HUMAN	Secretory carrier-associated membrane protein 3	4	Golgi	transport
F177A_HUMAN	Protein FAM177A1	0		
TIM50_HUMAN	Mitochondrial import inner membrane translocase subunit TIM50	1	Mito	transport,hydrolase activity
AP2B1_HUMAN	AP-2 complex subunit beta	0	PM,Cyto	transporter activity
CALU_HUMAN	Calumenin	0	ER,Golgi	calcium ion binding
F134C_HUMAN	Protein FAM134C	3		
TSP1_HUMAN	Thrombospondin-1	0	PM	calcium ion binding,receptor binding
IF4A1_HUMAN	Eukaryotic initiation factor 4A-I	0	Cyto	ATPase activity,pyrophosphatase activity
Q9BW34_HUMAN	EEF1D protein (Fragment)	0	Cyto	
UBXN4_HUMAN	UBX domain-containing protein 4	0	ER	
CPT1A_HUMAN	Carnitine O-palmitoyltransferase 1, liver isoform	2	Mito	transport
S38AA_HUMAN	Putative sodium-coupled neutral amino acid transporter 10	9		transport
RL36_HUMAN	60S ribosomal protein L36	0	Cyto,Ribo	ribonucleoprotein
MBOA7_HUMAN	Lysophospholipid acyltransferase 7	5		
RCN3_HUMAN	Reticulocalbin-3	0	ER	calcium ion binding
STX18_HUMAN	Syntaxin-18	1	ER,Golgi	transport
CTNB1_HUMAN	Catenin beta-1	0	PM,Cyto	cytoskeleton,receptor binding,kinase binding
P4HA1_HUMAN	Prolyl 4-hydroxylase subunit alpha-1	0	ER,Mito	metalloprotein,oxidoreductase activity
EGLN_HUMAN	Endoglin	1	PM	receptor binding
A0AV88_HUMAN	ADAM10 protein	0		
DYL1_HUMAN	Dynein light chain 1, cytoplasmic	0	PM,Cyto	pyrophosphatase activity
K220L_HUMAN	Putative NPIP-like protein KIAA0220-like	0		
CHSTE_HUMAN	Carbohydrate sulfotransferase 14	0	Golgi	
PTK7_HUMAN	Tyrosine-protein kinase-like 7	2	PM	nucleotide binding
FIS1_HUMAN	Mitochondrial fission 1 protein	1	Mito	
4F2_HUMAN	4F2 cell-surface antigen heavy chain	1	PM	transporter activity
SSRB_HUMAN	Translocon-associated protein subunit beta	1	ER,Nuc	peptide binding
RB22A_HUMAN	Ras-related protein Rab-22A	0	PM	transport,lipoprotein,GTPase activity,pyrophosphatase activity
KDIS_HUMAN	Kinase D-interacting substrate of 220 kDa	4	Cyto	
STX2_HUMAN	Syntaxin-2	1	PM	
CLPT1_HUMAN	Cleft lip and palate transmembrane protein 1	5	PM	
UGPA_HUMAN	UTP--glucose-1-phosphate uridylyltransferase	0	Cyto	
K0776_HUMAN	UPF0555 protein KIAA0776	0		
SHPS1_HUMAN	Tyrosine-protein phosphatase non-receptor type substrate 1	1	PM	
RL19_HUMAN	60S ribosomal protein L19	0	Cyto,Ribo	ribonucleoprotein

S12A4_HUMAN	Solute carrier family 12 member 4	12	PM	transporter activity
SC65_HUMAN	Synaptonemal complex protein SC65	0	Nuc	
PXDN_HUMAN	Peroxidasin homolog	1		calcium ion binding,oxidoreductase activity,receptor binding
K0090_HUMAN	Uncharacterized protein KIAA0090	0		
APOL2_HUMAN	Apolipoprotein L2	3	ER,Nuc,Cyto	transport,lipoprotein,receptor binding
TEN3_HUMAN	Teneurin-3	1		
SEC63_HUMAN	Translocation protein SEC63 homolog	3	ER	transport,Chaperone
CCD47_HUMAN	Coiled-coil domain-containing protein 47	0	ER	calcium ion binding
RL27A_HUMAN	60S ribosomal protein L27a	0	Cyto,Ribo	ribonucleoprotein
CG059_HUMAN	UPF0539 protein C7orf59	0		
RAB31_HUMAN	Ras-related protein Rab-31	0	PM	lipoprotein,GTPase activity,pyrophosphatase activity
GOSR1_HUMAN	Golgi SNAP receptor complex member 1	1	Golgi	transport
LIPB1_HUMAN	Liprin-beta-1	0	PM	
DERL2_HUMAN	Derlin-2	4	ER,Nuc	
AP2A2_HUMAN	AP-2 complex subunit alpha-2	0	PM,Cyto	transporter activity
DYSF_HUMAN	Dysferlin	1	PM	
GALT2_HUMAN	Polypeptide N-acetylgalactosaminyltransferase 2	1	Golgi	calcium ion binding
TPM1_HUMAN	Tropomyosin alpha-1 chain	0	Cyto,PM	cytoskeleton
ITA1_HUMAN	Integrin alpha-1	1	PM	integrin,calcium ion binding,receptor binding
MFGM_HUMAN	Lactadherin	0	PM	receptor binding
ACSL3_HUMAN	Long-chain-fatty-acid--CoA ligase 3	1	ER,Mito	nucleotide binding
AAAT_HUMAN	Neutral amino acid transporter B(0)	9	PM	transporter activity
PICAL_HUMAN	Phosphatidylinositol-binding clathrin assembly protein	0	PM,Golgi	
BT2A1_HUMAN	Butyrophilin subfamily 2 member A1	2	PM	
NCPR_HUMAN	NADPH--cytochrome P450 reductase	1	ER	nucleotide binding,oxidoreductase activity
YIPF5_HUMAN	Protein YIPF5	4	ER,Golgi	transport
LIMA1_HUMAN	LIM domain and actin-binding protein 1	0	Cyto,PM	cytoskeleton
AP2A1_HUMAN	AP-2 complex subunit alpha-1	0	PM,Cyto,Golgi	transporter activity
INF2_HUMAN	Inverted formin-2	0		
MICA2_HUMAN	Protein MICAL-2	0	Cyto	cytoskeleton,oxidoreductase activity
RDH11_HUMAN	Retinol dehydrogenase 11	0	ER	oxidoreductase activity
GBG5_HUMAN	Guanine nucleotide-binding protein G(I)/G(S)/G(O) subunit gamma-5	0	PM	lipoprotein,pyrophosphatase activity,GTPase activity
B2MG_HUMAN	Beta-2-microglobulin	0	PM,Golgi	
SAR1B_HUMAN	GTP-binding protein SAR1b	0	ER,Golgi	transport,nucleotide binding
LGAT1_HUMAN	Acyl-CoA:lysophosphatidylglycerol acyltransferase 1	4	ER	
FA38A_HUMAN	Protein FAM38A	23	ER	
PI16_HUMAN	Peptidase inhibitor 16	1		hydrolase activity

MA2A1_HUMAN	Alpha-mannosidase 2	1	Golgi	hydrolase activity
NCLN_HUMAN	Nicalin	2	ER	
IF2A_HUMAN	Eukaryotic translation initiation factor 2 subunit 1	0	Cyto	
SC61B_HUMAN	Protein transport protein Sec61 subunit beta	1	ER,Nuc	transporter activity
DHCR7_HUMAN	7-dehydrocholesterol reductase	6	ER,Nuc	oxidoreductase activity
KTN1_HUMAN	Kinectin	1	PM,ER,Nuc	
VANG1_HUMAN	Vang-like protein 1	4		
RRBP1_HUMAN	Ribosome-binding protein 1	1	ER,Ribo,Nuc	transport
CD99_HUMAN	CD99 antigen	2	PM	
TM9S4_HUMAN	Transmembrane 9 superfamily member 4	10		
GL8D1_HUMAN	Glycosyltransferase 8 domain-containing protein 1	1	Mito	
SPTC1_HUMAN	Serine palmitoyltransferase 1	2	ER,Nuc	
B2R701_HUMAN	cDNA, FLJ93202, Homo sapiens protease inhibitor 16 (PI16), mRNA	0		
FAF2_HUMAN	FAS-associated factor 2	0	Cyto	
MPRI_HUMAN	Cation-independent mannose-6-phosphate receptor	1	PM,Golgi	transporter activity
ITA11_HUMAN	Integrin alpha-11	1	PM	integrin,calcium ion binding
FDFT_HUMAN	Squalene synthetase	0	ER,Nuc	oxidoreductase activity
PDIA4_HUMAN	Protein disulfide-isomerase A4	1	ER	calcium ion binding
CO1A2_HUMAN	Collagen alpha-2(I) chain	0	PM	
ITFG3_HUMAN	Protein ITFG3	1		
TM165_HUMAN	Transmembrane protein 165	7		
SSRA_HUMAN	Translocon-associated protein subunit alpha	1	ER,Nuc	calcium ion binding,peptide binding
A7XZE4_HUMAN	Beta tropomyosin isoform	0		
ADT1_HUMAN	ADP/ATP translocase 1	3	PM,Mito	transporter activity
CTND1_HUMAN	Catenin delta-1	0	Cyto,PM	
PTH2_HUMAN	Peptidyl-tRNA hydrolase 2, mitochondrial	1	Mito	hydrolase activity
CO024_HUMAN	UPF0480 protein C15orf24	1		nucleotide binding
AT1B1_HUMAN	Sodium/potassium-transporting ATPase subunit beta-1	1	PM	transporter activity,pyrophosphatase activity,ATPase activity
EPHB3_HUMAN	Ephrin type-B receptor 3	1	PM	nucleotide binding
NUCB2_HUMAN	Nucleobindin-2	1	PM,ER,Cyto,Golgi,Nuc	calcium ion binding
RL32_HUMAN	60S ribosomal protein L32	0	Cyto,Ribo	ribonucleoprotein
ERP44_HUMAN	Endoplasmic reticulum resident protein ERp44	0	ER,Nuc	Chaperone
CSPG4_HUMAN	Chondroitin sulfate proteoglycan 4	1	PM	
STX12_HUMAN	Syntaxin-12	1	Golgi	transport
AT2A2_HUMAN	Sarcoplasmic/endoplasmic reticulum calcium ATPase 2	8	PM,ER,Nuc	ATPase activity,transporter activity,pyrophosphatase activity,calcium ion binding,receptor binding

COMT_HUMAN	Catechol O-methyltransferase	1	PM,Cyto	lipoprotein
SRPRB_HUMAN	Signal recognition particle receptor subunit beta	1	ER	ribonucleoprotein,nucleotide binding
CTL2_HUMAN	Choline transporter-like protein 2	11		transporter activity
NUCB1_HUMAN	Nucleobindin-1	0	Cyto,Golgi	calcium ion binding
RENR_HUMAN	Renin receptor	1	PM	
Q5TCU3_HUMAN	Tropomyosin 2 (Beta)	0	Cyto	cytoskeleton
NPM_HUMAN	Nucleophosmin	0	Cyto,Ribo	Chaperone
SL9A1_HUMAN	Sodium/hydrogen exchanger 1	12	PM	transporter activity
LMF2_HUMAN	Lipase maturation factor 2	7	ER	
H2AV_HUMAN	Histone H2A.V	0	Nuc	
NICA_HUMAN	Nicastrin	1	PM,ER,Golgi	hydrolase activity
H2AJ_HUMAN	Histone H2A.J	0	Nuc	
EPHA7_HUMAN	Ephrin type-A receptor 7	1	PM	nucleotide binding,receptor binding
RL27_HUMAN	60S ribosomal protein L27	0	Cyto,Ribo	ribonucleoprotein
SURF4_HUMAN	Surfeit locus protein 4	5	ER	
CADH2_HUMAN	Cadherin-2	1	PM	calcium ion binding,kinase binding
RAB21_HUMAN	Ras-related protein Rab-21	0	ER,Golgi	transport,lipoprotein,nucleotide binding
STX7_HUMAN	Syntaxin-7	1	Golgi	
P3H3_HUMAN	Prolyl 3-hydroxylase 3	0	ER	oxidoreductase activity
NOMO1_HUMAN	Nodal modulator 1	0	ER,Nuc	
RAB6A_HUMAN	Ras-related protein Rab-6A	0	Golgi	transport,lipoprotein,GTPase activity,pyrophosphatase activity
H2BFS_HUMAN	Histone H2B type F-S	0	Nuc	
SSRD_HUMAN	Translocon-associated protein subunit delta	0	ER,Nuc	calcium ion binding
H2A2A_HUMAN	Histone H2A type 2-A	0	Nuc	
ERG25_HUMAN	C-4 methylsterol oxidase	3	PM,ER,Nuc	oxidoreductase activity
LPLC3_HUMAN	Long palate, lung and nasal epithelium carcinoma-associated protein 3	0	Cyto	
RL12_HUMAN	60S ribosomal protein L12	0	Cyto,Ribo	ribonucleoprotein
TOR1A_HUMAN	Torsin-1A	0	ER,Nuc	Chaperone,nucleotide binding,hydrolase activity
RL15_HUMAN	60S ribosomal protein L15	0	Cyto,Ribo	ribonucleoprotein
FKB10_HUMAN	FK506-binding protein 10	0	ER	calcium ion binding
PARVA_HUMAN	Alpha-parvin	0	Cyto,PM	cytoskeleton
TAGL_HUMAN	Transgelin	0	Cyto	
HBXIP_HUMAN	Hepatitis B virus X-interacting protein	0	Cyto	
Q5HYB6_HUMAN	Putative uncharacterized protein DKFZp686J1372	0	Cyto	cytoskeleton
NCEH1_HUMAN	Neutral cholesterol ester hydrolase 1	1	ER	hydrolase activity
TMEM2_HUMAN	Transmembrane protein 2	1		
ADT2_HUMAN	ADP/ATP translocase 2	2	PM,Mito	transporter activity

RAN_HUMAN	GTP-binding nuclear protein Ran	0	Cyto	transport,GTPase activity,pyrophosphatase activity,receptor binding
SMC3_HUMAN	Structural maintenance of chromosomes protein 3	0	Ext.,Nuc	nucleotide binding,pyrophosphatase activity
STT3A_HUMAN	Dolichyl-diphosphooligosaccharide--protein glycosyltransferase subunit STT3A	12	ER,Nuc	
MOS_HUMAN	Proto-oncogene serine/threonine-protein kinase mos	0		nucleotide binding
NDUS2_HUMAN	NADH dehydrogenase [ubiquinone] iron-sulfur protein 2, mitochondrial	0	Mito	transport,nucleotide binding,oxidoreductase activity
HM13_HUMAN	Minor histocompatibility antigen H13	7	ER	hydrolase activity
RL8_HUMAN	60S ribosomal protein L8	0	Cyto,Ribo	ribonucleoprotein
CCD19_HUMAN	Coiled-coil domain-containing protein 19, mitochondrial	0	Mito	
RL13_HUMAN	60S ribosomal protein L13	0	Cyto,Ribo	ribonucleoprotein
LYRIC_HUMAN	Protein LYRIC	1	PM,ER,Nuc,Cyto	
YIF1B_HUMAN	Protein YIF1B	5		
RS13_HUMAN	40S ribosomal protein S13	0	Cyto,Ribo	ribonucleoprotein
TBL2_HUMAN	Transducin beta-like protein 2	1		
MXRA7_HUMAN	Matrix-remodeling-associated protein 7	1		
MOGS_HUMAN	Mannosyl-oligosaccharide glucosidase	1	ER	hydrolase activity
ADT3_HUMAN	ADP/ATP translocase 3	2	Mito	transporter activity
RAC1_HUMAN	Ras-related C3 botulinum toxin substrate 1	0	PM,Cyto	lipoprotein,GTPase activity,pyrophosphatase activity
Q562Z4_HUMAN	Actin-like protein (Fragment)	0	Cyto	cytoskeleton,nucleotide binding,kinase binding
GBLP_HUMAN	Guanine nucleotide-binding protein subunit beta-2-like 1	0	PM	receptor binding
PGAM1_HUMAN	Phosphoglycerate mutase 1	0	Cyto	kinase binding,hydrolase activity
GOLI4_HUMAN	Golgi integral membrane protein 4	1	Golgi	transport
UTRO_HUMAN	Utrophin	0	Cyto,PM	cytoskeleton,calcium ion binding
A5YM53_HUMAN	ITGAV protein	1		
DRS7B_HUMAN	Dehydrogenase/reductase SDR family member 7B	1		oxidoreductase activity
CALR_HUMAN	Calreticulin	0	PM,ER,Cyto	Chaperone,calcium ion binding,receptor binding
SSRG_HUMAN	Translocon-associated protein subunit gamma	4	ER,Nuc	peptide binding
CD47_HUMAN	Leukocyte surface antigen CD47	6	PM	integrin
RAP1A_HUMAN	Ras-related protein Rap-1A	0	PM,Cyto	lipoprotein,GTPase activity,pyrophosphatase activity
CKAP4_HUMAN	Cytoskeleton-associated protein 4	1	ER	lipoprotein
SERPH_HUMAN	Serpin H1	0	ER	Chaperone
DMD_HUMAN	Dystrophin	0	Cyto,PM	cytoskeleton,calcium ion binding
CALX_HUMAN	Calnexin	1	ER	Chaperone,calcium ion binding
ITB1_HUMAN	Integrin beta-1	1	PM	integrin,receptor binding
TXTP_HUMAN	Tricarboxylate transport protein, mitochondrial	0	Mito	transporter activity
SAC1_HUMAN	Phosphatidylinositide phosphatase SAC1	2	ER,Golgi,Nuc	hydrolase activity

ARL1_HUMAN	ADP-ribosylation factor-like protein 1	0	PM,Golgi	lipoprotein,GTPase activity,pyrophosphatase activity
CXA1_HUMAN	Gap junction alpha-1 protein	4	PM,ER,Mito,Golgi	transporter activity
HNRPU_HUMAN	Heterogeneous nuclear ribonucleoprotein U	0	Nuc	ribonucleoprotein,nucleotide binding
ELOV1_HUMAN	Elongation of very long chain fatty acids protein 1	7	ER	
COX5B_HUMAN	Cytochrome c oxidase subunit 5B, mitochondrial	0	Mito	transporter activity,oxidoreductase activity
ERD22_HUMAN	ER lumen protein retaining receptor 2	4	ER,Golgi	transport,peptide binding
LAMP1_HUMAN	Lysosome-associated membrane glycoprotein 1	2	PM	
ENPL_HUMAN	Endoplasmin	0	ER,Cyto,Nuc	Chaperone,nucleotide binding,calcium ion binding,receptor binding
RSSA_HUMAN	40S ribosomal protein SA	0	PM,Cyto,Ribo	ribonucleoprotein
ARF4_HUMAN	ADP-ribosylation factor 4	0	PM,Golgi	transport,lipoprotein,GTPase activity,pyrophosphatase activity,receptor binding
RAB8B_HUMAN	Ras-related protein Rab-8B	0	PM	transport,lipoprotein,GTPase activity,pyrophosphatase activity
PIGS_HUMAN	GPI transamidase component PIG-S	2	ER,Nuc	hydrolase activity
ZDHC5_HUMAN	Probable palmitoyltransferase ZDHHC5	4		
RL18A_HUMAN	60S ribosomal protein L18a	0	Cyto,Ribo	ribonucleoprotein
RAP1B_HUMAN	Ras-related protein Rap-1b	0	PM,Cyto	lipoprotein,GTPase activity,pyrophosphatase activity
CTNA1_HUMAN	Catenin alpha-1	0	Cyto,PM	cytoskeleton
Q58F09_HUMAN	Glucosidase I	1	ER	hydrolase activity
TM9S2_HUMAN	Transmembrane 9 superfamily member 2	9	PM	
UN84B_HUMAN	Protein unc-84 homolog B	1	Nuc	
Q3B7K3_HUMAN	IKIP protein (Fragment)	0	ER	
TMM33_HUMAN	Transmembrane protein 33	3		
TOM22_HUMAN	Mitochondrial import receptor subunit TOM22 homolog	1	Mito	transporter activity
GNAS1_HUMAN	Guanine nucleotide-binding protein G(s) subunit alpha isoforms XLas	0	PM,Golgi	lipoprotein,GTPase activity,pyrophosphatase activity,receptor binding
GSLG1_HUMAN	Golgi apparatus protein 1	1	Golgi	receptor binding
AL2S4_HUMAN	Amyotrophic lateral sclerosis 2 chromosomal region candidate gene 4 protein	4		
TMED4_HUMAN	Transmembrane emp24 domain-containing protein 4	2	ER	
TXND5_HUMAN	Thioredoxin domain-containing protein 5	0	ER	
TMM85_HUMAN	Transmembrane protein 85	2		
CSRP1_HUMAN	Cysteine and glycine-rich protein 1	0	Nuc	
CD166_HUMAN	CD166 antigen	1	PM	receptor binding
PHB2_HUMAN	Prohibitin-2	0	Cyto,Mito	receptor binding
TUSC3_HUMAN	Tumor suppressor candidate 3	5	ER,Mito,Nuc	
ITB5_HUMAN	Integrin beta-5	1	PM	cytoskeleton,integrin

RPN1_HUMAN	Dolichyl-diphosphooligosaccharide--protein glycosyltransferase subunit 1	1	ER,Nuc	
TMX3_HUMAN	Protein disulfide-isomerase TMX3	1	ER	
TOM70_HUMAN	Mitochondrial import receptor subunit TOM70	2	Mito	transporter activity
RAB35_HUMAN	Ras-related protein Rab-35	0	PM	transport,lipoprotein,GTPase activity,pyrophosphatase activity
RAB14_HUMAN	Ras-related protein Rab-14	0	PM,ER,Cyto,Golgi,Nuc	transport,lipoprotein,GTPase activity,pyrophosphatase activity
TPM4_HUMAN	Tropomyosin alpha-4 chain	0	Cyto	cytoskeleton,calcium ion binding
IKIP_HUMAN	Inhibitor of nuclear factor kappa-B kinase-interacting protein	0	ER	
DREB_HUMAN	Drebrin	0	Cyto	
RAB2A_HUMAN	Ras-related protein Rab-2A	0	ER,Golgi	transport,lipoprotein,GTPase activity,pyrophosphatase activity
VKGC_HUMAN	Vitamin K-dependent gamma-carboxylase	5	ER	
RS23_HUMAN	40S ribosomal protein S23	0	Cyto,Ribo	ribonucleoprotein
ALG5_HUMAN	Dolichyl-phosphate beta-glucosyltransferase	1	ER	
ADPGK_HUMAN	ADP-dependent glucokinase	0		
MGST3_HUMAN	Microsomal glutathione S-transferase 3	4	ER	oxidoreductase activity
EF1G_HUMAN	Elongation factor 1-gamma	0	Cyto	
AIFM1_HUMAN	Apoptosis-inducing factor 1, mitochondrial	0	ER,Mito	nucleotide binding,oxidoreductase activity
ZW10_HUMAN	Centromere/kinetochore protein zw10 homolog	0	ER,Cyto	transport
BST1_HUMAN	ADP-ribosyl cyclase 2	1	PM	lipoprotein,hydrolase activity
CHP1_HUMAN	Calcium-binding protein p22	0	Cyto	lipoprotein,calcium ion binding
GAPR1_HUMAN	Golgi-associated plant pathogenesis-related protein 1	0	Golgi	lipoprotein
GOGA5_HUMAN	Golgin subfamily A member 5	1	Golgi	nucleotide binding
GRP78_HUMAN	78 kDa glucose-regulated protein	0	ER,Cyto,Nuc	nucleotide binding,calcium ion binding
PPAC2_HUMAN	Presqualene diphosphate phosphatase	3		hydrolase activity
ADA17_HUMAN	Disintegrin and metalloproteinase domain-containing protein 17	1	PM	receptor binding,hydrolase activity
RAB8A_HUMAN	Ras-related protein Rab-8A	0	PM	transport,lipoprotein,nucleotide binding
MYOF_HUMAN	Myoferlin	1	PM	
GALT1_HUMAN	Polypeptide N-acetylgalactosaminyltransferase 1	1	Golgi	calcium ion binding
EBP_HUMAN	3-beta-hydroxysteroid-Delta(8),Delta(7)-isomerase	5	PM,ER	transporter activity
CTGE5_HUMAN	Cutaneous T-cell lymphoma-associated antigen 5	1		
COX41_HUMAN	Cytochrome c oxidase subunit 4 isoform 1, mitochondrial	1	Mito	transporter activity,oxidoreductase activity
LAMP2_HUMAN	Lysosome-associated membrane glycoprotein 2	1	PM	
RAB10_HUMAN	Ras-related protein Rab-10	0	PM,Golgi	transport,lipoprotein,nucleotide binding

ENPP2_HUMAN	Ectonucleotide pyrophosphatase/phosphodiesterase family member 2	1	PM	pyrophosphatase activity
NSDHL_HUMAN	Sterol-4-alpha-carboxylate 3-dehydrogenase, decarboxylating	1	ER,Nuc	oxidoreductase activity
ATPK_HUMAN	ATP synthase subunit f, mitochondrial	1	Mito	transport,pyrophosphatase activity,ATPase activity
FLOT1_HUMAN	Flotillin-1	0	PM,Golgi	
CLH1_HUMAN	Clathrin heavy chain 1	0	PM,Mito,Golgi	
TFR1_HUMAN	Transferrin receptor protein 1	1	PM,Mito	lipoprotein
KIRR1_HUMAN	Kin of IRRE-like protein 1	1	PM	
EFTU_HUMAN	Elongation factor Tu, mitochondrial	0	Mito	GTPase activity,pyrophosphatase activity
RB11B_HUMAN	Ras-related protein Rab-11B	0	PM	transport,lipoprotein,GTPase activity,pyrophosphatase activity
DNJC1_HUMAN	DnaJ homolog subfamily C member 1	1	ER	Chaperone
LRC59_HUMAN	Leucine-rich repeat-containing protein 59	1	ER,Mito	
TOM40_HUMAN	Mitochondrial import receptor subunit TOM40 homolog	0	Mito	transporter activity
TM214_HUMAN	Transmembrane protein 214	0		
RRAS2_HUMAN	Ras-related protein R-Ras2	0	PM,ER	lipoprotein,GTPase activity,pyrophosphatase activity
ESYT1_HUMAN	Extended synaptotagmin-1	2		
SCFD1_HUMAN	Sec1 family domain-containing protein 1	0	PM,ER,Golgi,Cyto	transport
CAND1_HUMAN	Cullin-associated NEDD8-dissociated protein 1	0	Nuc	
MYO1C_HUMAN	Myosin-Ic	0	Cyto,PM	transport,nucleotide binding,pyrophosphatase activity
RB11A_HUMAN	Ras-related protein Rab-11A	0	PM,Golgi	transport,lipoprotein,GTPase activity,transporter activity,pyrophosphatase activity
PDIA1_HUMAN	Protein disulfide-isomerase	0	PM,ER	Chaperone,oxidoreductase activity
PIGT_HUMAN	GPI transamidase component PIG-T	1	ER,Nuc	hydrolase activity
UCRI_HUMAN	Cytochrome b-c1 complex subunit Rieske, mitochondrial	0	Mito	transporter activity,oxidoreductase activity
UCRI_HUMAN	Cytochrome b-c1 complex subunit Rieske, mitochondrial	0	Mito	transporter activity,oxidoreductase activity
LETM1_HUMAN	LETM1 and EF-hand domain-containing protein 1, mitochondrial	1	Mito	calcium ion binding
A4D1N4_HUMAN	Coiled-coil-helix-coiled-coil-helix domain containing 3, isoform CRA_d	0	Mito	
GNA13_HUMAN	Guanine nucleotide-binding protein subunit alpha-13	0	PM	lipoprotein,GTPase activity,pyrophosphatase activity,receptor binding
STML2_HUMAN	Stomatin-like protein 2	0	Cyto,Mito	cytoskeleton,receptor binding
RAB1B_HUMAN	Ras-related protein Rab-1B	0	PM,Cyto,Golgi	transport,lipoprotein,nucleotide binding
RLA0_HUMAN	60S acidic ribosomal protein P0	0	Cyto,Ribo	ribonucleoprotein
C9JSZ5_HUMAN	Putative uncharacterized protein DDRGK1	#N/A		

PDIA3_HUMAN	Protein disulfide-isomerase A3	0	ER	hydrolase activity
ESYT2_HUMAN	Extended synaptotagmin-2	1	PM	
Q5SV24_HUMAN	ATPase family, AAA domain containing 3A (Fragment)	0		nucleotide binding,pyrophosphatase activity
B8ZZQ7_HUMAN	Putative uncharacterized protein IMMT	0		
PROF1_HUMAN	Profilin-1	0	Cyto	cytoskeleton
SCMC1_HUMAN	Calcium-binding mitochondrial carrier protein SCaMC-1	0	Mito	transport,calcium ion binding
F176B_HUMAN	Protein FAM176B	1		
SC22B_HUMAN	Vesicle-trafficking protein SEC22b	1	ER,Golgi	transport
DAD1_HUMAN	Dolichyl-diphosphooligosaccharide--protein glycosyltransferase subunit DAD1	3	ER,Nuc	
CK059_HUMAN	RhoA activator C11orf59	0	PM	
PALM_HUMAN	Paralemmin	0	PM	lipoprotein,receptor binding
RASH_HUMAN	GTPase HRas	0	PM,Golgi	lipoprotein,GTPase activity,pyrophosphatase activity
ERG7_HUMAN	Lanosterol synthase	0		
TTC35_HUMAN	Tetratricopeptide repeat protein 35	0	ER	
RS18_HUMAN	40S ribosomal protein S18	0	Cyto,Ribo	ribonucleoprotein
DHC24_HUMAN	24-dehydrocholesterol reductase	2	ER,Golgi,Nuc	nucleotide binding,oxidoreductase activity,peptide binding
PCAT1_HUMAN	Lysophosphatidylcholine acyltransferase 1	1	ER,Golgi	calcium ion binding
DHB12_HUMAN	Estradiol 17-beta-dehydrogenase 12	3	ER	oxidoreductase activity
RS17_HUMAN	40S ribosomal protein S17	0	Cyto,Ribo	ribonucleoprotein
PRAF2_HUMAN	PRA1 family protein 2	4		transport
Q3B7A4_HUMAN	RPLP0 protein	0	Cyto,Ribo	ribonucleoprotein
TPBG_HUMAN	Trophoblast glycoprotein	1	PM,ER	
MAGT1_HUMAN	Magnesium transporter protein 1	5	ER,Nuc	
VAPB_HUMAN	Vesicle-associated membrane protein-associated protein B/C	1	PM,ER,Golgi,Nuc	
RAB7A_HUMAN	Ras-related protein Rab-7a	0	Golgi	transport,lipoprotein,GTPase activity,pyrophosphatase activity
FKB11_HUMAN	FK506-binding protein 11	1		
Q8TEP9_HUMAN	FLJ00144 protein (Fragment)	0	ER,Golgi	
NSF_HUMAN	Vesicle-fusing ATPase	0	Cyto	transport,ATPase activity,pyrophosphatase activity
PDIA6_HUMAN	Protein disulfide-isomerase A6	0	ER	
LRP1_HUMAN	Prolow-density lipoprotein receptor-related protein 1	1	PM,Cyto	lipoprotein,transporter activity,calcium ion binding,receptor binding
CATB_HUMAN	Cathepsin B	0	PM,Mito	hydrolase activity,peptide binding
BET1_HUMAN	BET1 homolog	1	ER,Golgi	transport
TMED9_HUMAN	Transmembrane emp24 domain-containing protein 9	2	ER	
PTN1_HUMAN	Tyrosine-protein phosphatase non-receptor type 1	1	PM,ER,Cyto	receptor binding,hydrolase activity

PDCD6_HUMAN	Programmed cell death protein 6	0	ER,Cyto	calcium ion binding
RL3L_HUMAN	60S ribosomal protein L3-like	0	Cyto,Ribo	ribonucleoprotein
PGRC2_HUMAN	Membrane-associated progesterone receptor component 2	1		
TX1B3_HUMAN	Tax1-binding protein 3	0	Cyto	
RHOG_HUMAN	Rho-related GTP-binding protein RhoG	0	PM	lipoprotein,GTPase activity,pyrophosphatase activity
1433B_HUMAN	14-3-3 protein beta/alpha	0	Cyto	oxidoreductase activity
TECR_HUMAN	Trans-2,3-enoyl-CoA reductase	4	ER	oxidoreductase activity
MFS10_HUMAN	Major facilitator superfamily domain-containing protein 10	10		transporter activity
SC11A_HUMAN	Signal peptidase complex catalytic subunit SEC11A	1	ER,Nuc	hydrolase activity
ATLA3_HUMAN	Atlastin-3	2	ER	GTPase activity,pyrophosphatase activity
C7FDR3_HUMAN	MHC class I antigen (Fragment)	0		
MPKS1_HUMAN	Mitogen-activated protein kinase scaffold protein 1	0	PM	
USO1_HUMAN	General vesicular transport factor p115	0	Cyto,Golgi	transporter activity
SYFA_HUMAN	Phenylalanyl-tRNA synthetase alpha chain	0	Cyto	nucleotide binding
K1C18_HUMAN	Keratin, type I cytoskeletal 18	0	Cyto	
S61A1_HUMAN	Protein transport protein Sec61 subunit alpha isoform 1	10	ER,Nuc	transporter activity
CNN2_HUMAN	Calponin-2	0	PM	
RETST_HUMAN	All-trans-retinol 13,14-reductase	1	ER,Nuc	oxidoreductase activity
CD63_HUMAN	CD63 antigen	4	PM	
RL23A_HUMAN	60S ribosomal protein L23a	0	Cyto,Ribo	ribonucleoprotein,nucleotide binding
PTAD1_HUMAN	Protein tyrosine phosphatase-like protein PTPLAD1	5		
RS3_HUMAN	40S ribosomal protein S3	0	PM,Cyto,Ribo	ribonucleoprotein,kinase binding,hydrolase activity
IMB1_HUMAN	Importin subunit beta-1	0	Cyto	transporter activity,peptide binding
IMMT_HUMAN	Mitochondrial inner membrane protein	0	Mito	
QCR6_HUMAN	Cytochrome b-c1 complex subunit 6, mitochondrial	0	Mito	transporter activity,oxidoreductase activity
CO6A3_HUMAN	Collagen alpha-3(VI) chain	0	PM	
NDUV1_HUMAN	NADH dehydrogenase [ubiquinone] flavoprotein 1, mitochondrial	0	Mito	transport,metalloprotein,nucleotide binding,oxidoreductase activity
GNAI3_HUMAN	Guanine nucleotide-binding protein G(k) subunit alpha	0	PM,Golgi	lipoprotein,GTPase activity,pyrophosphatase activity,receptor binding
PRDX6_HUMAN	Peroxiredoxin-6	0	Cyto	oxidoreductase activity
GANAB_HUMAN	Neutral alpha-glucosidase AB	1	ER,Golgi	transporter activity,hydrolase activity
RRAS_HUMAN	Ras-related protein R-Ras	0	PM	lipoprotein,GTPase activity,pyrophosphatase activity
CA2D1_HUMAN	Voltage-dependent calcium channel subunit alpha-2/delta-1	0	PM,ER	transporter activity,calcium ion binding
SPCS2_HUMAN	Signal peptidase complex subunit 2	2	ER,Nuc	hydrolase activity

TMED7_HUMAN	Transmembrane emp24 domain-containing protein 7	1	PM,ER,Golgi,Cyto	lipoprotein
HXK1_HUMAN	Hexokinase-1	0	Cyto,Mito	nucleotide binding
DEST_HUMAN	Destrin	0		
ARF6_HUMAN	ADP-ribosylation factor 6	0	PM,Golgi	transport,lipoprotein,GTPase activity,pyrophosphatase activity
Q53TP5_HUMAN	Fibroblast activation protein, alpha, isoform CRA_a	1	PM	hydrolase activity
P3H1_HUMAN	Prolyl 3-hydroxylase 1	0	ER	oxidoreductase activity
DGAT1_HUMAN	Diacylglycerol O-acyltransferase 1	9	ER,Cyto	
PRDX4_HUMAN	Peroxiredoxin-4	0	Cyto,Mito	oxidoreductase activity
GPC6_HUMAN	Glypican-6	0	PM	lipoprotein
STT3B_HUMAN	Dolichyl-diphosphooligosaccharide--protein glycosyltransferase subunit STT3B	10	ER,Nuc	
NB5R3_HUMAN	NADH-cytochrome b5 reductase 3	0	PM,ER,Cyto,Mito,Nuc	lipoprotein,oxidoreductase activity
NDUA8_HUMAN	NADH dehydrogenase [ubiquinone] 1 alpha subcomplex subunit 8	0	Mito	transport,oxidoreductase activity
FACE1_HUMAN	CAAX prenyl protease 1 homolog	7	ER,Golgi	hydrolase activity
EFR3A_HUMAN	Protein EFR3 homolog A	0	PM	
RS2_HUMAN	40S ribosomal protein S2	0	Cyto,Ribo	ribonucleoprotein
IBP5_HUMAN	Insulin-like growth factor-binding protein 5	0	Ext.	
KAP0_HUMAN	cAMP-dependent protein kinase type I-alpha regulatory subunit	0	Cyto	nucleotide binding
TMM43_HUMAN	Transmembrane protein 43	4	ER,Golgi	
B4DPY0_HUMAN	Glutathione peroxidase	0		
HYEP_HUMAN	Epoxide hydrolase 1	0	ER	hydrolase activity
GLU2B_HUMAN	Glucosidase 2 subunit beta	0	ER	calcium ion binding,kinase binding
SNP23_HUMAN	Synaptosomal-associated protein 23	0	PM	transport
CY1_HUMAN	Cytochrome c1, heme protein, mitochondrial	0	Mito	transport,oxidoreductase,metalloprotein,electron carrier activity
COPE_HUMAN	Coatomer subunit epsilon	0	PM,Cyto,Golgi	transport
DPM1_HUMAN	Dolichol-phosphate mannosyltransferase	0	ER,Nuc	
ARL8B_HUMAN	ADP-ribosylation factor-like protein 8B	0		GTPase activity,pyrophosphatase activity
1433T_HUMAN	14-3-3 protein theta	0	Cyto	
ITA2_HUMAN	Integrin alpha-2	1	PM	integrin,calcium ion binding,receptor binding
THY1_HUMAN	Thy-1 membrane glycoprotein	0	PM,ER,Cyto	lipoprotein,receptor binding
NDUB9_HUMAN	NADH dehydrogenase [ubiquinone] 1 beta subcomplex subunit 9	0	Mito	transport,oxidoreductase activity
GNAI1_HUMAN	Guanine nucleotide-binding protein G(i), alpha-1 subunit	0	PM	lipoprotein,GTPase activity,pyrophosphatase activity

GNAI2_HUMAN	Guanine nucleotide-binding protein G(i), alpha-2 subunit	0	PM,Cyto	lipoprotein,GTPase activity,pyrophosphatase activity
AT2B1_HUMAN	Plasma membrane calcium-transporting ATPase 1	7	PM	ATPase activity,transporter activity,pyrophosphatase activity,calcium ion binding
MRP1_HUMAN	Multidrug resistance-associated protein 1	16	PM	ATPase activity,transporter activity,pyrophosphatase activity
LRRC1_HUMAN	Leucine-rich repeat-containing protein 1	0	Cyto	
RALA_HUMAN	Ras-related protein Ral-A	0	PM	lipoprotein,GTPase activity,pyrophosphatase activity
GNA11_HUMAN	Guanine nucleotide-binding protein subunit alpha-11	0	Cyto,PM	GTPase activity,pyrophosphatase activity,ATPase activity
HS71L_HUMAN	Heat shock 70 kDa protein 1L	0		nucleotide binding
PBIP1_HUMAN	Pre-B-cell leukemia transcription factor-interacting protein 1	0	Cyto	cytoskeleton
RGRF1_HUMAN	Ras-specific guanine nucleotide-releasing factor 1	0	PM,Cyto	receptor binding
MYO1B_HUMAN	Myosin-Ib	0		nucleotide binding,pyrophosphatase activity
PHB_HUMAN	Prohibitin	0	PM,Mito	
LRC32_HUMAN	Leucine-rich repeat-containing protein 32	1	PM	
ACTA_HUMAN	Actin, aortic smooth muscle	0	Cyto	cytoskeleton,nucleotide binding
MBLC2_HUMAN	Metallo-beta-lactamase domain-containing protein 2	0		hydrolase activity
TRPM4_HUMAN	Transient receptor potential cation channel subfamily M member 4	5	PM	transport,nucleotide binding,transporter activity,calcium ion binding
LMNA_HUMAN	Lamin-A/C	0	PM,Nuc	lipoprotein
TM9S3_HUMAN	Transmembrane 9 superfamily member 3	9		
ANX11_HUMAN	Annexin A11	0	Cyto	calcium ion binding
SGCD_HUMAN	Delta-sarcoglycan	1	PM,Cyto	cytoskeleton
RPN2_HUMAN	Dolichyl-diphosphooligosaccharide--protein glycosyltransferase subunit 2	4	ER,Nuc	
MGST1_HUMAN	Microsomal glutathione S-transferase 1	1	ER,Mito	
ACTG_HUMAN	Actin, cytoplasmic 2	0	Cyto	cytoskeleton,nucleotide binding,kinase binding
ACTB_HUMAN	Actin, cytoplasmic 1	0	Cyto	cytoskeleton,nucleotide binding,kinase binding
UBIQ_HUMAN	Ubiquitin	0	Cyto,Ribo	ribonucleoprotein
BASI_HUMAN	Basigin	2	PM,Mito,Golgi	transporter activity
RCN1_HUMAN	Reticulocalbin-1	0	ER	calcium ion binding
ATPO_HUMAN	ATP synthase subunit O, mitochondrial	0	PM,Mito	transporter activity,pyrophosphatase activity,ATPase activity
ILK_HUMAN	Integrin-linked protein kinase	0	PM	integrin,nucleotide binding,receptor binding
GNAQ_HUMAN	Guanine nucleotide-binding protein G(q) subunit alpha	0	PM	lipoprotein,GTPase activity,pyrophosphatase activity
Q49AG2_HUMAN	TMED5 protein	1	ER	
LMAN2_HUMAN	Vesicular integral-membrane protein VIP36	1	ER,Golgi	transport,calcium ion binding
MLEC_HUMAN	Malectin	1	ER	
STXB3_HUMAN	Syntaxin-binding protein 3	0	PM,Cyto	transport

AT1A1_HUMAN	Sodium/potassium-transporting ATPase subunit alpha-1	10	PM	ATPase activity,transporter activity,pyrophosphatase activity
EPHA2_HUMAN	Ephrin type-A receptor 2	1	PM	nucleotide binding
PERI_HUMAN	Peripherin	0		
LEMD2_HUMAN	LEM domain-containing protein 2	2	Nuc	
HNRPK_HUMAN	Heterogeneous nuclear ribonucleoprotein K	0	Cyto	ribonucleoprotein
MPIP1_HUMAN	M-phase inducer phosphatase 1	0	Nuc	hydrolase activity
ACTC_HUMAN	Actin, alpha cardiac muscle 1	0	Cyto	cytoskeleton,ATPase activity,pyrophosphatase activity
STX4_HUMAN	Syntaxin-4	1	PM,Cyto,Golgi	transport
RL18_HUMAN	60S ribosomal protein L18	0	Cyto,Ribo	ribonucleoprotein
TOLIP_HUMAN	Toll-interacting protein	0	Cyto,PM	receptor binding
OPRS1_HUMAN	Sigma 1-type opioid receptor	1	PM,ER,Nuc	transport,peptide binding
RS7_HUMAN	40S ribosomal protein S7	0	Cyto,Ribo	ribonucleoprotein
RL7A_HUMAN	60S ribosomal protein L7a	0	Cyto,Ribo	ribonucleoprotein
GBB1_HUMAN	Guanine nucleotide-binding protein G(I)/G(S)/G(T) subunit beta-1	0	PM	pyrophosphatase activity,receptor binding
SC61G_HUMAN	Protein transport protein Sec61 subunit gamma	1	ER,Nuc	transporter activity
BAK_HUMAN	Bcl-2 homologous antagonist/killer	1	ER,Cyto,Mito	
TRAM1_HUMAN	Translocating chain-associated membrane protein 1	8	ER,Nuc	transport
HS90B_HUMAN	Heat shock protein HSP 90-beta	0	Cyto	Chaperone,nucleotide binding
VPP3_HUMAN	V-type proton ATPase 116 kDa subunit a isoform 3	8	PM	transporter activity
PGFRB_HUMAN	Beta-type platelet-derived growth factor receptor	1	PM	nucleotide binding,receptor binding
RL24_HUMAN	60S ribosomal protein L24	0	Cyto,Ribo	ribonucleoprotein
SREC2_HUMAN	Scavenger receptor class F member 2	0		
CE043_HUMAN	UPF0542 protein C5orf43	1		
DAG1_HUMAN	Dystroglycan	1	PM,Cyto	cytoskeleton,calcium ion binding
RAB9A_HUMAN	Ras-related protein Rab-9A	0	PM,ER,Golgi,Nuc	transport,lipoprotein,GTPase activity,pyrophosphatase activity
ATPB_HUMAN	ATP synthase subunit beta, mitochondrial	0	PM,Mito	ATPase activity,transporter activity,pyrophosphatase activity,calcium ion binding,receptor binding
Q5HYD9_HUMAN	Putative uncharacterized protein DKFZp686M0619 (Fragment)	0	Mito	transport
NSMA3_HUMAN	Sphingomyelin phosphodiesterase 4	1	ER,Golgi	hydrolase activity
LNP_HUMAN	Protein lunapark	2		
MFF_HUMAN	Mitochondrial fission factor	1	Mito	
S10AD_HUMAN	Protein S100-A13	0		calcium ion binding
S39AE_HUMAN	Zinc transporter ZIP14	6	PM,Golgi	transporter activity
TOIP1_HUMAN	Torsin-1A-interacting protein 1	0		

NDUA9_HUMAN	NADH dehydrogenase [ubiquinone] 1 alpha subcomplex subunit 9, mitochondrial	0	Mito	transport,oxidoreductase activity
LMAN1_HUMAN	Protein ERGIC-53	1	ER,Golgi,Nuc	transport
CD59_HUMAN	CD59 glycoprotein	0	PM	lipoprotein
MPU1_HUMAN	Mannose-P-dolichol utilization defect 1 protein	6	ER,Nuc	
SGCB_HUMAN	Beta-sarcoglycan	1	PM,Cyto	cytoskeleton
RAB5C_HUMAN	Ras-related protein Rab-5C	0	PM	transport,lipoprotein,GTPase activity,pyrophosphatase activity
MOXD1_HUMAN	DBH-like monooxygenase protein 1	0	ER	oxidoreductase activity
KAP2_HUMAN	cAMP-dependent protein kinase type II-alpha regulatory subunit	0	PM,Cyto	nucleotide binding
CALD1_HUMAN	Caldesmon	0	PM	
PDLI7_HUMAN	PDZ and LIM domain protein 7	0	Cyto	cytoskeleton
RASK_HUMAN	GTPase KRas	0	PM,Golgi	lipoprotein,GTPase activity,pyrophosphatase activity
FKBP8_HUMAN	FK506-binding protein 8	1	PM,ER,Mito,Nuc	calcium ion binding
ENPP1_HUMAN	Ectonucleotide pyrophosphatase/phosphodiesterase family member 1	1	PM	nucleotide binding,pyrophosphatase activity,receptor binding
UBE2N_HUMAN	Ubiquitin-conjugating enzyme E2 N	0	Nuc	nucleotide binding
CAD11_HUMAN	Cadherin-11	1	PM	calcium ion binding
COX5A_HUMAN	Cytochrome c oxidase subunit 5A, mitochondrial	0	Mito	transporter activity,oxidoreductase activity
T179B_HUMAN	Transmembrane protein 179B	3		
ITA3_HUMAN	Integrin alpha-3	2	PM	integrin,calcium ion binding
Q6IPH7_HUMAN	RPL14 protein	0	Cyto,Ribo	ribonucleoprotein
ABD12_HUMAN	Monoacylglycerol lipase ABHD12	1		hydrolase activity
CP51A_HUMAN	Lanosterol 14-alpha demethylase	1	ER,Nuc	metalloprotein,oxidoreductase activity
SCPDH_HUMAN	Probable saccharopine dehydrogenase	1		oxidoreductase activity
ERLN1_HUMAN	Erlin-1	1	ER,Nuc	
PPIC_HUMAN	Peptidyl-prolyl cis-trans isomerase C	1	Cyto	peptide binding
MYO1D_HUMAN	Myosin-Id	0		nucleotide binding,pyrophosphatase activity
FAM3C_HUMAN	Protein FAM3C	1		receptor binding
RAB32_HUMAN	Ras-related protein Rab-32	0	Mito	lipoprotein,nucleotide binding
AT131_HUMAN	Probable cation-transporting ATPase 13A1	7		ATPase activity,transporter activity,pyrophosphatase activity
DCBD2_HUMAN	Discoidin, CUB and LCCL domain-containing protein 2	1	PM	
ARPC2_HUMAN	Actin-related protein 2/3 complex subunit 2	0	PM,Cyto,Golgi	cytoskeleton
ITM2B_HUMAN	Integral membrane protein 2B	1	Golgi	nucleotide binding
TCPG_HUMAN	T-complex protein 1 subunit gamma	0	PM,Cyto	Chaperone,nucleotide binding
RL5_HUMAN	60S ribosomal protein L5	0	Cyto,Ribo	ribonucleoprotein
PPIB_HUMAN	Peptidyl-prolyl cis-trans isomerase B	1	ER	peptide binding

HSP71_HUMAN	Heat shock 70 kDa protein 1	0	ER,Mito	Chaperone,nucleotide binding
FLNA_HUMAN	Filamin-A	0	PM,Cyto,Golgi	cytoskeleton,receptor binding,kinase binding
PLXB2_HUMAN	Plexin-B2	0		
OSTC_HUMAN	Oligosaccharyltransferase complex subunit OSTC	3	ER,Nuc	
1433G_HUMAN	14-3-3 protein gamma	0	Cyto	receptor binding,kinase binding
SNAA_HUMAN	Alpha-soluble NSF attachment protein	0	ER,Golgi	transport
CISD1_HUMAN	CDGSH iron sulfur domain-containing protein 1	1	Mito	
PRDBP_HUMAN	Protein kinase C delta-binding protein	0		kinase binding
H33_HUMAN	Histone H3.3	0	Nuc	
HP1B3_HUMAN	Heterochromatin protein 1-binding protein 3	0	Nuc	
PON2_HUMAN	Serum paraoxonase/arylesterase 2	0	PM	hydrolase activity
SPNS1_HUMAN	Protein spinster homolog 1	11	Mito	transport
OST48_HUMAN	Dolichyl-diphosphooligosaccharide--protein glycosyltransferase 48 kDa subunit	2	ER,Nuc	
PANX1_HUMAN	Pannexin-1	4	PM	transporter activity,receptor binding
COX2_HUMAN	Cytochrome c oxidase subunit 2	2	Mito	transporter activity,oxidoreductase activity
CISD2_HUMAN	CDGSH iron sulfur domain-containing protein 2	1	ER	
ANXA6_HUMAN	Annexin A6	0	Cyto,PM	calcium ion binding
COPG_HUMAN	Coatomer subunit gamma	0	PM,Cyto,Golgi	transport
ERO1A_HUMAN	ERO1-like protein alpha	0	ER,Nuc	transport,nucleotide binding,oxidoreductase activity
ASPH_HUMAN	Aspartyl/asparaginyl beta-hydroxylase	1	ER,Nuc	calcium ion binding,oxidoreductase activity
K2C7_HUMAN	Keratin, type II cytoskeletal 7	0	Cyto	
DDAH2_HUMAN	N(G),N(G)-dimethylarginine dimethylaminohydrolase 2	0	Cyto	hydrolase activity
APMAP_HUMAN	Adipocyte plasma membrane-associated protein	1		hydrolase activity
RS10_HUMAN	40S ribosomal protein S10	0	Cyto,Ribo	ribonucleoprotein
IDHC_HUMAN	Isocitrate dehydrogenase [NADP] cytoplasmic	0	Cyto	nucleotide binding,oxidoreductase activity
LMNB1_HUMAN	Lamin-B1	0	Nuc	lipoprotein
PPGB_HUMAN	Lysosomal protective protein	0	ER,Mito	hydrolase activity
EMD_HUMAN	Emerin	1	ER,Nuc	
SKP1_HUMAN	S-phase kinase-associated protein 1	0	Cyto	
FAS_HUMAN	Fatty acid synthase	0	Cyto,Golgi	transporter activity,oxidoreductase activity
SQSTM_HUMAN	Sequestosome-1	0	Cyto	receptor binding,kinase binding
S12A2_HUMAN	Solute carrier family 12 member 2	12	PM	transporter activity
CALM_HUMAN	Calmodulin	0	PM,Cyto	calcium ion binding
PGRC1_HUMAN	Membrane-associated progesterone receptor component 1	1	ER	
RASN_HUMAN	GTPase NRas	0	PM,Golgi	lipoprotein,GTPase activity,pyrophosphatase activity
BASP1_HUMAN	Brain acid soluble protein 1	0	PM	lipoprotein
ANO6_HUMAN	Anoctamin-6	8		transporter activity,calcium ion binding

AR6P6_HUMAN	ADP-ribosylation factor-like protein 6-interacting protein 6	3		
LPHN2_HUMAN	Latrophilin-2	7	PM	
AT2B4_HUMAN	Plasma membrane calcium-transporting ATPase 4	8	PM	ATPase activity,transporter activity,pyrophosphatase activity,calcium ion binding
NDUAC_HUMAN	NADH dehydrogenase [ubiquinone] 1 alpha subcomplex subunit 12	0	Mito	transport,oxidoreductase activity
GBB4_HUMAN	Guanine nucleotide-binding protein subunit beta-4	0		
CD276_HUMAN	CD276 antigen	1	PM	receptor binding
HMOX1_HUMAN	Heme oxygenase 1	1	PM,ER,Cyto	oxidoreductase activity
NDUS8_HUMAN	NADH dehydrogenase [ubiquinone] iron-sulfur protein 8, mitochondrial	0	Mito	transport,oxidoreductase activity
NDUAD_HUMAN	NADH dehydrogenase [ubiquinone] 1 alpha subcomplex subunit 13	1	Mito	transport,nucleotide binding,oxidoreductase activity
PEX14_HUMAN	Peroxisomal membrane protein PEX14	0	PM	transport
TPSN_HUMAN	Tapasin	1	ER,Golgi,Nuc	transporter activity,pyrophosphatase activity,receptor binding,ATPase activity,peptide binding
GBB2_HUMAN	Guanine nucleotide-binding protein G(I)/G(S)/G(T) subunit beta-2	0		pyrophosphatase activity,GTPase activity
RL28_HUMAN	60S ribosomal protein L28	0	Cyto,Ribo	ribonucleoprotein
RER1_HUMAN	Protein RER1	3	Golgi	
TMED2_HUMAN	Transmembrane emp24 domain-containing protein 2	2	Golgi	transport
HMOX2_HUMAN	Heme oxygenase 2	1	PM,ER	oxidoreductase activity
RS20_HUMAN	40S ribosomal protein S20	0	Cyto,Ribo	ribonucleoprotein
VDAC1_HUMAN	Voltage-dependent anion-selective channel protein 1	0	PM,Mito	transporter activity
1C12_HUMAN	HLA class I histocompatibility antigen, Cw-12 alpha chain	1	PM,Golgi	
VIME_HUMAN	Vimentin	0	PM,Cyto	kinase binding
E41L2_HUMAN	Band 4.1-like protein 2	0	Cyto,PM	cytoskeleton
NNMT_HUMAN	Nicotinamide N-methyltransferase	0	Cyto	
CAB45_HUMAN	45 kDa calcium-binding protein	1	PM,Golgi	calcium ion binding
TRPV2_HUMAN	Transient receptor potential cation channel subfamily V member 2	6	PM,Cyto	transporter activity,calcium ion binding
ACTZ_HUMAN	Alpha-centractin	0	Cyto	cytoskeleton,nucleotide binding
Q7L7Q6_HUMAN	RTN4 isoform B1	2	PM,ER,Nuc	
CC50A_HUMAN	Cell cycle control protein 50A	2		
HNRPF_HUMAN	Heterogeneous nuclear ribonucleoprotein F	0	Nuc	ribonucleoprotein,nucleotide binding
ATPA_HUMAN	ATP synthase subunit alpha, mitochondrial	0	PM,Mito	ATPase activity,transporter activity,pyrophosphatase activity,receptor binding

ATP5H_HUMAN	ATP synthase subunit d, mitochondrial	0	Mito	transporter activity,pyrophosphatase activity,ATPase activity
TM87A_HUMAN	Transmembrane protein 87A	7		
EF1A1_HUMAN	Elongation factor 1-alpha 1	0	Cyto	GTPase activity,pyrophosphatase activity
RS6_HUMAN	40S ribosomal protein S6	0	Cyto,Ribo	ribonucleoprotein
VDAC3_HUMAN	Voltage-dependent anion-selective channel protein 3	0	Mito	transport,nucleotide binding,transporter activity
PKHO2_HUMAN	Pleckstrin homology domain-containing family O member 2	0		
IPO7_HUMAN	Importin-7	0	Cyto	transporter activity
LAP2B_HUMAN	Lamina-associated polypeptide 2, isoforms beta/gamma	1	Nuc	
MPCP_HUMAN	Phosphate carrier protein, mitochondrial	2	PM,Mito	transporter activity
VDAC2_HUMAN	Voltage-dependent anion-selective channel protein 2	0	Mito	transport,nucleotide binding,transporter activity
S10AB_HUMAN	Protein S100-A11	0	Cyto	calcium ion binding
STIM1_HUMAN	Stromal interaction molecule 1	1	PM,ER,Nuc	transport,calcium ion binding
MYH9_HUMAN	Myosin-9	0	PM,Cyto	ATPase activity,pyrophosphatase activity
P4K2A_HUMAN	Phosphatidylinositol 4-kinase type 2-alpha	0	Cyto,PM	nucleotide binding
TM87B_HUMAN	Transmembrane protein 87B	6		
TM173_HUMAN	Transmembrane protein 173	0	PM,ER,Mito	
CD44_HUMAN	CD44 antigen	1	PM,Golgi	
RL6_HUMAN	60S ribosomal protein L6	0	Cyto,Ribo	ribonucleoprotein
GDIB_HUMAN	Rab GDP dissociation inhibitor beta	0	Cyto,Golgi	
VAMP3_HUMAN	Vesicle-associated membrane protein 3	1	PM	
RS9_HUMAN	40S ribosomal protein S9	0	Cyto,Ribo	ribonucleoprotein
ILVBL_HUMAN	Acetolactate synthase-like protein	1		
TMEDA_HUMAN	Transmembrane emp24 domain-containing protein 10	2	Golgi	transport
RAB5B_HUMAN	Ras-related protein Rab-5B	0	PM	transport,lipoprotein,GTPase activity,pyrophosphatase activity
BAG2_HUMAN	BAG family molecular chaperone regulator 2	0		Chaperone
MTCH1_HUMAN	Mitochondrial carrier homolog 1	2	Mito	transport
Q54A51_HUMAN	Cervical EMMPRIN	2	PM,Mito,Golgi	transporter activity
ACAM_HUMAN	Adipocyte adhesion molecule	1	PM	
RL23_HUMAN	60S ribosomal protein L23	0	Cyto,Ribo	ribonucleoprotein
FARP1_HUMAN	FERM, RhoGEF and pleckstrin domain-containing protein 1	0		
VAPA_HUMAN	Vesicle-associated membrane protein-associated protein A	1	PM,ER,Nuc	
AR6P1_HUMAN	ADP-ribosylation factor-like protein 6-interacting protein 1	4	ER,Cyto,Nuc	
ANXA2_HUMAN	Annexin A2	0	PM	calcium ion binding

PLXD1_HUMAN	Plexin-D1	1	PM	
NPTN_HUMAN	Neuroplastin	1	PM	
FLOT2_HUMAN	Flotillin-2	0	PM	
SCRIB_HUMAN	Protein scribble homolog	0	Cyto,PM	
MYPT1_HUMAN	Protein phosphatase 1 regulatory subunit 12A	0	Cyto	hydrolase activity
RL4_HUMAN	60S ribosomal protein L4	0	Cyto,Ribo	ribonucleoprotein
A8K5C2_HUMAN	cDNA FLJ75055	0		
USE1_HUMAN	Vesicle transport protein USE1	1	ER	transport
RL13A_HUMAN	60S ribosomal protein L13a	0	Cyto,Ribo	ribonucleoprotein
RAB5A_HUMAN	Ras-related protein Rab-5A	0	PM	transport,lipoprotein,GTPase activity,pyrophosphatase activity
HS90A_HUMAN	Heat shock protein HSP 90-alpha	0	Cyto	Chaperone,nucleotide binding
CD9_HUMAN	CD9 antigen	4	PM	lipoprotein
SFXN1_HUMAN	Sideroflexin-1	3	Mito	transporter activity
RAB18_HUMAN	Ras-related protein Rab-18	0	PM	transport,lipoprotein,GTPase activity,pyrophosphatase activity
MMP2_HUMAN	72 kDa type IV collagenase	0	PM	calcium ion binding,hydrolase activity
PTRF_HUMAN	Polymerase I and transcript release factor	0	PM,ER,Cyto,Mito	
ACTN1_HUMAN	Alpha-actinin-1	0	Cyto,PM	cytoskeleton,calcium ion binding,receptor binding
ICAM1_HUMAN	Intercellular adhesion molecule 1	1	PM	receptor binding
RLA2_HUMAN	60S acidic ribosomal protein P2	0	Cyto,Ribo	ribonucleoprotein
VKOR1_HUMAN	Vitamin K epoxide reductase complex subunit 1	3	ER	oxidoreductase activity
CS010_HUMAN	UPF0556 protein C19orf10	0		receptor binding
K1C19_HUMAN	Keratin, type I cytoskeletal 19	0	PM	
CYFP1_HUMAN	Cytoplasmic FMR1-interacting protein 1	0	Cyto,PM	
SE1L1_HUMAN	Protein sel-1 homolog 1	1	ER	
SFXN3_HUMAN	Sideroflexin-3	1	Mito	transporter activity
TMED5_HUMAN	Transmembrane emp24 domain-containing protein 5	2	ER	
FNDC1_HUMAN	Fibronectin type III domain-containing protein 1	0		
AHNK_HUMAN	Neuroblast differentiation-associated protein AHNAK	0	Nuc	
Q29669_HUMAN	MHC class I antigen HLA-B35	1	PM,Golgi	
RL17_HUMAN	60S ribosomal protein L17	0	Cyto,Ribo	ribonucleoprotein
SYPL1_HUMAN	Synaptophysin-like protein 1	3	PM	transporter activity
MTX1_HUMAN	Metaxin-1	1	Mito	transport
UGGG1_HUMAN	UDP-glucose:glycoprotein glucosyltransferase 1	1	ER	
RHOA_HUMAN	Transforming protein RhoA	0	Cyto,PM	lipoprotein,cytoskeleton,GTPase activity,pyrophosphatase activity
SFPQ_HUMAN	Splicing factor, proline- and glutamine-rich	0	Nuc	nucleotide binding

AT1B3_HUMAN	Sodium/potassium-transporting ATPase subunit beta-3	1	PM	transporter activity,pyrophosphatase activity,ATPase activity
HSPB1_HUMAN	Heat shock protein beta-1	0	Cyto,PM	
ANPRB_HUMAN	Atrial natriuretic peptide receptor B	0	PM	nucleotide binding,peptide binding
BAX_HUMAN	Apoptosis regulator BAX	1	ER,Cyto,Mito,Nuc	
NDUA3_HUMAN	NADH dehydrogenase [ubiquinone] 1 alpha subcomplex subunit 3	1	Mito	transport,oxidoreductase activity
NOTC2_HUMAN	Neurogenic locus notch homolog protein 2	2	PM	calcium ion binding
VKORL_HUMAN	Vitamin K epoxide reductase complex subunit 1-like protein 1	2		
SEPT9_HUMAN	Septin-9	0	PM	GTPase activity,pyrophosphatase activity
PSMD6_HUMAN	26S proteasome non-ATPase regulatory subunit 6	0	Cyto	pyrophosphatase activity,ATPase activity
RAB34_HUMAN	Ras-related protein Rab-34	0	Cyto,Golgi	transport,lipoprotein,GTPase activity,pyrophosphatase activity
B4DMH5_HUMAN	cDNA FLJ55107, highly similar to Cell division control protein 42 homolog	0		
SCRB2_HUMAN	Lysosome membrane protein 2	2	PM	
ITA4_HUMAN	Integrin alpha-4	1	PM	cytoskeleton,integrin,calcium ion binding
HAS1_HUMAN	Hyaluronan synthase 1	7	PM	
FSCN1_HUMAN	Fascin	0	PM	
Q4W6C4_HUMAN	MHC class I antigen (Fragment)	0	PM,Golgi	
MARCS_HUMAN	Myristoylated alanine-rich C-kinase substrate	0	Cyto	lipoprotein,cytoskeleton,kinase binding
CS052_HUMAN	Uncharacterized protein C19orf52	0		
S10AG_HUMAN	Protein S100-A16	0	Nuc	calcium ion binding
YIF1A_HUMAN	Protein YIF1A	5	ER,Golgi	transport
SPCS3_HUMAN	Signal peptidase complex subunit 3	1	ER,Nuc	hydrolase activity
FERM2_HUMAN	Fermitin family homolog 2	0	Cyto,PM	cytoskeleton
TPP1_HUMAN	Tripeptidyl-peptidase 1	0	Mito	hydrolase activity,peptide binding
NNTM_HUMAN	NAD(P) transhydrogenase, mitochondrial	12	Mito	nucleotide binding,transporter activity,oxidoreductase activity
PSD11_HUMAN	26S proteasome non-ATPase regulatory subunit 11	0	Cyto	
RAB13_HUMAN	Ras-related protein Rab-13	0	PM,Golgi	transport,lipoprotein,GTPase activity,pyrophosphatase activity
Q8MGZ8_HUMAN	MHC class I antigen (Fragment)	0	PM,Golgi	
QCR1_HUMAN	Cytochrome b-c1 complex subunit 1, mitochondrial	0	Mito	transporter activity,oxidoreductase activity
RL22_HUMAN	60S ribosomal protein L22	0	Cyto,Ribo	ribonucleoprotein
QCR8_HUMAN	Cytochrome b-c1 complex subunit 8	0	Mito	transporter activity,oxidoreductase activity
ZNT9_HUMAN	Zinc transporter 9	5		transport,nucleotide binding,transporter activity
ANXA4_HUMAN	Annexin A4	0		calcium ion binding

AT5F1_HUMAN	ATP synthase subunit b, mitochondrial	0	Mito	transporter activity,pyrophosphatase activity,ATPase activity
ITA5_HUMAN	Integrin alpha-5	1	PM	integrin,calcium ion binding,receptor binding
ATP5L_HUMAN	ATP synthase subunit g, mitochondrial	0	Mito	transporter activity,pyrophosphatase activity,ATPase activity
B3KUB6_HUMAN	cDNA FLJ39529 fis, clone PUAEN2004067, highly similar to Band 4.1-like protein 1	0		
XRP2_HUMAN	Protein XRP2	0	PM	lipoprotein,nucleotide binding
B2R7T6_HUMAN	cDNA, FLJ93596, highly similar to Homo sapiens sulfide quinone reductase-like (yeast) (SQRDL), mRNA	0		
H15_HUMAN	Histone H1.5	0	Nuc	
SEP11_HUMAN	Septin-11	0		nucleotide binding
RS24_HUMAN	40S ribosomal protein S24	0	Cyto,Ribo	ribonucleoprotein,nucleotide binding
VASN_HUMAN	Vasorin	1		
RS4X_HUMAN	40S ribosomal protein S4, X isoform	0	Cyto,Ribo	ribonucleoprotein
SC23A_HUMAN	Protein transport protein Sec23A	0	ER,Cyto,Golgi	transport
NDUS1_HUMAN	NADH-ubiquinone oxidoreductase 75 kDa subunit, mitochondrial	0	Mito	transport,metalloprotein,oxidoreductase activity
PLXA1_HUMAN	Plexin-A1	2	PM	
LMNB2_HUMAN	Lamin-B2	0	Nuc	lipoprotein
PTPRD_HUMAN	Receptor-type tyrosine-protein phosphatase delta	1	PM	hydrolase activity
PGS1_HUMAN	Biglycan	0	PM	
TAP2_HUMAN	Antigen peptide transporter 2	6	PM,ER,Cyto	ATPase activity,transporter activity,pyrophosphatase activity,receptor binding,peptide binding
MRC2_HUMAN	C-type mannose receptor 2	1		calcium ion binding
MYH10_HUMAN	Myosin-10	0	PM	ATPase activity,pyrophosphatase activity
PPBT_HUMAN	Alkaline phosphatase, tissue-nonspecific isozyme	0	PM	lipoprotein,metalloprotein,hydrolase activity
ARPC3_HUMAN	Actin-related protein 2/3 complex subunit 3	0	Cyto	cytoskeleton
LANC1_HUMAN	LanC-like protein 1	0	Cyto,PM	receptor binding
PCDG4_HUMAN	Protocadherin gamma-A4	1	PM	calcium ion binding
F162A_HUMAN	UPF0389 protein FAM162A	1		
RADI_HUMAN	Radixin	0	Cyto,PM	cytoskeleton
GCN1L_HUMAN	Translational activator GCN1	0	Ribo	
RL10_HUMAN	60S ribosomal protein L10	0	ER,Cyto,Ribo, Mito	ribonucleoprotein
CCD56_HUMAN	Coiled-coil domain-containing protein 56	1	Mito	
NDUB5_HUMAN	NADH dehydrogenase [ubiquinone] 1 beta subcomplex subunit 5, mitochondrial	1	Mito	transport,oxidoreductase activity
B5A9M9_HUMAN	MHC class I antigen (Fragment)	0		

RECK_HUMAN	Reversion-inducing cysteine-rich protein with Kazal motifs	1	PM	lipoprotein
RS16_HUMAN	40S ribosomal protein S16	0	Cyto,Ribo	ribonucleoprotein
ACTN4_HUMAN	Alpha-actinin-4	0	Cyto	calcium ion binding,receptor binding
DYHC1_HUMAN	Cytoplasmic dynein 1 heavy chain 1	0	Cyto,Golgi	ATPase activity,pyrophosphatase activity
CPNE2_HUMAN	Copine-2	0		
MCFD2_HUMAN	Multiple coagulation factor deficiency protein 2	0	ER,Golgi	transport,calcium ion binding
ERLN2_HUMAN	Erlin-2	0	ER,Nuc	
ANO10_HUMAN	Anoctamin-10	8		transporter activity,calcium ion binding
COX6C_HUMAN	Cytochrome c oxidase subunit 6C	1	Mito	transporter activity,oxidoreductase activity
H12_HUMAN	Histone H1.2	0	Nuc	
EZRI_HUMAN	Ezrin	0	PM,Cyto	cytoskeleton
HSP7C_HUMAN	Heat shock cognate 71 kDa protein	0	Cyto	Chaperone,ATPase activity,pyrophosphatase activity
CYB5_HUMAN	Cytochrome b5	1	PM,ER,Mito,Cyto	transporter activity,oxidoreductase activity
TCPE_HUMAN	T-complex protein 1 subunit epsilon	0	Cyto	Chaperone,nucleotide binding
Q32Q12_HUMAN	Nucleoside diphosphate kinase	0	Cyto	nucleotide binding,hydrolase activity
RL7_HUMAN	60S ribosomal protein L7	0	Cyto,Ribo	ribonucleoprotein
1433E_HUMAN	14-3-3 protein epsilon	0	Cyto,Mito	
B4DDF9_HUMAN	cDNA FLJ52218, highly similar to Annexin A4	0		
1B58_HUMAN	HLA class I histocompatibility antigen, B-58 alpha chain	1	PM,Golgi	
PLPL6_HUMAN	Neuropathy target esterase	1	PM,ER,Nuc	hydrolase activity
BAP31_HUMAN	B-cell receptor-associated protein 31	3	PM,ER,Cyto,Golgi,Nuc	transport,receptor binding
KPCA_HUMAN	Protein kinase C alpha type	0	PM,Cyto,Mito	nucleotide binding,calcium ion binding
AL3A2_HUMAN	Fatty aldehyde dehydrogenase	1	PM,ER,Mito	oxidoreductase activity
RS11_HUMAN	40S ribosomal protein S11	0	Cyto,Ribo	ribonucleoprotein
B7Z9B1_HUMAN	cDNA FLJ52398, highly similar to Cadherin-13	0		
ARF5_HUMAN	ADP-ribosylation factor 5	0	PM,Golgi	transport,lipoprotein,GTPase activity,pyrophosphatase activity
AMPN_HUMAN	Aminopeptidase N	1	PM,Cyto	hydrolase activity,peptide binding
1433Z_HUMAN	14-3-3 protein zeta/delta	0	Cyto,Mito	oxidoreductase activity
PGFRA_HUMAN	Alpha-type platelet-derived growth factor receptor	3	PM	nucleotide binding,receptor binding
PLEC1_HUMAN	Plectin-1	0	PM,Cyto	cytoskeleton
QCR2_HUMAN	Cytochrome b-c1 complex subunit 2, mitochondrial	0	Mito	transport,oxidoreductase,hydrolase activity
RAP2B_HUMAN	Ras-related protein Rap-2b	0	PM	lipoprotein,GTPase activity,pyrophosphatase activity
EGFR_HUMAN	Epidermal growth factor receptor	2	PM	nucleotide binding
LDHA_HUMAN	L-lactate dehydrogenase A chain	0	Cyto	oxidoreductase activity
CO6A1_HUMAN	Collagen alpha-1(VI) chain	0	PM	
RALB_HUMAN	Ras-related protein Ral-B	0	PM	lipoprotein,GTPase activity,pyrophosphatase activity

NP1L1_HUMAN	Nucleosome assembly protein 1-like 1	0	Nuc	lipoprotein
MMP14_HUMAN	Matrix metalloproteinase-14	1	PM	calcium ion binding,hydrolase activity
MFN2_HUMAN	Mitofusin-2	1	Cyto,Mito	GTPase activity,pyrophosphatase activity
T161A_HUMAN	Transmembrane protein 161A	8		
POK2_HUMAN	HERV-K_7p22.1 provirus ancestral Pol protein	0	Cyto,PM	transport,lipoprotein,hydrolase activity
RS8_HUMAN	40S ribosomal protein S8	0	Cyto,Ribo	ribonucleoprotein
TMED1_HUMAN	Transmembrane emp24 domain-containing protein 1	1	PM	receptor binding
C109A_HUMAN	Coiled-coil domain-containing protein 109A	3		
CAZA1_HUMAN	F-actin-capping protein subunit alpha-1	0		
ACLY_HUMAN	ATP-citrate synthase	0	Cyto	nucleotide binding
H4_HUMAN	Histone H4	0	Nuc	
WDR1_HUMAN	WD repeat-containing protein 1	0	Cyto	cytoskeleton
ML12A_HUMAN	Myosin regulatory light chain 12A	0		pyrophosphatase activity,calcium ion binding
SYRC_HUMAN	Arginyl-tRNA synthetase, cytoplasmic	0	Cyto	nucleotide binding
TLN1_HUMAN	Talin-1	0	PM,Cyto	cytoskeleton
A8ILL3_HUMAN	MHC class I antigen (Fragment)	0		
RL21_HUMAN	60S ribosomal protein L21	0	Cyto,Ribo	ribonucleoprotein
COR1B_HUMAN	Coronin-1B	0	Cyto	cytoskeleton
FLNC_HUMAN	Filamin-C	0	Cyto,PM	cytoskeleton
CAZA2_HUMAN	F-actin-capping protein subunit alpha-2	0	PM,Cyto,Mito	kinase binding
CNTP1_HUMAN	Contactin-associated protein 1	1	PM	receptor binding
OCC1_HUMAN	Putative overexpressed in colon carcinoma 1 protein	0		
B2L13_HUMAN	Bcl-2-like protein 13	1	Mito	
B2RDY9_HUMAN	Adenylyl cyclase-associated protein	0		
RAP2C_HUMAN	Ras-related protein Rap-2c	0	PM	lipoprotein,GTPase activity,pyrophosphatase activity
DDR2_HUMAN	Discoidin domain-containing receptor 2	1	PM	nucleotide binding
STOM_HUMAN	Erythrocyte band 7 integral membrane protein	1	PM,ER,Mito,Golgi,Cyto	lipoprotein,nucleotide binding,receptor binding,kinase binding
Q1EJP6_HUMAN	MHC class I antigen (Fragment)	0	PM,Golgi	
PALLD_HUMAN	Palladin	0	Cyto	cytoskeleton
MYADM_HUMAN	Myeloid-associated differentiation marker	8		
PDC6I_HUMAN	Programmed cell death 6-interacting protein	0	Cyto	transport
Q52YL7_HUMAN	MHC class I antigen	1	PM,Golgi	
B1B6Q4_HUMAN	MHC class I antigen (Fragment)	0		
ANXA1_HUMAN	Annexin A1	0	Cyto,PM	calcium ion binding,receptor binding
CAV2_HUMAN	Caveolin-2	1	PM,Cyto,Mito,Golgi	receptor binding,kinase binding
PRDX1_HUMAN	Peroxiredoxin-1	0	Cyto,Mito	oxidoreductase activity

2AAA_HUMAN	Serine/threonine-protein phosphatase 2A 65 kDa regulatory subunit A alpha isoform	0	Cyto,Mito	
TM109_HUMAN	Transmembrane protein 109	5	ER,Nuc	
TERA_HUMAN	Transitional endoplasmic reticulum ATPase	0	ER,Cyto	transport,ATPase activity,pyrophosphatase activity
ERGI1_HUMAN	Endoplasmic reticulum-Golgi intermediate compartment protein 1	2	ER,Golgi	transport
PPIA_HUMAN	Peptidyl-prolyl cis-trans isomerase A	0	Cyto	peptide binding
SEPT7_HUMAN	Septin-7	0	Cyto	nucleotide binding
GRP75_HUMAN	Stress-70 protein, mitochondrial	0	Mito	Chaperone,nucleotide binding
RASEF_HUMAN	RAS and EF-hand domain-containing protein	0	Cyto	nucleotide binding,calcium ion binding
TM119_HUMAN	Transmembrane protein 119	2		
UBA1_HUMAN	Ubiquitin-like modifier-activating enzyme 1	0		nucleotide binding
Q86X69_HUMAN	PON2 protein	0	PM	hydrolase activity
DAB2_HUMAN	Disabled homolog 2	0		
EHD1_HUMAN	EH domain-containing protein 1	0	PM	GTPase activity,pyrophosphatase activity,calcium ion binding
PCYOX_HUMAN	Prenylcysteine oxidase 1	0	PM	transporter activity,pyrophosphatase activity,oxidoreductase activity,ATPase activity
S10A6_HUMAN	Protein S100-A6	0	Nuc	transporter activity,calcium ion binding
TMOD3_HUMAN	Tropomodulin-3	0	Cyto	cytoskeleton
SGCE_HUMAN	Epsilon-sarcoglycan	1	PM,Cyto	cytoskeleton,calcium ion binding
NRP1_HUMAN	Neuropilin-1	1	PM	
A8K968_HUMAN	cDNA FLJ77757	0		
VAT1_HUMAN	Synaptic vesicle membrane protein VAT-1 homolog	0	Nuc	oxidoreductase activity
ATPG_HUMAN	ATP synthase subunit gamma, mitochondrial	0	Mito	transporter activity,pyrophosphatase activity,ATPase activity
MAP4_HUMAN	Microtubule-associated protein 4	0		
SNTB2_HUMAN	Beta-2-syntrophin	0	Cyto,PM	cytoskeleton,calcium ion binding
VINC_HUMAN	Vinculin	0	PM,Cyto	lipoprotein,cytoskeleton,oxidoreductase activity
EF2_HUMAN	Elongation factor 2	0	Cyto	GTPase activity,pyrophosphatase activity
EHD2_HUMAN	EH domain-containing protein 2	0	PM	GTPase activity,pyrophosphatase activity,calcium ion binding
RL38_HUMAN	60S ribosomal protein L38	0	Cyto,Ribo	ribonucleoprotein
S10AA_HUMAN	Protein S100-A10	1		calcium ion binding,receptor binding
RAP2A_HUMAN	Ras-related protein Rap-2a	0	PM	lipoprotein,GTPase activity,pyrophosphatase activity
TBB6_HUMAN	Tubulin beta-6 chain	0		GTPase activity,pyrophosphatase activity
CHMP6_HUMAN	Charged multivesicular body protein 6	0		transport,lipoprotein
COPB_HUMAN	Coatomer subunit beta	0	PM,Cyto,Golgi	transport
TBB4_HUMAN	Tubulin beta-4 chain	0	PM,Cyto	GTPase activity,pyrophosphatase activity

RINI_HUMAN	Ribonuclease inhibitor	0	Cyto	
TBB2C_HUMAN	Tubulin beta-2C chain	0	Cyto	GTPase activity,pyrophosphatase activity,receptor binding
TBB2A_HUMAN	Tubulin beta-2A chain	0	PM,Cyto	GTPase activity,pyrophosphatase activity,receptor binding
KINH_HUMAN	Kinesin-1 heavy chain	0	Cyto	cytoskeleton,nucleotide binding,pyrophosphatase activity
CMC1_HUMAN	Calcium-binding mitochondrial carrier protein Aralar1	0	Mito	transporter activity,calcium ion binding
C9JGY8_HUMAN	Putative uncharacterized protein JAZF1	0		
GSTP1_HUMAN	Glutathione S-transferase P	0		
FLNB_HUMAN	Filamin-B	0	Cyto	cytoskeleton
TBB5_HUMAN	Tubulin beta chain	0	PM,Cyto	GTPase activity,pyrophosphatase activity,receptor binding
TITIN_HUMAN	Titin	0	Cyto	nucleotide binding,calcium ion binding
ARP3_HUMAN	Actin-related protein 3	0	PM,Cyto,Golgi	cytoskeleton,nucleotide binding
SYNC_HUMAN	Asparaginyl-tRNA synthetase, cytoplasmic	0	Cyto	nucleotide binding
CN37_HUMAN	2~,3~-cyclic-nucleotide 3~-phosphodiesterase	0	Ext.	hydrolase activity
ARF1_HUMAN	ADP-ribosylation factor 1	0	PM,Cyto,Golgi	transport,lipoprotein,GTPase activity,pyrophosphatase activity
5NTD_HUMAN	5~-nucleotidase	2	PM	lipoprotein,metalloprotein,nucleotide binding,hydrolase activity
MVP_HUMAN	Major vault protein	0	Cyto	transport,ribonucleoprotein
KPYM_HUMAN	Pyruvate kinase isozymes M1/M2	0	Cyto,Mito	metalloprotein,nucleotide binding
SERA_HUMAN	D-3-phosphoglycerate dehydrogenase	0		nucleotide binding,oxidoreductase activity
PTGIS_HUMAN	Prostacyclin synthase	1	ER	oxidoreductase activity
TBB3_HUMAN	Tubulin beta-3 chain	0	PM	lipoprotein,GTPase activity,pyrophosphatase activity,peptide binding
MX2_HUMAN	Interferon-induced GTP-binding protein Mx2	0	Cyto	GTPase activity,pyrophosphatase activity
COR1C_HUMAN	Coronin-1C	0		
SEPT2_HUMAN	Septin-2	0	Cyto,PM	nucleotide binding
VA0D1_HUMAN	V-type proton ATPase subunit d 1	0	PM	transporter activity,pyrophosphatase activity,ATPase activity
IF5A1_HUMAN	Eukaryotic translation initiation factor 5A-1	0	PM,ER,Cyto,Nuc	
PARK7_HUMAN	Protein DJ-1	0	Cyto,Mito	Chaperone,oxidoreductase activity
TM138_HUMAN	Transmembrane protein 138	4		
GGT5_HUMAN	Gamma-glutamyltransferase 5	1	PM	
RFTN1_HUMAN	Raftlin	0	PM	lipoprotein,nucleotide binding
Q53XZ0_HUMAN	Interferon induced transmembrane protein 1 (9-27)	2	PM	
K2C8_HUMAN	Keratin, type II cytoskeletal 8	0	Cyto,PM	

GBG12_HUMAN	Guanine nucleotide-binding protein G(I)/G(S)/G(O) subunit gamma-12	0	PM	lipoprotein
MOES_HUMAN	Moesin	0	Cyto,PM	cytoskeleton,receptor binding
AMRP_HUMAN	Alpha-2-macroglobulin receptor-associated protein	1	PM,ER,Cyto	lipoprotein,calcium ion binding,receptor binding
TCPZ_HUMAN	T-complex protein 1 subunit zeta	0	Cyto	Chaperone,nucleotide binding
VASP_HUMAN	Vasodilator-stimulated phosphoprotein	0	PM,Cyto	cytoskeleton
TBA1B_HUMAN	Tubulin alpha-1B chain	0		GTPase activity,pyrophosphatase activity
LEG1_HUMAN	Galectin-1	0	Ext.	
GPDM_HUMAN	Glycerol-3-phosphate dehydrogenase, mitochondrial	1	Mito	calcium ion binding,oxidoreductase activity
TPT1L_HUMAN	TPT1-like protein	0		
TBA1C_HUMAN	Tubulin alpha-1C chain	0		GTPase activity,pyrophosphatase activity
RANG_HUMAN	Ran-specific GTPase-activating protein	0	Nuc	
TBA1A_HUMAN	Tubulin alpha-1A chain	0	Cyto	GTPase activity,pyrophosphatase activity
CD81_HUMAN	CD81 antigen	4	PM	
CAV1_HUMAN	Caveolin-1	1	PM,ER,Cyto,Mito ,Golgi	lipoprotein,kinase binding
ARF3_HUMAN	ADP-ribosylation factor 3	0	Golgi	transport,lipoprotein,GTPase activity,pyrophosphatase activity
PRAF3_HUMAN	PRA1 family protein 3	3	ER, Cyto	
ANXA5_HUMAN	Annexin A5	0		calcium ion binding,receptor binding
IQGA1_HUMAN	Ras GTPase-activating-like protein IQGAP1	0	PM	
SC31A_HUMAN	Protein transport protein Sec31A	0	PM,ER,Golgi,Cyt o	transport
ENOA_HUMAN	Alpha-enolase	0	PM,Cyto	hydrolase activity
PLIN3_HUMAN	Perilipin-3	0	PM,Cyto,Golgi	transport
FRIL_HUMAN	Ferritin light chain	0	Cyto,Ribo	metalloprotein,oxidoreductase activity
AK1A1_HUMAN	Alcohol dehydrogenase [NADP+]	0	PM,Cyto	oxidoreductase activity
TAGL2_HUMAN	Transgelin-2	0	PM,Nuc	
KCD12_HUMAN	BTB/POZ domain-containing protein KCTD12	0	PM	transporter activity
MAVS_HUMAN	Mitochondrial antiviral-signaling protein	1	Mito	
SYG_HUMAN	Glycyl-tRNA synthetase	0	Cyto,Mito	nucleotide binding
RS5_HUMAN	40S ribosomal protein S5	0	Cyto,Ribo	ribonucleoprotein
MYL6_HUMAN	Myosin light polypeptide 6	0		pyrophosphatase activity,calcium ion binding,ATPase activity
K22E_HUMAN	Keratin, type II cytoskeletal 2 epidermal	0		
EPCR_HUMAN	Endothelial protein C receptor	1	PM	
CAN1_HUMAN	Calpain-1 catalytic subunit	0	Cyto,PM	calcium ion binding,hydrolase activity
ALDOA_HUMAN	Fructose-bisphosphate aldolase A	0	Ext.	
SRC8_HUMAN	Src substrate cortactin	0	Cyto	cytoskeleton

K1C10_HUMAN	Keratin, type I cytoskeletal 10	0		
CD109_HUMAN	CD109 antigen	0	PM	lipoprotein
ENDD1_HUMAN	Endonuclease domain-containing 1 protein	3		hydrolase activity
AT11C_HUMAN	Probable phospholipid-transporting ATPase IG	7		ATPase activity,transporter activity,pyrophosphatase activity
HSP74_HUMAN	Heat shock 70 kDa protein 4	0	Cyto	nucleotide binding
AHNK2_HUMAN	Protein AHNAK2	0	Nuc	
ISLR_HUMAN	Immunoglobulin superfamily containing leucine-rich repeat protein	0		
VTA1_HUMAN	Vacuolar protein sorting-associated protein VTA1 homolog	0	Cyto	transport
M2OM_HUMAN	Mitochondrial 2-oxoglutarate/malate carrier protein	0	PM,Mito	transporter activity
MIF_HUMAN	Macrophage migration inhibitory factor	0	Ext.	receptor binding
COF1_HUMAN	Cofilin-1	0	Cyto	cytoskeleton
G3P_HUMAN	Glyceraldehyde-3-phosphate dehydrogenase	0	Cyto	nucleotide binding,oxidoreductase activity
IPO9_HUMAN	Importin-9	0	Cyto	transporter activity
TCPB_HUMAN	T-complex protein 1 subunit beta	0	Cyto	Chaperone,nucleotide binding
A1L0S7_HUMAN	TNS1 protein (Fragment)	0	Cyto,PM	cytoskeleton
K2C1_HUMAN	Keratin, type II cytoskeletal 1	0	PM	
LPP3_HUMAN	Lipid phosphate phosphohydrolase 3	6	PM,ER,Cyto,Golgi,Nuc	hydrolase activity
MRP4_HUMAN	Multidrug resistance-associated protein 4	11	PM	ATPase activity,transporter activity,pyrophosphatase activity,oxidoreductase activity
NEP_HUMAN	Neprilysin	1	PM	hydrolase activity,peptide binding
ARC1B_HUMAN	Actin-related protein 2/3 complex subunit 1B	0	Cyto	cytoskeleton
A6NGN7_HUMAN	Ribosomal protein L1	0		
KHDR1_HUMAN	KH domain-containing, RNA-binding, signal transduction-associated protein 1	0	Nuc	
NB5R1_HUMAN	NADH-cytochrome b5 reductase 1	1		oxidoreductase activity
CLIC1_HUMAN	Chloride intracellular channel protein 1	0	PM,Cyto	transporter activity
COPB2_HUMAN	Coatomer subunit beta~	0	PM,Cyto,Golgi	transport
TCPQ_HUMAN	T-complex protein 1 subunit theta	0	Cyto	Chaperone,ATPase activity,pyrophosphatase activity
TIAM1_HUMAN	T-lymphoma invasion and metastasis-inducing protein 1	0	PM,Cyto	lipoprotein,receptor binding
FPRP_HUMAN	Prostaglandin F2 receptor negative regulator	1	ER,Golgi	
COPA_HUMAN	Coatomer subunit alpha	0	PM,ER,Cyto,Golgi	transport,calcium ion binding,receptor binding
MK01_HUMAN	Mitogen-activated protein kinase 1	0	Cyto	nucleotide binding,kinase binding
PSD12_HUMAN	26S proteasome non-ATPase regulatory subunit 12	0	Cyto	
S39A7_HUMAN	Zinc transporter SLC39A7	7		transporter activity

CPNS1_HUMAN	Calpain small subunit 1	0	Cyto,PM	calcium ion binding,hydrolase activity
G6PI_HUMAN	Glucose-6-phosphate isomerase	0	Cyto	receptor binding
K2C5_HUMAN	Keratin, type II cytoskeletal 5	0		cytoskeleton
AGK_HUMAN	Acylglycerol kinase, mitochondrial	0	Mito	nucleotide binding
KDM5B_HUMAN	Lysine-specific demethylase 5B	0	Nuc	oxidoreductase activity
K1C14_HUMAN	Keratin, type I cytoskeletal 14	0	Cyto	
KANK2_HUMAN	KN motif and ankyrin repeat domain-containing protein 2	0		
NXP20_HUMAN	Protein NOXP20	0	Cyto	
TAP1_HUMAN	Antigen peptide transporter 1	7	PM,ER	ATPase activity,transporter activity,pyrophosphatase activity
TMTC3_HUMAN	Transmembrane and TPR repeat-containing protein 3	9		
DCTN2_HUMAN	Dynactin subunit 2	0	Cyto	cytoskeleton,pyrophosphatase activity
GFPT2_HUMAN	Glucosamine--fructose-6-phosphate aminotransferase [isomerizing] 2	0		
LDHB_HUMAN	L-lactate dehydrogenase B chain	0	Cyto	nucleotide binding,oxidoreductase activity
IPO5_HUMAN	Importin-5	0	Cyto	transporter activity
CAN5_HUMAN	Calpain-5	0		hydrolase activity
MK04_HUMAN	Mitogen-activated protein kinase 4	0		nucleotide binding
Q6S4P3_HUMAN	Ferritin	0	Cyto,Ribo	metalloprotein,oxidoreductase activity
K1C9_HUMAN	Keratin, type I cytoskeletal 9	0		
MDHM_HUMAN	Malate dehydrogenase, mitochondrial	0	Mito	oxidoreductase activity
GELS_HUMAN	Gelsolin	0	Cyto	cytoskeleton,calcium ion binding
Q1WWL2_HUMAN	PTGFRN protein (Fragment)	1	ER,Golgi	
RAI14_HUMAN	Ankycorbin	0	Cyto,Mito	cytoskeleton
CAN2_HUMAN	Calpain-2 catalytic subunit	0	Cyto,PM	calcium ion binding,hydrolase activity
K2C6C_HUMAN	Keratin, type II cytoskeletal 6C	0		
S27A3_HUMAN	Long-chain fatty acid transport protein 3	1	Mito	nucleotide binding
SPTB2_HUMAN	Spectrin beta chain, brain 1	0	PM,Cyto	cytoskeleton
TKT_HUMAN	Transketolase	0	Cyto	calcium ion binding
ADDG_HUMAN	Gamma-adducin	0	Cyto,PM	cytoskeleton,kinase binding
SPTA2_HUMAN	Spectrin alpha chain, brain	0	PM,Cyto	cytoskeleton,calcium ion binding
GDIR1_HUMAN	Rho GDP-dissociation inhibitor 1	0	PM,Cyto	
JAK1_HUMAN	Tyrosine-protein kinase JAK1	0	Nuc	nucleotide binding,receptor binding
PLST_HUMAN	Plastin-3	0	Cyto	calcium ion binding
KCC2D_HUMAN	Calcium/calmodulin-dependent protein kinase type II delta chain	0	PM,Cyto	nucleotide binding
DPYL1_HUMAN	Dihydropyrimidinase-related protein 1	0	Cyto	hydrolase activity
MAP1B_HUMAN	Microtubule-associated protein 1B	0	PM,Cyto	

ZCH18_HUMAN	Zinc finger CCCH domain-containing protein 18	0	Nuc	
LASP1_HUMAN	LIM and SH3 domain protein 1	0	Cyto,PM	transport,cytoskeleton
XPO2_HUMAN	Exportin-2	0	Cyto	transporter activity
K2C6B_HUMAN	Keratin, type II cytoskeletal 6B	0		
ALBU_HUMAN	Serum albumin	0	Ext.	
ACE_HUMAN	Angiotensin-converting enzyme	1	PM	receptor binding,hydrolase activity,peptide binding
DCTN1_HUMAN	Dynactin subunit 1	0	Cyto	cytoskeleton,pyrophosphatase activity
K6PP_HUMAN	6-phosphofructokinase type C	0	Cyto	nucleotide binding
G6PD_HUMAN	Glucose-6-phosphate 1-dehydrogenase	0	PM,Cyto	nucleotide binding,oxidoreductase activity
PGK1_HUMAN	Phosphoglycerate kinase 1	0	Cyto	nucleotide binding
KCC2B_HUMAN	Calcium/calmodulin-dependent protein kinase type II beta chain	0		nucleotide binding
KCC2A_HUMAN	Calcium/calmodulin-dependent protein kinase type II alpha chain	0	PM	nucleotide binding,receptor binding
CRYAB_HUMAN	Alpha-crystallin B chain	0	PM,Golgi	
SYNE3_HUMAN	Nesprin-3	1		
STAT1_HUMAN	Signal transducer and activator of transcription 1-alpha/beta	0	Cyto	calcium ion binding
TM14C_HUMAN	Transmembrane protein 14C	4	Mito	
CP1B1_HUMAN	Cytochrome P450 1B1	0	ER,Nuc	metalloprotein,oxidoreductase activity
LEG3_HUMAN	Galectin-3	0	PM	
DPP4_HUMAN	Dipeptidyl peptidase 4	1	PM,ER,Golgi	hydrolase activity,peptide binding
A1L3U3_HUMAN	ABCA8 protein	13		
TPIS_HUMAN	Triosephosphate isomerase	0	Cyto	
AKA12_HUMAN	A-kinase anchor protein 12	0	Cyto,PM	cytoskeleton
UN84A_HUMAN	Protein unc-84 homolog A	2	Nuc,Cyto	
TRM2_HUMAN	tRNA (uracil-5-)-methyltransferase homolog	0		
HSPB6_HUMAN	Heat shock protein beta-6	0		
K1C16_HUMAN	Keratin, type I cytoskeletal 16	0		
UGDH_HUMAN	UDP-glucose 6-dehydrogenase	0	Cyto	nucleotide binding,oxidoreductase activity

Selected localization and biological function of TGF-β1-induced secretome were extracted from swissprot keywords and GO terms

*ext., extracellular; cyt., cytosol; ER, edoplasmic reticulum; nuc., nucleus; rib., ribosome; mem., membrane; lyso., lysosome; intra., intracellular; mito., mitochondria

§TM: Number of transmembrane domain
