# Supplementary material for: Proteomic Identification of ADAM12 as a Regulator for TGF-β1-Induced Differentiation of Human Mesenchymal Stem Cells to Smooth Muscle Cells
Source: PLoS One. 2012 Jul 13;7(7):e40820. doi: 10.1371/journal.pone.0040820 (PMC3396647; doi:10.1371/journal.pone.0040820)
Supplement: Table S3 — Functional annotation of up-regulated proteins by TGF-β1. (PDF) [file pone.0040820.s005.pdf]

Table S3: Functional annotation of up-regulated proteins by TGF-β1

Category	GO Term	Count	%	adj. p-value
BP	intracellular transport	33	14.2241379	1.34E-06
BP	translational elongation	14	6.03448276	3.30E-06
BP	protein transport	35	15.0862069	3.58E-06
BP	establishment of protein localization	35	15.0862069	4.51E-06
BP	intracellular protein transport	24	10.3448276	5.71E-06
BP	cellular protein localization	25	10.7758621	6.80E-06
BP	cellular macromolecule localization	25	10.7758621	7.82E-06
BP	protein localization	37	15.9482759	1.29E-05
BP	vesicle-mediated transport	27	11.637931	1.61E-04
BP	translation	19	8.18965517	6.20E-03
BP	cell adhesion	27	11.637931	1.08E-02
BP	biological adhesion	27	11.637931	1.11E-02
CC	endoplasmic reticulum	67	28.8793103	1.95E-20
CC	endoplasmic reticulum part	36	15.5172414	1.42E-14
CC	endomembrane system	48	20.6896552	3.18E-11
CC	organelle membrane	56	24.137931	2.00E-10
CC	endoplasmic reticulum membrane	25	10.7758621	3.74E-08
CC	nuclear envelope-endoplasmic reticulum network	25	10.7758621	1.16E-07
CC	endoplasmic reticulum lumen	12	5.17241379	1.27E-04
CC	membrane fraction	35	15.0862069	1.64E-03
CC	intrinsic to membrane	131	56.4655172	1.80E-03
CC	insoluble fraction	35	15.0862069	3.73E-03
CC	cytoplasmic membrane-bounded vesicle	27	11.637931	3.78E-03
CC	integral to membrane	126	54.3103448	5.56E-03
CC	membrane-bounded vesicle	27	11.637931	6.76E-03
CC	cytosolic ribosome	10	4.31034483	1.43E-02
CC	cytoplasmic vesicle	28	12.0689655	1.93E-02
CC	pigment granule	10	4.31034483	2.53E-02
CC	melanosome	10	4.31034483	2.53E-02
CC	cell fraction	39	16.8103448	3.03E-02
CC	vesicle	28	12.0689655	4.07E-02
MF	structural constituent of ribosome	14	6.03448276	3.10E-03
MF	SNAP receptor activity	6	2.5862069	8.91E-03

Count: No. of protein annotated to corresponding GO term

%: percentage of Count to total no. of TGF-β1-induced proteins

adj. p-value: adjusted p-value by multiple testing correction technique (Benjamini)

Category: CC, Cellular component; MF, Molecular function; BP, Biological process
