# Supplementary material for: Proteomic Identification of ADAM12 as a Regulator for TGF-β1-Induced Differentiation of Human Mesenchymal Stem Cells to Smooth Muscle Cells
Source: PLoS One. 2012 Jul 13;7(7):e40820. doi: 10.1371/journal.pone.0040820 (PMC3396647; doi:10.1371/journal.pone.0040820)
Supplement: Table S4 — Comparison of lipid raft proteome of hASCs with a database for lipid raft porteins (Zhang, T. et al. (2010) J. Mol. Biol., 402: 761–773). (PDF) [file pone.0040820.s006.pdf]

Table S4: Comparison of lipid raft proteome of hASCs with a database for lipid raft proteins (Zhang, T. et al. (2010) J. Mol. Biol., 402: 761-773)

Gene	Protein	Log2	Database
ADAM12	Disintegrin and metalloproteinase domain-containing protein 12	6.233	Lipid rafts
LDLR	Low-density lipoprotein receptor	4.732	Lipid rafts
PKD2	Polycystin-2	4.435	Lipid rafts
SDC1	Syndecan-1	3.579	Lipid rafts
FAS	Tumor necrosis factor receptor superfamily member 6	3.355	Lipid rafts
CTNNB1	Catenin beta-1	2.448	Lipid rafts
ADAM10	ADAM10 protein	2.378	Lipid rafts
KIDINS220	Kinase D-interacting substrate of 220 kDa	2.181	Lipid rafts
DYSF	Dysferlin	1.879	Lipid rafts
CD99	CD99 antigen	1.588	Lipid rafts
ATP1B1	Sodium/potassium-transporting ATPase subunit beta-1	1.397	Lipid rafts
NCSTN	Nicastrin	1.300	Lipid rafts
ITGAV	ITGAV protein	1.027	Lipid rafts
CALR	Calreticulin	1.022	Lipid rafts
CD47	Leukocyte surface antigen CD47	1.006	Lipid rafts
RAP1A	Ras-related protein Rap-1A	1.003	Lipid rafts
CANX	Calnexin	0.991	Lipid rafts
ITGB1	Integrin beta-1	0.986	Lipid rafts
GNAS	Guanine nucleotide-binding protein G(s) subunit alpha isoforms XLas	0.899	Lipid rafts
ALCAM	CD166 antigen	0.879	Lipid rafts
ADAM17	Disintegrin and metalloproteinase domain-containing protein 17	0.758	Lipid rafts
FLOT1	Flotillin-1	0.700	Lipid rafts
TFRC	Transferrin receptor protein 1	0.699	Lipid rafts
PALM	Paralemmin	0.589	Lipid rafts
HRAS	GTPase HRas	0.589	Lipid rafts
DHCR24	24-dehydrocholesterol reductase	0.583	Lipid rafts
LRP1	Prolow-density lipoprotein receptor-related protein 1	0.535	Lipid rafts
CTSB	Cathepsin B	0.535	Lipid rafts
RHOG	Rho-related GTP-binding protein RhoG	0.504	Lipid rafts
CD63	CD63 antigen	0.451	Lipid rafts
RRAS	Ras-related protein R-Ras	0.416	Lipid rafts
CACNA2D1	Voltage-dependent calcium channel subunit alpha-2/delta-1	0.416	Lipid rafts
HK1	Hexokinase-1	0.410	Lipid rafts
ARF6	ADP-ribosylation factor 6	0.394	Lipid rafts
SNAP23	Synaptosomal-associated protein 23	0.335	Lipid rafts
ITGA2	Integrin alpha-2	0.315	Lipid rafts
THY1	Thy-1 membrane glycoprotein	0.313	Lipid rafts
GNAI2	Guanine nucleotide-binding protein G(i), alpha-2 subunit	0.309	Lipid rafts
ATP2B1	Plasma membrane calcium-transporting ATPase 1	0.308	Lipid rafts
GNA11	Guanine nucleotide-binding protein subunit alpha-11	0.275	Lipid rafts
PHB	Prohibitin	0.254	Lipid rafts
ACTB	Actin, cytoplasmic 1	0.220	Lipid rafts
BSG	Basigin	0.210	Lipid rafts
GNAQ	Guanine nucleotide-binding protein G(q) subunit alpha	0.201	Lipid rafts
ATP1A1	Sodium/potassium-transporting ATPase subunit alpha-1	0.190	Lipid rafts
STX4	Syntaxin-4	0.159	Lipid rafts
GNB1	Guanine nucleotide-binding protein G(I)/G(S)/G(T) subunit beta-1	0.137	Lipid rafts
PDGFRB	Beta-type platelet-derived growth factor receptor	0.123	Lipid rafts
DAG1	Dystroglycan	0.110	Lipid rafts
CD59	CD59 glycoprotein	0.065	Lipid rafts
ITGA3	Integrin alpha-3	0.014	Lipid rafts
FLNA	Filamin-A	-0.034	Lipid rafts

ANXA6	Annexin A6	-0.088	Lipid rafts
CALM1	Calmodulin	-0.126	Lipid rafts
NRAS	GTPase NRas	-0.130	Lipid rafts
ATP2B4	Plasma membrane calcium-transporting ATPase 4	-0.147	Lipid rafts
TMED2	Transmembrane emp24 domain-containing protein 2	-0.185	Lipid rafts
VDAC1	Voltage-dependent anion-selective channel protein 1	-0.196	Lipid rafts
RTN4	RTN4 isoform B1	-0.219	Lipid rafts
STIM1	Stromal interaction molecule 1	-0.263	Lipid rafts
CD44	CD44 antigen	-0.284	Lipid rafts
VAMP3	Vesicle-associated membrane protein 3	-0.290	Lipid rafts
BSG	Cervical EMMPRIN	-0.312	Lipid rafts
ANXA2	Annexin A2	-0.326	Lipid rafts
FLOT2	Flotillin-2	-0.332	Lipid rafts
RAB5A	Ras-related protein Rab-5A	-0.371	Lipid rafts
CD9	CD9 antigen	-0.376	Lipid rafts
RAB18	Ras-related protein Rab-18	-0.376	Lipid rafts
MMP2	72 kDa type IV collagenase	-0.381	Lipid rafts
ICAM1	Intercellular adhesion molecule 1	-0.392	Lipid rafts
RHOA	Transforming protein RhoA	-0.446	Lipid rafts
CDC42	cDNA FLJ55107, highly similar to Cell division control protein 42 homolog	-0.476	Lipid rafts
MARCKS	Myristoylated alanine-rich C-kinase substrate	-0.492	Lipid rafts
ITGA5	Integrin alpha-5	-0.547	Lipid rafts
RP2	Protein XRP2	-0.556	Lipid rafts
PRKCA	Protein kinase C alpha type	-0.736	Lipid rafts
PDGFRA	Alpha-type platelet-derived growth factor receptor	-0.765	Lipid rafts
EGFR	Epidermal growth factor receptor	-0.790	Lipid rafts
STOM	Erythrocyte band 7 integral membrane protein	-0.941	Lipid rafts
CAV2	Caveolin-2	-0.987	Lipid rafts
HSPA9	Stress-70 protein, mitochondrial	-1.014	Lipid rafts
NRP1	Neuropilin-1	-1.083	Lipid rafts
VCL	Vinculin	-1.170	Lipid rafts
RAP2A	Ras-related protein Rap-2a	-1.213	Lipid rafts
ARF1	ADP-ribosylation factor 1	-1.366	Lipid rafts
PTGIS	Prostacyclin synthase	-1.412	Lipid rafts
RFTN1	Raftlin	-1.487	Lipid rafts
MSN	Moesin	-1.553	Lipid rafts
LGALS1	Galectin-1	-1.600	Lipid rafts
CD81	CD81 antigen	-1.621	Lipid rafts
CAV1	Caveolin-1	-1.638	Lipid rafts
IQGAP1	Ras GTPase-activating-like protein IQGAP1	-1.685	Lipid rafts
CAPN1	Calpain-1 catalytic subunit	-1.983	Lipid rafts
TNS1	TNS1 protein (Fragment)	-2.294	Lipid rafts
CAPNS1	Calpain small subunit 1	-2.614	Lipid rafts
CAPN2	Calpain-2 catalytic subunit	-3.204	Lipid rafts
ACE	Angiotensin-converting enzyme	-4.195	Lipid rafts
PVR	Poliovirus receptor	5.530	Non-lipid rafts
SLC1A4	Neutral amino acid transporter A	5.161	Non-lipid rafts
SEMA7A	Semaphorin-7A	4.296	Non-lipid rafts
SGPL1	Sphingosine-1-phosphate lyase 1	4.214	Non-lipid rafts
SLC27A4	Long-chain fatty acid transport protein 4	4.090	Non-lipid rafts
RPL29	60S ribosomal protein L29	3.899	Non-lipid rafts
PVRL2	Poliovirus receptor-related protein 2	3.735	Non-lipid rafts
LRRC15	HCG2043616	3.603	Non-lipid rafts
PTPRM	Receptor-type tyrosine-protein phosphatase mu	3.438	Non-lipid rafts
AP2M1	AP-2 complex subunit mu	3.431	Non-lipid rafts

FZD7	Frizzled-7	3.292	Non-lipid rafts
MCAM	Cell surface glycoprotein MUC18	3.123	Non-lipid rafts
SLC4A7	Solute carrier family 4 sodium bicarbonate cotransporter member 7	3.074	Non-lipid rafts
HDLBP	Vigilin	2.761	Non-lipid rafts
PLCE1	1-phosphatidylinositol-4,5-bisphosphate phosphodiesterase epsilon-1	2.726	Non-lipid rafts
SCAMP3	Secretory carrier-associated membrane protein 3	2.679	Non-lipid rafts
AP2B1	AP-2 complex subunit beta	2.603	Non-lipid rafts
ENG	Endoglin	2.423	Non-lipid rafts
PTK7	Tyrosine-protein kinase-like 7	2.245	Non-lipid rafts
SLC3A2	4F2 cell-surface antigen heavy chain	2.218	Non-lipid rafts
STX2	Syntaxin-2	2.175	Non-lipid rafts
SIRPA	Tyrosine-protein phosphatase non-receptor type substrate 1	2.128	Non-lipid rafts
SLC12A4	Solute carrier family 12 member 4	2.109	Non-lipid rafts
ODZ3	Teneurin-3	2.015	Non-lipid rafts
RAB31	Ras-related protein Rab-31	1.962	Non-lipid rafts
PPFIBP1	Liprin-beta-1	1.890	Non-lipid rafts
AP2A2	AP-2 complex subunit alpha-2	1.882	Non-lipid rafts
ITGA1	Integrin alpha-1	1.857	Non-lipid rafts
MFGE8	Lactadherin	1.847	Non-lipid rafts
SLC1A5	Neutral amino acid transporter B(0)	1.844	Non-lipid rafts
PICALM	Phosphatidylinositol-binding clathrin assembly protein	1.834	Non-lipid rafts
POR	NADPH--cytochrome P450 reductase	1.815	Non-lipid rafts
AP2A1	AP-2 complex subunit alpha-1	1.735	Non-lipid rafts
FAM38A	Protein FAM38A	1.693	Non-lipid rafts
NCLN	Nicalin	1.661	Non-lipid rafts
IGF2R	Cation-independent mannose-6-phosphate receptor	1.545	Non-lipid rafts
ITGA11	Integrin alpha-11	1.541	Non-lipid rafts
PDIA4	Protein disulfide-isomerase A4	1.490	Non-lipid rafts
SLC25A4	ADP/ATP translocase 1	1.420	Non-lipid rafts
EPHB3	Ephrin type-B receptor 3	1.393	Non-lipid rafts
NUCB2	Nucleobindin-2	1.385	Non-lipid rafts
CSPG4	Chondroitin sulfate proteoglycan 4	1.374	Non-lipid rafts
STX12	Syntaxin-12	1.365	Non-lipid rafts
ATP6AP2	Renin receptor	1.330	Non-lipid rafts
SLC9A1	Sodium/hydrogen exchanger 1	1.315	Non-lipid rafts
EPHA7	Ephrin type-A receptor 7	1.299	Non-lipid rafts
CDH2	Cadherin-2	1.295	Non-lipid rafts
RAB6A	Ras-related protein Rab-6A	1.234	Non-lipid rafts
SC4MOL	C-4 methylsterol oxidase	1.213	Non-lipid rafts
PARVA	Alpha-parvin	1.179	Non-lipid rafts
STT3A	Dolichyl-diphosphooligosaccharide--protein glycosyltransferase subunit STT3A	1.136	Non-lipid rafts
MTDH	Protein LYRIC	1.099	Non-lipid rafts
GNB2L1	Guanine nucleotide-binding protein subunit beta-2-like 1	1.042	Non-lipid rafts
UTRN	Utrophin	1.028	Non-lipid rafts
CKAP4	Cytoskeleton-associated protein 4	1.002	Non-lipid rafts
SERPINH1	Serpin H1	0.997	Non-lipid rafts
GJA1	Gap junction alpha-1 protein	0.971	Non-lipid rafts
LAMP1	Lysosome-associated membrane glycoprotein 1	0.954	Non-lipid rafts
ARF4	ADP-ribosylation factor 4	0.944	Non-lipid rafts
RAB8B	Ras-related protein Rab-8B	0.940	Non-lipid rafts
RAP1B	Ras-related protein Rap-1b	0.919	Non-lipid rafts
CTNNA1	Catenin alpha-1	0.916	Non-lipid rafts
TMEM33	Transmembrane protein 33	0.909	Non-lipid rafts
GLG1	Golgi apparatus protein 1	0.897	Non-lipid rafts
TXNDC5	Thioredoxin domain-containing protein 5	0.892	Non-lipid rafts

ITGB5	Integrin beta-5	0.854	Non-lipid rafts
EEF1G	Elongation factor 1-gamma	0.788	Non-lipid rafts
BST1	ADP-ribosyl cyclase 2	0.781	Non-lipid rafts
CHP	Calcium-binding protein p22	0.779	Non-lipid rafts
HSPA5	78 kDa glucose-regulated protein	0.761	Non-lipid rafts
RAB8A	Ras-related protein Rab-8A	0.757	Non-lipid rafts
LAMP2	Lysosome-associated membrane glycoprotein 2	0.731	Non-lipid rafts
ENPP2	Ectonucleotide pyrophosphatase/phosphodiesterase family member 2	0.729	Non-lipid rafts
CLTC	Clathrin heavy chain 1	0.700	Non-lipid rafts
KIRREL	Kin of IRRE-like protein 1	0.697	Non-lipid rafts
RAB11B	Ras-related protein Rab-11B	0.694	Non-lipid rafts
RRAS2	Ras-related protein R-Ras2	0.680	Non-lipid rafts
SCFD1	Sec1 family domain-containing protein 1	0.673	Non-lipid rafts
MYO1C	Myosin-Ic	0.669	Non-lipid rafts
LETM1	LETM1 and EF-hand domain-containing protein 1, mitochondrial	0.650	Non-lipid rafts
GNA13	Guanine nucleotide-binding protein subunit alpha-13	0.641	Non-lipid rafts
STOML2	Stomatin-like protein 2	0.638	Non-lipid rafts
PDIA3	Protein disulfide-isomerase A3	0.619	Non-lipid rafts
TPBG	Trophoblast glycoprotein	0.573	Non-lipid rafts
VAPB	Vesicle-associated membrane protein-associated protein B/C	0.566	Non-lipid rafts
PGRMC2	Membrane-associated progesterone receptor component 2	0.510	Non-lipid rafts
HLA-B	MHC class I antigen (Fragment)	0.482	Non-lipid rafts
GNAI3	Guanine nucleotide-binding protein G(k) subunit alpha	0.430	Non-lipid rafts
FAP	Fibroblast activation protein, alpha, isoform CRA_a	0.390	Non-lipid rafts
GPC6	Glypican-6	0.379	Non-lipid rafts
CYB5R3	NADH-cytochrome b5 reductase 3	0.373	Non-lipid rafts
DPM1	Dolichol-phosphate mannosyltransferase	0.323	Non-lipid rafts
GNAI1	Guanine nucleotide-binding protein G(i), alpha-1 subunit	0.312	Non-lipid rafts
ABCC1	Multidrug resistance-associated protein 1	0.294	Non-lipid rafts
RALA	Ras-related protein Ral-A	0.288	Non-lipid rafts
RASGRF1	Ras-specific guanine nucleotide-releasing factor 1	0.261	Non-lipid rafts
MYO1B	Myosin-Ib	0.255	Non-lipid rafts
LRRC32	Leucine-rich repeat-containing protein 32	0.249	Non-lipid rafts
TRPM4	Transient receptor potential cation channel subfamily M member 4	0.240	Non-lipid rafts
TM9SF3	Transmembrane 9 superfamily member 3	0.237	Non-lipid rafts
SGCD	Delta-sarcoglycan	0.234	Non-lipid rafts
EPHA2	Ephrin type-A receptor 2	0.188	Non-lipid rafts
TOLLIP	Toll-interacting protein	0.156	Non-lipid rafts
OPRS1	Sigma 1-type opioid receptor	0.142	Non-lipid rafts
ATP5B	ATP synthase subunit beta, mitochondrial	0.107	Non-lipid rafts
SMPD4	Sphingomyelin phosphodiesterase 4	0.098	Non-lipid rafts
SLC39A14	Zinc transporter ZIP14	0.077	Non-lipid rafts
SGCB	Beta-sarcoglycan	0.055	Non-lipid rafts
PRKAR2A	cAMP-dependent protein kinase type II-alpha regulatory subunit	0.049	Non-lipid rafts
CALD1	Caldesmon	0.043	Non-lipid rafts
KRAS	GTPase KRas	0.033	Non-lipid rafts
ENPP1	Ectonucleotide pyrophosphatase/phosphodiesterase family member 1	0.028	Non-lipid rafts
CDH11	Cadherin-11	0.026	Non-lipid rafts
ERLIN1	Erlin-1	-0.012	Non-lipid rafts
RAB32	Ras-related protein Rab-32	-0.022	Non-lipid rafts
DCBLD2	Discoidin, CUB and LCCL domain-containing protein 2	-0.023	Non-lipid rafts
ITM2B	Integral membrane protein 2B	-0.026	Non-lipid rafts
PPIB	Peptidyl-prolyl cis-trans isomerase B	-0.033	Non-lipid rafts
PLXNB2	Plexin-B2	-0.035	Non-lipid rafts
PON2	Serum paraoxonase/arylesterase 2	-0.069	Non-lipid rafts
PANX1	Pannexin-1	-0.080	Non-lipid rafts

EMD	Emerin	-0.114	Non-lipid rafts
SLC12A2	Solute carrier family 12 member 2	-0.125	Non-lipid rafts
BASP1	Brain acid soluble protein 1	-0.135	Non-lipid rafts
LPHN2	Latrophilin-2	-0.144	Non-lipid rafts
GNB4	Guanine nucleotide-binding protein subunit beta-4	-0.158	Non-lipid rafts
CD276	CD276 antigen	-0.160	Non-lipid rafts
HLA-C	HLA class I histocompatibility antigen, Cw-12 alpha chain	-0.204	Non-lipid rafts
EPB41L2	Band 4.1-like protein 2	-0.214	Non-lipid rafts
TRPV2	Transient receptor potential cation channel subfamily V member 2	-0.216	Non-lipid rafts
S100A11	Protein S100-A11	-0.259	Non-lipid rafts
GDI2	Rab GDP dissociation inhibitor beta	-0.289	Non-lipid rafts
TMED10	Transmembrane emp24 domain-containing protein 10	-0.301	Non-lipid rafts
RAB5B	Ras-related protein Rab-5B	-0.304	Non-lipid rafts
VAPA	Vesicle-associated membrane protein-associated protein A	-0.320	Non-lipid rafts
ARL6IP1	ADP-ribosylation factor-like protein 6-interacting protein 1	-0.321	Non-lipid rafts
PLXND1	Plexin-D1	-0.329	Non-lipid rafts
NPTN	Neuroplastin	-0.330	Non-lipid rafts
SCRIB	Protein scribble homolog	-0.340	Non-lipid rafts
PPP1R12A	Protein phosphatase 1 regulatory subunit 12A	-0.340	Non-lipid rafts
SFXN1	Sideroflexin-1	-0.376	Non-lipid rafts
AHNAK	Neuroblast differentiation-associated protein AHNAK	-0.422	Non-lipid rafts
SYPL1	Synaptophysin-like protein 1	-0.436	Non-lipid rafts
ATP1B3	Sodium/potassium-transporting ATPase subunit beta-3	-0.451	Non-lipid rafts
HSPB1	Heat shock protein beta-1	-0.453	Non-lipid rafts
NPR2	Atrial natriuretic peptide receptor B	-0.456	Non-lipid rafts
NOTCH2	Neurogenic locus notch homolog protein 2	-0.460	Non-lipid rafts
RAB34	Ras-related protein Rab-34	-0.476	Non-lipid rafts
SCARB2	Lysosome membrane protein 2	-0.478	Non-lipid rafts
ITGA4	Integrin alpha-4	-0.483	Non-lipid rafts
HAS1	Hyaluronan synthase 1	-0.483	Non-lipid rafts
FSCN1	Fascin	-0.485	Non-lipid rafts
HLA-B	MHC class I antigen (Fragment)	-0.491	Non-lipid rafts
RAB13	Ras-related protein Rab-13	-0.532	Non-lipid rafts
HLA-B	MHC class I antigen (Fragment)	-0.533	Non-lipid rafts
EPB41L1	cDNA FLJ39529 fis, clone PUAEN2004067, highly similar to Band 4.1-like protein 1	-0.556	Non-lipid rafts
VASN	Vasorin	-0.575	Non-lipid rafts
PLXNA1	Plexin-A1	-0.586	Non-lipid rafts
PTPRD	Receptor-type tyrosine-protein phosphatase delta	-0.595	Non-lipid rafts
MRC2	C-type mannose receptor 2	-0.624	Non-lipid rafts
ALPL	Alkaline phosphatase, tissue-nonspecific isozyme	-0.627	Non-lipid rafts
LANCL1	LanC-like protein 1	-0.639	Non-lipid rafts
RDX	Radixin	-0.651	Non-lipid rafts
HLA-A	MHC class I antigen (Fragment)	-0.662	Non-lipid rafts
RECK	Reversion-inducing cysteine-rich protein with Kazal motifs	-0.663	Non-lipid rafts
DYNC1H1	Cytoplasmic dynein 1 heavy chain 1	-0.667	Non-lipid rafts
NME2	Nucleoside diphosphate kinase	-0.707	Non-lipid rafts
HLA-B	HLA class I histocompatibility antigen, B-58 alpha chain	-0.722	Non-lipid rafts
CDH13	cDNA FLJ52398, highly similar to Cadherin-13	-0.756	Non-lipid rafts
ARF5	ADP-ribosylation factor 5	-0.758	Non-lipid rafts
ANPEP	Aminopeptidase N	-0.759	Non-lipid rafts
YWHAZ	14-3-3 protein zeta/delta	-0.761	Non-lipid rafts
PLEC1	Plectin-1	-0.780	Non-lipid rafts
RAP2B	Ras-related protein Rap-2b	-0.788	Non-lipid rafts
MMP14	Matrix metalloproteinase-14	-0.805	Non-lipid rafts
TMEM161A	Transmembrane protein 161A	-0.824	Non-lipid rafts

TMED1	Transmembrane emp24 domain-containing protein 1	-0.832	Non-lipid rafts
TLN1	Talin-1	-0.875	Non-lipid rafts
HLA-A	MHC class I antigen (Fragment)	-0.893	Non-lipid rafts
CNTNAP1	Contactin-associated protein 1	-0.908	Non-lipid rafts
DDR2	Discoidin domain-containing receptor 2	-0.934	Non-lipid rafts
HLA-A	MHC class I antigen (Fragment)	-0.955	Non-lipid rafts
HLA-A	MHC class I antigen	-0.972	Non-lipid rafts
HLA-A	MHC class I antigen (Fragment)	-0.974	Non-lipid rafts
ANXA1	Annexin A1	-0.984	Non-lipid rafts
PON2	PON2 protein	-1.034	Non-lipid rafts
EHD1	EH domain-containing protein 1	-1.045	Non-lipid rafts
SGCE	Epsilon-sarcoglycan	-1.080	Non-lipid rafts
EPB41L3	cDNA FLJ77757	-1.089	Non-lipid rafts
EHD2	EH domain-containing protein 2	-1.186	Non-lipid rafts
S100A10	Protein S100-A10	-1.203	Non-lipid rafts
FLNB	Filamin-B	-1.302	Non-lipid rafts
CNP	2~,3~-cyclic-nucleotide 3~-phosphodiesterase	-1.355	Non-lipid rafts
NT5E	5~-nucleotidase	-1.367	Non-lipid rafts
IFITM1	Interferon induced transmembrane protein 1 (9-27)	-1.512	Non-lipid rafts
LRPAP1	Alpha-2-macroglobulin receptor-associated protein	-1.568	Non-lipid rafts
ARF3	ADP-ribosylation factor 3	-1.642	Non-lipid rafts
ARL6IP5	PRA1 family protein 3	-1.645	Non-lipid rafts
ANXA5	Annexin A5	-1.666	Non-lipid rafts
ENO1	Alpha-enolase	-1.693	Non-lipid rafts
AKR1A1	Alcohol dehydrogenase [NADP+]	-1.761	Non-lipid rafts
PROCR	Endothelial protein C receptor	-1.958	Non-lipid rafts
CTTN	Src substrate cortactin	-2.020	Non-lipid rafts
CD109	CD109 antigen	-2.065	Non-lipid rafts
HSPA4	Heat shock 70 kDa protein 4	-2.190	Non-lipid rafts
ISLR	Immunoglobulin superfamily containing leucine-rich repeat protein	-2.210	Non-lipid rafts
CFL1	Cofilin-1	-2.240	Non-lipid rafts
KRT1	Keratin, type II cytoskeletal 1	-2.297	Non-lipid rafts
PPAP2B	Lipid phosphate phosphohydrolase 3	-2.325	Non-lipid rafts
ABCC4	Multidrug resistance-associated protein 4	-2.332	Non-lipid rafts
MME	Neprilysin	-2.336	Non-lipid rafts
ARPC1B	Actin-related protein 2/3 complex subunit 1B	-2.346	Non-lipid rafts
CLIC1	Chloride intracellular channel protein 1	-2.439	Non-lipid rafts
TIAM1	T-lymphoma invasion and metastasis-inducing protein 1	-2.507	Non-lipid rafts
SLC39A7	Zinc transporter SLC39A7	-2.569	Non-lipid rafts
TAP1	Antigen peptide transporter 1	-2.728	Non-lipid rafts
SPTBN1	Spectrin beta chain, brain 1	-3.233	Non-lipid rafts
ADD3	Gamma-adducin	-3.344	Non-lipid rafts
SPTAN1	Spectrin alpha chain, brain	-3.521	Non-lipid rafts
MAP1B	Microtubule-associated protein 1B	-3.747	Non-lipid rafts
DPP4	Dipeptidyl peptidase 4	-4.834	Non-lipid rafts
AKAP12	A-kinase anchor protein 12	-5.017	Non-lipid rafts
PLOD2	Procollagen-lysine,2-oxoglutarate 5-dioxygenase 2	5.699	
ALG2	Alpha-1,3-mannosyltransferase ALG2	5.195	
SCD	Acyl-CoA desaturase	5.166	
HYOU1	Hypoxia up-regulated protein 1	5.060	
FADS2	Fatty acid desaturase 2	4.978	
ATP5I	ATP synthase subunit e, mitochondrial	4.970	
IDH2	Isocitrate dehydrogenase [NADP], mitochondrial	4.905	
P4HA2	Prolyl 4-hydroxylase subunit alpha-2	4.896	
RPS15	40S ribosomal protein S15	4.787	

KRT72	cDNA FLJ50908, highly similar to Homo sapiens keratin protein K6irs (K6IRS2), mRNA	4.629
RPS15A	40S ribosomal protein S15a	4.595
PRKDC	DNA-dependent protein kinase catalytic subunit	4.583
PTGS1	Prostaglandin G/H synthase 1	4.566
FNDC3B	Fibronectin type III domain-containing protein 3B	4.468
TGFBI	Transforming growth factor-beta-induced protein ig-h3	4.266
AFG3	Similar to AFG3 ATPase family gene 3-like 2 (Yeast) (Fragment)	4.033
ANKLE2	Ankyrin repeat and LEM domain-containing protein 2	3.808
TMX1	Thioredoxin-related transmembrane protein 1	3.720
FKBP9	FK506-binding protein 9	3.694
COMP	Cartilage oligomeric matrix protein	3.680
RPS27L	40S ribosomal protein S27-like protein	3.639
GLT25D1	Procollagen galactosyltransferase 1	3.625
LEMD3	Inner nuclear membrane protein Man1	3.616
ERG1	Squalene epoxidase	3.604
LAMB1	Laminin subunit beta-1	3.567
SULT1C3	Sulfotransferase 1C3	3.517
TENC1	Tensin-like C1 domain-containing phosphatase	3.500
PLOD1	Procollagen-lysine,2-oxoglutarate 5-dioxygenase 1	3.445
OSBPL5	Oxysterol-binding protein-related protein 5	3.363
ERP29	Endoplasmic reticulum protein ERp29	3.296
HNRNPC	Heterogeneous nuclear ribonucleoproteins C1/C2	3.290
PIGK	GPI-anchor transamidase	3.268
MAN1B1	Endoplasmic reticulum mannosyl-oligosaccharide 1,2-alpha-mannosidase	3.253
GOSR2	Golgi SNAP receptor complex member 2	3.196
VPS45	Vacuolar protein sorting-associated protein 45	3.169
ATP6V0A1	V-type proton ATPase 116 kDa subunit a isoform 1	3.136
SRPR	Signal recognition particle receptor subunit alpha	3.112
OSBPL8	Oxysterol-binding protein-related protein 8	3.098
SPARC	SPARC	3.093
MIA3	Melanoma inhibitory activity protein 3	3.082
COL12A1	Collagen alpha-1(XII) chain	3.068
IMPAD1	Inositol monophosphatase 3	3.008
FAT1	Protocadherin Fat 1	2.952
ALDH1B1	Aldehyde dehydrogenase X, mitochondrial	2.920
HNRNPM	Heterogeneous nuclear ribonucleoprotein M	2.851
BET1L	BET1-like protein	2.816
FN1	Fibronectin	2.776
DERL1	Derlin-1	2.746
USMG5	Up-regulated during skeletal muscle growth protein 5	2.726
FAM177A1	Protein FAM177A1	2.677
TIMM50	Mitochondrial import inner membrane translocase subunit TIM50	2.628
CALU	Calumenin	2.595
FAM134C	Protein FAM134C	2.585
THBS1	Thrombospondin-1	2.566
EIF4A1	Eukaryotic initiation factor 4A-I	2.526
EEF1D	EEF1D protein (Fragment)	2.522
UBXN4	UBX domain-containing protein 4	2.505
CPT1A	Carnitine O-palmitoyltransferase 1, liver isoform	2.491
SLC38A10	Putative sodium-coupled neutral amino acid transporter 10	2.484
RPL36	60S ribosomal protein L36	2.471
MBOAT7	Lysophospholipid acyltransferase 7	2.463
RCN3	Reticulocalbin-3	2.462
STX18	Syntaxin-18	2.450

P4HA1	Prolyl 4-hydroxylase subunit alpha-1	2.437
DYNLL1	Dynein light chain 1, cytoplasmic	2.340
KIAA0220L	Putative NPIP-like protein KIAA0220-like	2.293
CHST14	Carbohydrate sulfotransferase 14	2.288
FIS1	Mitochondrial fission 1 protein	2.238
SSR2	Translocon-associated protein subunit beta	2.195
RAB22A	Ras-related protein Rab-22A	2.183
CLPTM1	Cleft lip and palate transmembrane protein 1	2.160
UGP2	UTP--glucose-1-phosphate uridylyltransferase	2.157
KIAA0776	UPF0555 protein KIAA0776	2.147
RPL19	60S ribosomal protein L19	2.116
SC65	Synaptonemal complex protein SC65	2.087
PXDN	Peroxidasin homolog	2.051
KIAA0090	Uncharacterized protein KIAA0090	2.036
APOL2	Apolipoprotein L2	2.020
SEC63	Translocation protein SEC63 homolog	2.007
CCDC47	Coiled-coil domain-containing protein 47	1.983
RPL27A	60S ribosomal protein L27a	1.969
C7orf59	UPF0539 protein C7orf59	1.963
GOSR1	Golgi SNAP receptor complex member 1	1.892
DERL2	Derlin-2	1.890
GALNT2	Polypeptide N-acetylgalactosaminyltransferase 2	1.878
TPM1	Tropomyosin alpha-1 chain	1.866
ACSL3	Long-chain-fatty-acid--CoA ligase 3	1.845
BTN2A1	Butyrophilin subfamily 2 member A1	1.833
YIPF5	Protein YIPF5	1.760
LIMA1	LIM domain and actin-binding protein 1	1.758
INF2	Inverted formin-2	1.728
MICAL2	Protein MICAL-2	1.727
RDH11	Retinol dehydrogenase 11	1.725
GNG5	Guanine nucleotide-binding protein G(I)/G(S)/G(O) subunit gamma-5	1.718
B2M	Beta-2-microglobulin	1.702
SAR1B	GTP-binding protein SAR1b	1.701
LPGAT1	Acyl-CoA:lysophosphatidylglycerol acyltransferase 1	1.700
PI16	Peptidase inhibitor 16	1.676
MAN2A1	Alpha-mannosidase 2	1.669
EIF2S1	Eukaryotic translation initiation factor 2 subunit 1	1.655
SEC61B	Protein transport protein Sec61 subunit beta	1.646
DHCR7	7-dehydrocholesterol reductase	1.634
KTN1	Kinectin	1.619
VANGL1	Vang-like protein 1	1.603
RRBP1	Ribosome-binding protein 1	1.596
TM9SF4	Transmembrane 9 superfamily member 4	1.570
GLT8D1	Glycosyltransferase 8 domain-containing protein 1	1.562
SPTLC1	Serine palmitoyltransferase 1	1.562
P116	cDNA, FLJ93202, Homo sapiens protease inhibitor 16 (PI16), mRNA	1.553
FAF2	FAS-associated factor 2	1.552
FDFT1	Squalene synthetase	1.511
COL1A2	Collagen alpha-2(I) chain	1.486
ITFG3	Protein ITFG3	1.457
TMEM165	Transmembrane protein 165	1.449
SSR1	Translocon-associated protein subunit alpha	1.447
TPM2b	Beta tropomyosin isoform	1.424
CTNND1	Catenin delta-1	1.414
PTRH2	Peptidyl-tRNA hydrolase 2, mitochondrial	1.398
C15orf24	UPF0480 protein C15orf24	1.397

RPL32	60S ribosomal protein L32	1.383
ERP44	Endoplasmic reticulum resident protein ERp44	1.377
ATP2A2	Sarcoplasmic/endoplasmic reticulum calcium ATPase 2	1.362
COMT	Catechol O-methyltransferase	1.351
SRPRB	Signal recognition particle receptor subunit beta	1.341
SLC44A2	Choline transporter-like protein 2	1.337
NUCB1	Nucleobindin-1	1.333
TPM2	Tropomyosin 2 (Beta)	1.321
NPM1	Nucleophosmin	1.318
LMF2	Lipase maturation factor 2	1.312
H2AFV	Histone H2A.V	1.302
H2AFJ	Histone H2A.J	1.300
RPL27	60S ribosomal protein L27	1.298
SURF4	Surfeit locus protein 4	1.298
RAB21	Ras-related protein Rab-21	1.286
STX7	Syntaxin-7	1.269
LEPREL2	Prolyl 3-hydroxylase 3	1.267
NOMO1	Nodal modulator 1	1.250
H2BFS	Histone H2B type F-S	1.233
SSR4	Translocon-associated protein subunit delta	1.217
HIST2H2AA3	Histone H2A type 2-A	1.214
LPLUNC3	Long palate, lung and nasal epithelium carcinoma-associated protein 3	1.205
RPL12	60S ribosomal protein L12	1.195
TOR1A	Torsin-1A	1.195
RPL15	60S ribosomal protein L15	1.188
FKBP10	FK506-binding protein 10	1.183
TAGLN	Transgelin	1.179
HBXIP	Hepatitis B virus X-interacting protein	1.179
TPM3	Putative uncharacterized protein DKFZp686J1372	1.171
NCEH1	Neutral cholesterol ester hydrolase 1	1.168
TMEM2	Transmembrane protein 2	1.161
SLC25A5	ADP/ATP translocase 2	1.156
RAN	GTP-binding nuclear protein Ran	1.148
SMC3	Structural maintenance of chromosomes protein 3	1.143
MOS	Proto-oncogene serine/threonine-protein kinase mos	1.135
NDUFS2	NADH dehydrogenase [ubiquinone] iron-sulfur protein 2, mitochondrial	1.131
HM13	Minor histocompatibility antigen H13	1.131
RPL8	60S ribosomal protein L8	1.130
CCDC19	Coiled-coil domain-containing protein 19, mitochondrial	1.119
RPL13	60S ribosomal protein L13	1.117
YIF1B	Protein YIF1B	1.095
RPS13	40S ribosomal protein S13	1.086
TBL2	Transducin beta-like protein 2	1.082
MXRA7	Matrix-remodeling-associated protein 7	1.068
MOGS	Mannosyl-oligosaccharide glucosidase	1.064
SLC25A6	ADP/ATP translocase 3	1.058
RAC1	Ras-related C3 botulinum toxin substrate 1	1.056
ACT	Actin-like protein (Fragment)	1.044
PGAM1	Phosphoglycerate mutase 1	1.041
GOLIM4	Golgi integral membrane protein 4	1.032
DHRS7B	Dehydrogenase/reductase SDR family member 7B	1.026
SSR3	Translocon-associated protein subunit gamma	1.015
DMD	Dystrophin	0.991
SLC25A1	Tricarboxylate transport protein, mitochondrial	0.985
SACM1L	Phosphatidylinositide phosphatase SAC1	0.983

ARL1	ADP-ribosylation factor-like protein 1	0.972
HNRNPU	Heterogeneous nuclear ribonucleoprotein U	0.967
ELOVL1	Elongation of very long chain fatty acids protein 1	0.962
COX5B	Cytochrome c oxidase subunit 5B, mitochondrial	0.956
KDELR2	ER lumen protein retaining receptor 2	0.955
HSP90B1	Endoplasmin	0.952
RPSA	40S ribosomal protein SA	0.950
PIGS	GPI transamidase component PIG-S	0.936
ZDHHC5	Probable palmitoyltransferase ZDHHC5	0.932
RPL18A	60S ribosomal protein L18a	0.921
GCS1	Glucosidase I	0.916
TM9SF2	Transmembrane 9 superfamily member 2	0.915
UNC84B	Protein unc-84 homolog B	0.914
IKIP	IKIP protein (Fragment)	0.911
TOMM22	Mitochondrial import receptor subunit TOM22 homolog	0.900
ALS2CR4	Amyotrophic lateral sclerosis 2 chromosomal region candidate gene 4 protein	0.896
TMED4	Transmembrane emp24 domain-containing protein 4	0.893
TMEM85	Transmembrane protein 85	0.889
CSRP1	Cysteine and glycine-rich protein 1	0.882
PHB2	Prohibitin-2	0.875
TUSC3	Tumor suppressor candidate 3	0.863
RPN1	Dolichyl-diphosphooligosaccharide--protein glycosyltransferase subunit 1	0.845
TMX3	Protein disulfide-isomerase TMX3	0.845
TOMM70A	Mitochondrial import receptor subunit TOM70	0.844
RAB35	Ras-related protein Rab-35	0.843
RAB14	Ras-related protein Rab-14	0.837
TPM4	Tropomyosin alpha-4 chain	0.833
IKIP	Inhibitor of nuclear factor kappa-B kinase-interacting protein	0.830
DBN1	Drebrin	0.823
RAB2A	Ras-related protein Rab-2A	0.823
GGCX	Vitamin K-dependent gamma-carboxylase	0.819
RPS23	40S ribosomal protein S23	0.813
ALG5	Dolichyl-phosphate beta-glucosyltransferase	0.807
ADPGK	ADP-dependent glucokinase	0.800
MGST3	Microsomal glutathione S-transferase 3	0.799
AIFM1	Apoptosis-inducing factor 1, mitochondrial	0.787
ZW10	Centromere/kinetochore protein zw10 homolog	0.786
GLIPR2	Golgi-associated plant pathogenesis-related protein 1	0.771
GOLGA5	Golgin subfamily A member 5	0.766
PPAPDC2	Presqualene diphosphate phosphatase	0.759
MYOF	Myoferlin	0.755
GALNT1	Polypeptide N-acetylgalactosaminyltransferase 1	0.750
EBP	3-beta-hydroxysteroid-Delta(8),Delta(7)-isomerase	0.749
CTAGE5	Cutaneous T-cell lymphoma-associated antigen 5	0.748
COX4I1	Cytochrome c oxidase subunit 4 isoform 1, mitochondrial	0.746
RAB10	Ras-related protein Rab-10	0.730
NSDHL	Sterol-4-alpha-carboxylate 3-dehydrogenase, decarboxylating	0.712
ATP5J2	ATP synthase subunit f, mitochondrial	0.703
TUFM	Elongation factor Tu, mitochondrial	0.696
DNAJC1	DnaJ homolog subfamily C member 1	0.691
LRRC59	Leucine-rich repeat-containing protein 59	0.689
TOMM40	Mitochondrial import receptor subunit TOM40 homolog	0.685
TMEM214	Transmembrane protein 214	0.681
ESYT1	Extended synaptotagmin-1	0.679

CAND1	Cullin-associated NEDD8-dissociated protein 1	0.672
RAB11A	Ras-related protein Rab-11A	0.664
P4HB	Protein disulfide-isomerase	0.657
PIGT	GPI transamidase component PIG-T	0.655
UQCRFS1	Cytochrome b-c1 complex subunit Rieske, mitochondrial	0.654
UQCRFS1	Cytochrome b-c1 complex subunit Rieske, mitochondrial	0.654
CHCHD3	Coiled-coil-helix-coiled-coil-helix domain containing 3, isoform CRA_d	0.646
RAB1B	Ras-related protein Rab-1B	0.633
RPLP0	60S acidic ribosomal protein P0	0.627
DDRGK1	Putative uncharacterized protein DDRGK1	0.626
ESYT2	Extended synaptotagmin-2	0.619
ATAD3A	ATPase family, AAA domain containing 3A (Fragment)	0.619
IMMT	Putative uncharacterized protein IMMT	0.618
PFN1	Profilin-1	0.616
SLC25A24	Calcium-binding mitochondrial carrier protein SCaMC-1	0.612
FAM176B	Protein FAM176B	0.611
SEC22B	Vesicle-trafficking protein SEC22b	0.607
DAD1	Dolichyl-diphosphooligosaccharide--protein glycosyltransferase subunit DAD1	0.597
C11orf59	RhoA activator C11orf59	0.594
LSS	Lanosterol synthase	0.587
TTC35	Tetratricopeptide repeat protein 35	0.585
RPS18	40S ribosomal protein S18	0.585
LPCAT1	Lysophosphatidylcholine acyltransferase 1	0.583
HSD17B12	Estradiol 17-beta-dehydrogenase 12	0.577
RPS17	40S ribosomal protein S17	0.575
PRAF2	PRA1 family protein 2	0.575
RPLP0	RPLP0 protein	0.575
MAGT1	Magnesium transporter protein 1	0.568
RAB7A	Ras-related protein Rab-7a	0.563
FKBP11	FK506-binding protein 11	0.558
FLJ00144	FLJ00144 protein (Fragment)	0.555
NSF	Vesicle-fusing ATPase	0.550
PDIA6	Protein disulfide-isomerase A6	0.545
BET1	BET1 homolog	0.531
TMED9	Transmembrane emp24 domain-containing protein 9	0.530
PTPN1	Tyrosine-protein phosphatase non-receptor type 1	0.526
PDCD6	Programmed cell death protein 6	0.521
RPL3L	60S ribosomal protein L3-like	0.517
TAX1BP3	Tax1-binding protein 3	0.507
YWHAB	14-3-3 protein beta/alpha	0.497
TECR	Trans-2,3-enoyl-CoA reductase	0.493
MFSD10	Major facilitator superfamily domain-containing protein 10	0.490
SEC11A	Signal peptidase complex catalytic subunit SEC11A	0.490
ATL3	Atlastin-3	0.488
MAPKSP1	Mitogen-activated protein kinase scaffold protein 1	0.481
USO1	General vesicular transport factor p115	0.476
FARSA	Phenylalanyl-tRNA synthetase alpha chain	0.472
KRT18	Keratin, type I cytoskeletal 18	0.467
SEC61A1	Protein transport protein Sec61 subunit alpha isoform 1	0.462
CNN2	Calponin-2	0.459
RETSAT	All-trans-retinol 13,14-reductase	0.452
RPL23A	60S ribosomal protein L23a	0.447
PTPLAD1	Protein tyrosine phosphatase-like protein PTPLAD1	0.443
RPS3	40S ribosomal protein S3	0.440
KPNB1	Importin subunit beta-1	0.438

IMMT	Mitochondrial inner membrane protein	0.437
UQCRH	Cytochrome b-c1 complex subunit 6, mitochondrial	0.433
COL6A3	Collagen alpha-3(VI) chain	0.432
NDUFV1	NADH dehydrogenase [ubiquinone] flavoprotein 1, mitochondrial	0.431
PRDX6	Peroxiredoxin-6	0.429
GANAB	Neutral alpha-glucosidase AB	0.424
SPCS2	Signal peptidase complex subunit 2	0.414
TMED7	Transmembrane emp24 domain-containing protein 7	0.413
DSTN	Destrin	0.403
LEPRE1	Prolyl 3-hydroxylase 1	0.387
DGAT1	Diacylglycerol O-acyltransferase 1	0.384
PRDX4	Peroxiredoxin-4	0.379
STT3B	Dolichyl-diphosphooligosaccharide--protein glycosyltransferase subunit STT3B	0.376
NDUFA8	NADH dehydrogenase [ubiquinone] 1 alpha subcomplex subunit 8	0.373
ZMPSTE24	CAAX prenyl protease 1 homolog	0.370
EFR3A	Protein EFR3 homolog A	0.368
RPS2	40S ribosomal protein S2	0.366
IGFBP5	Insulin-like growth factor-binding protein 5	0.365
PRKAR1A	cAMP-dependent protein kinase type I-alpha regulatory subunit	0.361
TMEM43	Transmembrane protein 43	0.358
GPX8	Glutathione peroxidase	0.356
EPHX1	Epoxide hydrolase 1	0.354
PRKCSH	Glucosidase 2 subunit beta	0.346
CYC1	Cytochrome c1, heme protein, mitochondrial	0.331
COPE	Coatomer subunit epsilon	0.325
ARL8B	ADP-ribosylation factor-like protein 8B	0.321
YWHAQ	14-3-3 protein theta	0.317
NDUFB9	NADH dehydrogenase [ubiquinone] 1 beta subcomplex subunit 9	0.313
LRRC1	Leucine-rich repeat-containing protein 1	0.292
HSPA1L	Heat shock 70 kDa protein 1L	0.267
PBXIP1	Pre-B-cell leukemia transcription factor-interacting protein 1	0.262
ACTA2	Actin, aortic smooth muscle	0.248
MBLAC2	Metallo-beta-lactamase domain-containing protein 2	0.248
LMNA	Lamin-A/C	0.238
ANXA11	Annexin A11	0.236
RPN2	Dolichyl-diphosphooligosaccharide--protein glycosyltransferase subunit 2	0.229
MGST1	Microsomal glutathione S-transferase 1	0.226
ACTG1	Actin, cytoplasmic 2	0.221
RPS27A	Ubiquitin	0.212
RCN1	Reticulocalbin-1	0.209
ATP5O	ATP synthase subunit O, mitochondrial	0.206
ILK	Integrin-linked protein kinase	0.204
TMED5	TMED5 protein	0.198
LMAN2	Vesicular integral-membrane protein VIP36	0.196
MLEC	Malectin	0.194
STXBP3	Syntaxin-binding protein 3	0.191
PRPH	Peripherin	0.186
LEMD2	LEM domain-containing protein 2	0.178
HNRNPK	Heterogeneous nuclear ribonucleoprotein K	0.176
CDC25A	M-phase inducer phosphatase 1	0.170
ACTC1	Actin, alpha cardiac muscle 1	0.164
RPL18	60S ribosomal protein L18	0.156
RPS7	40S ribosomal protein S7	0.142
RPL7A	60S ribosomal protein L7a	0.142

SEC61G	Protein transport protein Sec61 subunit gamma	0.134
BAK1	Bcl-2 homologous antagonist/killer	0.131
TRAM1	Translocating chain-associated membrane protein 1	0.130
HSP90AB1	Heat shock protein HSP 90-beta	0.129
TCIRG1	V-type proton ATPase 116 kDa subunit a isoform 3	0.128
RPL24	60S ribosomal protein L24	0.121
SCARF2	Scavenger receptor class F member 2	0.120
C5orf43	UPF0542 protein C5orf43	0.119
RAB9A	Ras-related protein Rab-9A	0.109
CYB5B	Putative uncharacterized protein DKFZp686M0619 (Fragment)	0.102
LNP	Protein lunapark	0.089
MFF	Mitochondrial fission factor	0.084
S100A13	Protein S100-A13	0.078
TOR1AIP1	Torsin-1A-interacting protein 1	0.075
NDUFA9	NADH dehydrogenase [ubiquinone] 1 alpha subcomplex subunit 9, mitochondrial	0.069
LMAN1	Protein ERGIC-53	0.066
MPDU1	Mannose-P-dolichol utilization defect 1 protein	0.059
RAB5C	Ras-related protein Rab-5C	0.053
MOXD1	DBH-like monooxygenase protein 1	0.052
PDLIM7	PDZ and LIM domain protein 7	0.039
FKBP8	FK506-binding protein 8	0.029
UBE2N	Ubiquitin-conjugating enzyme E2 N	0.026
COX5A	Cytochrome c oxidase subunit 5A, mitochondrial	0.023
TMEM179B	Transmembrane protein 179B	0.016
RPL14	RPL14 protein	0.008
ABHD12	Monoacylglycerol lipase ABHD12	0.007
CYP51A1	Lanosterol 14-alpha demethylase	0.006
SCCPDH	Probable saccharopine dehydrogenase	-0.004
PPIC	Peptidyl-prolyl cis-trans isomerase C	-0.013
MYO1D	Myosin-Id	-0.014
FAM3C	Protein FAM3C	-0.014
ATP13A1	Probable cation-transporting ATPase 13A1	-0.023
ARPC2	Actin-related protein 2/3 complex subunit 2	-0.026
CCT3	T-complex protein 1 subunit gamma	-0.030
RPL5	60S ribosomal protein L5	-0.031
HSPA1A	Heat shock 70 kDa protein 1	-0.033
OSTC	Oligosaccharyltransferase complex subunit OSTC	-0.041
YWHAG	14-3-3 protein gamma	-0.045
NAPA	Alpha-soluble NSF attachment protein	-0.047
CISD1	CDGSH iron sulfur domain-containing protein 1	-0.047
PRKCDBP	Protein kinase C delta-binding protein	-0.056
H3F3A	Histone H3.3	-0.062
HP1BP3	Heterochromatin protein 1-binding protein 3	-0.066
SPNS1	Protein spinster homolog 1	-0.070
DDOST	Dolichyl-diphosphooligosaccharide--protein glycosyltransferase 48 kDa subunit	-0.075
MT-CO2	Cytochrome c oxidase subunit 2	-0.081
CISD2	CDGSH iron sulfur domain-containing protein 2	-0.085
COPG	Coatomer subunit gamma	-0.089
ERO1L	ERO1-like protein alpha	-0.090
ASPH	Aspartyl/asparaginyl beta-hydroxylase	-0.095
KRT7	Keratin, type II cytoskeletal 7	-0.095
DDAH2	N(G),N(G)-dimethylarginine dimethylaminohydrolase 2	-0.096
APMAP	Adipocyte plasma membrane-associated protein	-0.099
RPS10	40S ribosomal protein S10	-0.104

IDH1	Isocitrate dehydrogenase [NADP] cytoplasmic	-0.104
LMNB1	Lamin-B1	-0.108
CTSA	Lysosomal protective protein	-0.110
SKP1	S-phase kinase-associated protein 1	-0.118
FASN	Fatty acid synthase	-0.120
SQSTM1	Sequestosome-1	-0.124
PGRMC1	Membrane-associated progesterone receptor component 1	-0.127
ANO6	Anoctamin-6	-0.140
ARL6IP6	ADP-ribosylation factor-like protein 6-interacting protein 6	-0.143
NDUFA12	NADH dehydrogenase [ubiquinone] 1 alpha subcomplex subunit 12	-0.148
HMOX1	Heme oxygenase 1	-0.165
NDUFS8	NADH dehydrogenase [ubiquinone] iron-sulfur protein 8, mitochondrial	-0.166
NDUFA13	NADH dehydrogenase [ubiquinone] 1 alpha subcomplex subunit 13	-0.174
PEX14	Peroxisomal membrane protein PEX14	-0.174
TAPBP	Tapasin	-0.175
GNB2	Guanine nucleotide-binding protein G(I)/G(S)/G(T) subunit beta-2	-0.179
RPL28	60S ribosomal protein L28	-0.179
RER1	Protein RER1	-0.181
HMOX2	Heme oxygenase 2	-0.189
RPS20	40S ribosomal protein S20	-0.190
VIM	Vimentin	-0.213
NNMT	Nicotinamide N-methyltransferase	-0.214
SDF4	45 kDa calcium-binding protein	-0.214
ACTR1A	Alpha-centractin	-0.219
TMEM30A	Cell cycle control protein 50A	-0.223
HNRNPF	Heterogeneous nuclear ribonucleoprotein F	-0.226
ATP5A1	ATP synthase subunit alpha, mitochondrial	-0.229
ATP5H	ATP synthase subunit d, mitochondrial	-0.231
TMEM87A	Transmembrane protein 87A	-0.240
EEF1A1	Elongation factor 1-alpha 1	-0.248
RPS6	40S ribosomal protein S6	-0.250
VDAC3	Voltage-dependent anion-selective channel protein 3	-0.251
PLEKHO2	Pleckstrin homology domain-containing family O member 2	-0.255
IPO7	Importin-7	-0.256
TMPO	Lamina-associated polypeptide 2, isoforms beta/gamma	-0.257
SLC25A3	Phosphate carrier protein, mitochondrial	-0.257
VDAC2	Voltage-dependent anion-selective channel protein 2	-0.258
MYH9	Myosin-9	-0.266
PI4K2A	Phosphatidylinositol 4-kinase type 2-alpha	-0.275
TMEM87B	Transmembrane protein 87B	-0.279
TMEM173	Transmembrane protein 173	-0.284
RPL6	60S ribosomal protein L6	-0.285
RPS9	40S ribosomal protein S9	-0.292
ILVBL	Acetolactate synthase-like protein	-0.293
BAG2	BAG family molecular chaperone regulator 2	-0.306
MTCH1	Mitochondrial carrier homolog 1	-0.310
ACAM	Adipocyte adhesion molecule	-0.314
RPL23	60S ribosomal protein L23	-0.317
FARP1	FERM, RhoGEF and pleckstrin domain-containing protein 1	-0.320
RPL4	60S ribosomal protein L4	-0.348
KIAA1609	cDNA FLJ75055	-0.357
USE1	Vesicle transport protein USE1	-0.359
RPL13A	60S ribosomal protein L13a	-0.360
HSP90AA1	Heat shock protein HSP 90-alpha	-0.372
PTRF	Polymerase I and transcript release factor	-0.381
ACTN1	Alpha-actinin-1	-0.385

RPLP2	60S acidic ribosomal protein P2	-0.396
VKORC1	Vitamin K epoxide reductase complex subunit 1	-0.399
C19orf10	UPF0556 protein C19orf10	-0.400
KRT19	Keratin, type I cytoskeletal 19	-0.402
CYFIP1	Cytoplasmic FMR1-interacting protein 1	-0.405
SEL1L	Protein sel-1 homolog 1	-0.407
SFXN3	Sideroflexin-3	-0.409
TMED5	Transmembrane emp24 domain-containing protein 5	-0.409
FNDC1	Fibronectin type III domain-containing protein 1	-0.417
HLA-B35	MHC class I antigen HLA-B35	-0.427
RPL17	60S ribosomal protein L17	-0.432
MTX1	Metaxin-1	-0.440
UGGT1	UDP-glucose:glycoprotein glucosyltransferase 1	-0.441
SFPQ	Splicing factor, proline- and glutamine-rich	-0.446
BAX	Apoptosis regulator BAX	-0.460
NDUFA3	NADH dehydrogenase [ubiquinone] 1 alpha subcomplex subunit 3	-0.460
VKORC1L1	Vitamin K epoxide reductase complex subunit 1-like protein 1	-0.464
SEPT9	Septin-9	-0.467
PSMD6	26S proteasome non-ATPase regulatory subunit 6	-0.469
C19orf52	Uncharacterized protein C19orf52	-0.498
S100A16	Protein S100-A16	-0.506
YIF1A	Protein YIF1A	-0.520
SPCS3	Signal peptidase complex subunit 3	-0.521
FERMT2	Fermitin family homolog 2	-0.522
TPP1	Tripeptidyl-peptidase 1	-0.529
NNT	NAD(P) transhydrogenase, mitochondrial	-0.530
PSMD11	26S proteasome non-ATPase regulatory subunit 11	-0.531
UQCRC1	Cytochrome b-c1 complex subunit 1, mitochondrial	-0.536
RPL22	60S ribosomal protein L22	-0.537
UQCRQ	Cytochrome b-c1 complex subunit 8	-0.538
SLC30A9	Zinc transporter 9	-0.542
ANXA4	Annexin A4	-0.543
ATP5F1	ATP synthase subunit b, mitochondrial	-0.547
ATP5L	ATP synthase subunit g, mitochondrial	-0.551
SQRDL	cDNA, FLJ93596, highly similar to Homo sapiens sulfide quinone reductase-like (yeast) (SQRDL), mRNA	-0.565
HIST1H1B	Histone H1.5	-0.569
SEPT11	Septin-11	-0.572
RPS24	40S ribosomal protein S24	-0.575
RPS4X	40S ribosomal protein S4, X isoform	-0.576
SEC23A	Protein transport protein Sec23A	-0.583
NDUFS1	NADH-ubiquinone oxidoreductase 75 kDa subunit, mitochondrial	-0.583
LMNB2	Lamin-B2	-0.591
BGN	Biglycan	-0.609
TAP2	Antigen peptide transporter 2	-0.609
MYH10	Myosin-10	-0.626
ARPC3	Actin-related protein 2/3 complex subunit 3	-0.631
PCDHGA4	Protocadherin gamma-A4	-0.641
FAM162A	UPF0389 protein FAM162A	-0.646
GCN1L1	Translational activator GCN1	-0.655
RPL10	60S ribosomal protein L10	-0.658
CCDC56	Coiled-coil domain-containing protein 56	-0.659
NDUFB5	NADH dehydrogenase [ubiquinone] 1 beta subcomplex subunit 5, mitochondrial	-0.659
RPS16	40S ribosomal protein S16	-0.665
ACTN4	Alpha-actinin-4	-0.666

CPNE2	Copine-2	-0.677
MCFD2	Multiple coagulation factor deficiency protein 2	-0.678
ERLIN2	Erlin-2	-0.678
ANO10	Anoctamin-10	-0.678
COX6C	Cytochrome c oxidase subunit 6C	-0.679
HIST1H1C	Histone H1.2	-0.683
EZR	Ezrin	-0.686
HSPA8	Heat shock cognate 71 kDa protein	-0.697
CYB5A	Cytochrome b5	-0.702
CCT5	T-complex protein 1 subunit epsilon	-0.703
RPL7	60S ribosomal protein L7	-0.719
YWHAE	14-3-3 protein epsilon	-0.721
ANXA4	cDNA FLJ52218, highly similar to Annexin A4	-0.721
PNPLA6	Neuropathy target esterase	-0.727
BCAP31	B-cell receptor-associated protein 31	-0.734
ALDH3A2	Fatty aldehyde dehydrogenase	-0.739
RPS11	40S ribosomal protein S11	-0.753
UQCRC2	Cytochrome b-c1 complex subunit 2, mitochondrial	-0.781
LDHA	L-lactate dehydrogenase A chain	-0.790
COL6A1	Collagen alpha-1(VI) chain	-0.793
RALB	Ras-related protein Ral-B	-0.798
NAP1L1	Nucleosome assembly protein 1-like 1	-0.803
MFN2	Mitofusin-2	-0.810
ERVK6	HERV-K_7p22.1 provirus ancestral Pol protein	-0.827
RPS8	40S ribosomal protein S8	-0.829
CCDC109A	Coiled-coil domain-containing protein 109A	-0.834
CAPZA1	F-actin-capping protein subunit alpha-1	-0.837
ACLY	ATP-citrate synthase	-0.841
HIST1H4A	Histone H4	-0.850
WDR1	WD repeat-containing protein 1	-0.852
MYL12A	Myosin regulatory light chain 12A	-0.857
RARS	Arginyl-tRNA synthetase, cytoplasmic	-0.875
RPL21	60S ribosomal protein L21	-0.900
CORO1B	Coronin-1B	-0.901
FLNC	Filamin-C	-0.901
CAPZA2	F-actin-capping protein subunit alpha-2	-0.902
OCC1	Putative overexpressed in colon carcinoma 1 protein	-0.920
BCL2L13	Bcl-2-like protein 13	-0.921
CAP1	Adenylyl cyclase-associated protein	-0.925
RAP2C	Ras-related protein Rap-2c	-0.928
PALLD	Palladin	-0.956
MYADM	Myeloid-associated differentiation marker	-0.967
PDCD6IP	Programmed cell death 6-interacting protein	-0.967
PRDX1	Peroxiredoxin-1	-0.987
PPP2R1A	Serine/threonine-protein phosphatase 2A 65 kDa regulatory subunit A alpha isoform	-0.988
TMEM109	Transmembrane protein 109	-0.990
VCP	Transitional endoplasmic reticulum ATPase	-1.002
ERGIC1	Endoplasmic reticulum-Golgi intermediate compartment protein 1	-1.003
PPIA	Peptidyl-prolyl cis-trans isomerase A	-1.008
SEPT7	Septin-7	-1.012
RASEF	RAS and EF-hand domain-containing protein	-1.025
TMEM119	Transmembrane protein 119	-1.032
UBA1	Ubiquitin-like modifier-activating enzyme 1	-1.032
DAB2	Disabled homolog 2	-1.042
PCYOX1	Prenylcysteine oxidase 1	-1.061

S100A6	Protein S100-A6	-1.068
TMOD3	Tropomodulin-3	-1.075
VAT1	Synaptic vesicle membrane protein VAT-1 homolog	-1.091
ATP5C1	ATP synthase subunit gamma, mitochondrial	-1.120
MAP4	Microtubule-associated protein 4	-1.126
SNTB2	Beta-2-syntrophin	-1.126
EEF2	Elongation factor 2	-1.182
RPL38	60S ribosomal protein L38	-1.198
TUBB6	Tubulin beta-6 chain	-1.214
CHMP6	Charged multivesicular body protein 6	-1.219
COPB1	Coatomer subunit beta	-1.239
TUBB4	Tubulin beta-4 chain	-1.241
RNH1	Ribonuclease inhibitor	-1.244
TUBB2C	Tubulin beta-2C chain	-1.244
TUBB2A	Tubulin beta-2A chain	-1.249
KIF5B	Kinesin-1 heavy chain	-1.250
SLC25A12	Calcium-binding mitochondrial carrier protein Aralar1	-1.253
JAZF1	Putative uncharacterized protein JAZF1	-1.275
GSTP1	Glutathione S-transferase P	-1.285
TUBB	Tubulin beta chain	-1.306
TTN	Titin	-1.309
ACTR3	Actin-related protein 3	-1.322
NARS	Asparaginyl-tRNA synthetase, cytoplasmic	-1.352
MVP	Major vault protein	-1.395
PKM2	Pyruvate kinase isozymes M1/M2	-1.402
PHGDH	D-3-phosphoglycerate dehydrogenase	-1.402
TUBB3	Tubulin beta-3 chain	-1.415
MX2	Interferon-induced GTP-binding protein Mx2	-1.420
CORO1C	Coronin-1C	-1.433
SEPT2	Septin-2	-1.434
ATP6V0D1	V-type proton ATPase subunit d 1	-1.438
EIF5A	Eukaryotic translation initiation factor 5A-1	-1.441
PARK7	Protein DJ-1	-1.463
TMEM138	Transmembrane protein 138	-1.466
GGT5	Gamma-glutamyltransferase 5	-1.474
KRT8	Keratin, type II cytoskeletal 8	-1.513
GNG12	Guanine nucleotide-binding protein G(I)/G(S)/G(O) subunit gamma-12	-1.513
CCT6A	T-complex protein 1 subunit zeta	-1.577
VASP	Vasodilator-stimulated phosphoprotein	-1.589
TUBA1B	Tubulin alpha-1B chain	-1.594
GPD2	Glycerol-3-phosphate dehydrogenase, mitochondrial	-1.602
FLJ44635	TPT1-like protein	-1.604
TUBA1C	Tubulin alpha-1C chain	-1.614
RANBP1	Ran-specific GTPase-activating protein	-1.615
TUBA1A	Tubulin alpha-1A chain	-1.619
SEC31A	Protein transport protein Sec31A	-1.689
PLIN3	Perilipin-3	-1.703
FTL	Ferritin light chain	-1.715
TAGLN2	Transgelin-2	-1.777
KCTD12	BTB/POZ domain-containing protein KCTD12	-1.805
MAVS	Mitochondrial antiviral-signaling protein	-1.806
GARS	Glycyl-tRNA synthetase	-1.843
RPS5	40S ribosomal protein S5	-1.875
MYL6	Myosin light polypeptide 6	-1.887
KRT2	Keratin, type II cytoskeletal 2 epidermal	-1.912
ALDOA	Fructose-bisphosphate aldolase A	-2.017

KRT10	Keratin, type I cytoskeletal 10	-2.022
ENDOD1	Endonuclease domain-containing 1 protein	-2.074
ATP11C	Probable phospholipid-transporting ATPase IG	-2.083
AHNAK2	Protein AHNAK2	-2.198
VTA1	Vacuolar protein sorting-associated protein VTA1 homolog	-2.215
SLC25A11	Mitochondrial 2-oxoglutarate/malate carrier protein	-2.218
MIF	Macrophage migration inhibitory factor	-2.223
GAPDH	Glyceraldehyde-3-phosphate dehydrogenase	-2.250
IPO9	Importin-9	-2.256
CCT2	T-complex protein 1 subunit beta	-2.257
hCG_1787790	Ribosomal protein L1	-2.388
KHDRBS1	KH domain-containing, RNA-binding, signal transduction-associated protein 1	-2.424
CYB5R1	NADH-cytochrome b5 reductase 1	-2.429
COPB2	Coatomer subunit beta~	-2.440
CCT8	T-complex protein 1 subunit theta	-2.503
PTGFRN	Prostaglandin F2 receptor negative regulator	-2.525
COPA	Coatomer subunit alpha	-2.527
MAPK1	Mitogen-activated protein kinase 1	-2.533
PSMD12	26S proteasome non-ATPase regulatory subunit 12	-2.552
GPI	Glucose-6-phosphate isomerase	-2.640
KRT5	Keratin, type II cytoskeletal 5	-2.648
AGK	Acylglycerol kinase, mitochondrial	-2.659
KDM5B	Lysine-specific demethylase 5B	-2.661
KRT14	Keratin, type I cytoskeletal 14	-2.661
KANK2	KN motif and ankyrin repeat domain-containing protein 2	-2.706
FAM114A1	Protein NOXP20	-2.717
TMTC3	Transmembrane and TPR repeat-containing protein 3	-2.735
DCTN2	Dynactin subunit 2	-2.748
GFPT2	Glucosamine--fructose-6-phosphate aminotransferase [isomerizing] 2	-2.783
LDHB	L-lactate dehydrogenase B chain	-2.819
IPO5	Importin-5	-2.882
CAPN5	Calpain-5	-2.949
MAPK4	Mitogen-activated protein kinase 4	-2.955
FTL	Ferritin	-3.000
KRT9	Keratin, type I cytoskeletal 9	-3.017
MDH2	Malate dehydrogenase, mitochondrial	-3.077
GSN	Gelsolin	-3.097
PTGFRN	PTGFRN protein (Fragment)	-3.133
RAI14	Ankycorbin	-3.165
KRT6C	Keratin, type II cytoskeletal 6C	-3.220
SLC27A3	Long-chain fatty acid transport protein 3	-3.231
TKT	Transketolase	-3.281
ARHGDIA	Rho GDP-dissociation inhibitor 1	-3.537
JAK1	Tyrosine-protein kinase JAK1	-3.558
PLS3	Plastin-3	-3.569
CAMK2D	Calcium/calmodulin-dependent protein kinase type II delta chain	-3.576
CRMP1	Dihydropyrimidinase-related protein 1	-3.610
ZC3H18	Zinc finger CCCH domain-containing protein 18	-3.747
LASP1	LIM and SH3 domain protein 1	-3.760
CSE1L	Exportin-2	-3.839
KRT6B	Keratin, type II cytoskeletal 6B	-3.882
ALB	Serum albumin	-3.887
DCTN1	Dynactin subunit 1	-4.264
PFKP	6-phosphofructokinase type C	-4.335
G6PD	Glucose-6-phosphate 1-dehydrogenase	-4.336

PGK1	Phosphoglycerate kinase 1	-4.399
CAMK2B	Calcium/calmodulin-dependent protein kinase type II beta chain	-4.444
CAMK2A	Calcium/calmodulin-dependent protein kinase type II alpha chain	-4.524
CRYAB	Alpha-crystallin B chain	-4.577
C14orf49	Nesprin-3	-4.613
STAT1	Signal transducer and activator of transcription 1-alpha/beta	-4.654
TMEM14C	Transmembrane protein 14C	-4.671
CYP1B1	Cytochrome P450 1B1	-4.701
LGALS3	Galectin-3	-4.816
ABCA8	ABCA8 protein	-4.880
TPI1	Triosephosphate isomerase	-4.938
UNC84A	Protein unc-84 homolog A	-5.030
TRMT2B	tRNA (uracil-5-)-methyltransferase homolog	-5.037
HSPB6	Heat shock protein beta-6	-5.110
KRT16	Keratin, type I cytoskeletal 16	-5.115
UGDH	UDP-glucose 6-dehydrogenase	-5.704
